# Self-assembled multidye-sensitized erbium single molecules for boosting energy transfer light upconversion in solution†

**DOI:** 10.1039/d5dt00438a

**Published:** 2025-05-29

**Authors:** Filipe Alves, Inès Taarit, Laure Guénée, Claude Piguet

**Affiliations:** a Department of Inorganic and Analytical Chemistry, University of Geneva 30 quai E. Ansermet CH-1211 Geneva 4 Switzerland Claude.Piguet@unige.ch; b Laboratory of Crystallography, University of Geneva 24 quai E. Ansermet CH-1211 Geneva 4 Switzerland

## Abstract

Efficient near-infrared (NIR) to visible (VIS) light upconversion should combine large absorption coefficients *ε*_NIR_ with very large quantum yields *ϕ*^UC^ so that the overall brightness *B*^UC^ = *ε*_NIR_·*ϕ*^UC^ is maximum. Relying on linear optics, several photons are collected by strongly absorbing dyes, stored on long-lived intermediate excited states and finally piled up using mechanisms of simple or double operator natures. The miniaturization to implement detectable linear light upconversion in a single molecule is challenging because of the existence of the thermal vibrational bath, which increases non-radiative relaxation and limits quantum yields to 10^−9^ ≤ *ϕ*^UC^ ≤ 10^−6^. An acceptable brightness thus requires the connection of a maximum of cationic cyanine dyes around trivalent lanthanide luminophores. Taking advantage of the thermodynamic benefit brought by strict self-assembly processes, three cationic IR-780 dyes could be arranged around a single Er(iii) cation in the trinuclear [ZnErZn(L5)_3_]^10+^ triple-stranded helicate. NIR excitation at 801 nm in acetonitrile at room temperature induces light upconversion *via* the energy transfer upconversion (ETU) mechanism. The final green Er(^2^H_11/2_,^4^S_3/2_ → ^4^I_15/2_) emission with *ϕ*^UC^ = 3.6 × 10^−8^ shows a record brightness of *B*^UC^ = 2.8 × 10^−2^ M^−1^ cm^−1^ (*P*_exc_ = 25 W cm^−2^) for a molecular-based upconversion process.

## Abstract 2

The virtues of self-assembly force the connection of three cationic cyanine dyes around a trivalent erbium emitter. Upconverted green emission is observed upon near-infrared excitation of a single molecule in solution at room temperature.

## Graphical abstract

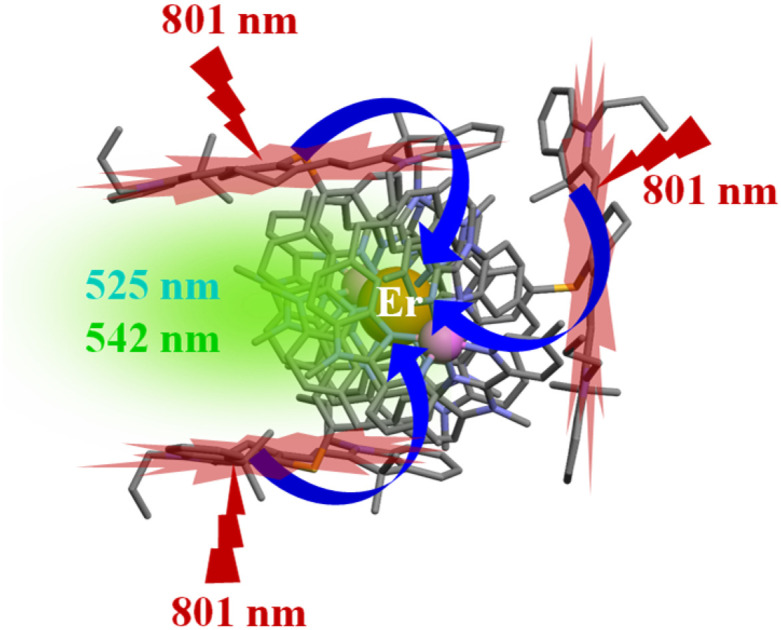

## Introduction

Among the different options to induce anti-Stokes processes,^1–11^ efficient light upconversion upon reasonable excitation intensities, often referred to as upconversion (UC), mainly relies on Bloembergen's strategy, which exploits linear optics.^2,12,13^ The existence of long-lived intermediate excited states is thus crucial for implementing the successive excitations required for piling several low-energy photons and finally reaching a high-energy excited state able to radiatively relax to states of lower energies (Fig. S1 and S2†). An obvious choice considers long-lived triplet excited states found in main group aromatic molecules as intermediate excited relays, followed by triplet–triplet annihilation upconversion involving the diffusion and collision of two independent molecules to reach the final emissive singlet excited state.^3–5,7,8,10^ However, this intermolecular strategy does not fit the criteria for being considered as a discrete molecular process, the subject of the present contribution.^14,15^ The second choice takes advantage of scales of electronic terms produced by interelectronic interactions in open-shell d-block (d^*n*^, *n* = 1–9) and f-block (f^*n*^, *n* = 1–13) metals, according to which these centers are deprived from vibrational-based non-radiative relaxation processes *via* their integration into low-phonon ionic solids or nanoparticles, thus providing long-lived intermediate excited relays.^2,11,16–19^ Again, miniaturization to reach single isolated (supra)molecules based on the latter ionic/polymeric materials is not trivial. Inspired by the excited-state absorption (ESA; Fig. S1a†) and the energy transfer upconversion (ETU; Fig. S1b†) implemented in doped solids, coordination chemists were, however, able to design some rare (supra)molecular complexes exhibiting UC processes in solution under reasonable incident excitation power (*P*_exc_ < 30 W cm^−2^) and using real intermediate excited states.^20–29^ Additionally, cooperative sensitization upconversion (CSU; Fig. S2a†)^30–36^ and cooperative luminescence (CL; Fig. S2b†),^33,36,37^ which consider quasi-virtual pair levels,^38^ have been recently described. These pioneer efforts aiming at implementing these four upconversion mechanisms for molecules in solution are regularly reviewed.^14,15,39–42^ Some reports of solid-state UC recorded for mixtures of co-crystallized coordination complexes, coordination polymers or clusters^43–57^ deserve mention for the sake of comprehensiveness of this introduction despite (i) the assignment of the UC mechanism to the single molecular nature is debatable and (ii) the statistically doped character of the multimetallic samples developed for ETU or CSU processes.

**Fig. 1:**
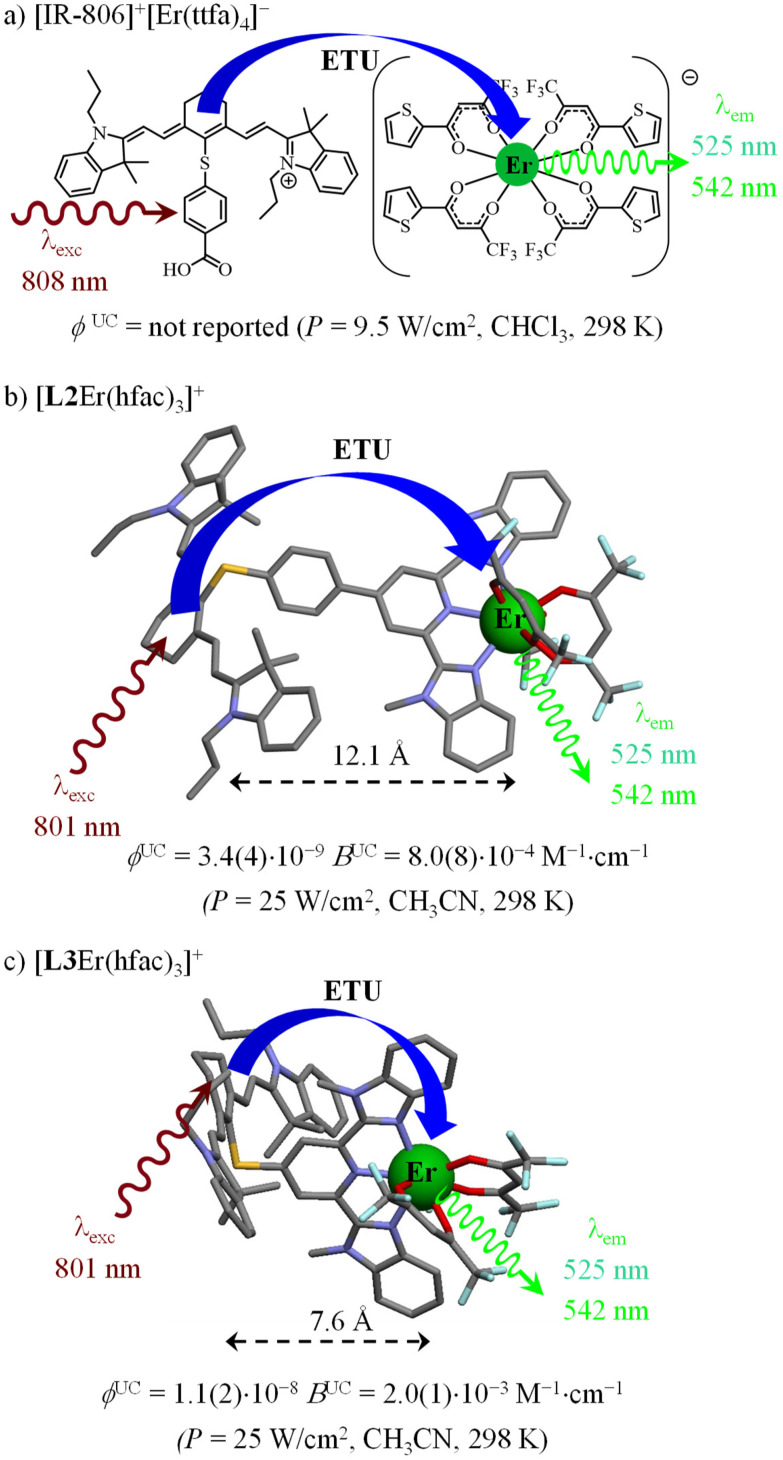
Dye-sensitized ETU implemented in (a) [IR-806]^+^[Er(ttfa)_4_]^−^ ion pairs^65^ and (b and c) single molecular complexes [L2Er(hfac)_3_]^+^ and [L3Er(hfac)_3_]^+^ in solution (CCDC 2091959 for [L2Er(hfac)_2_(CF_3_CO_2_)]^+^ (ref. 28) and CCDC 2238348 for [L3Er(hfac)_2_(CF_3_CO_2_)]^+^ (ref. 29)). Color codes: C = grey, N = dark blue. O = red, F = light blue; S = yellow. The chemical structures of ligands [L2]^+^ and [L3]^+^ are shown in Scheme 1.

Whatever the exact mechanism is, molecular-based near-infrared (NIR) to visible (VIS) UC in solution shows low quantum yields, of the order of 10^−9^ ≤ *ϕ*^UC^ ≤ 10^−6^ (normalized at *P* = 25 W cm^−2^), due to unavoidable large and penalizing non-radiative relaxation processes. Significant improvements should consider balancing the low UC quantum yield (*ϕ*^UC^) by large NIR absorption cross-sections (*ε*_NIR_) to finally provide acceptable UC brightness *B*^UC^ = *ε*_NIR_·*ϕ*^UC^*via* the antenna effect,^58^ a procedure successfully used in solid-state materials upon grafting polymethine dyes^59^ onto the surface of solid UC nanoparticles.^60–64^ At the molecular level, combining a cyanine dye, taken as a sensitizer for Ln-based UC *via* the ETU mechanism, has been first described for [IR-806]^+^[Er(ttfa)_4_]^−^ ion pairs, which are assumed to be formed upon simple mixing in chloroform (Fig. 1a).^65^ As the UC quantum yield *ϕ*^UC^ is proportional to excitation rate constant *k*^exc^_S_,^15^ which in turn is proportional the absorption coefficient *ε*^*m*→*n*^_S_ (eqn (1)),^66–68^ the target brightness *B*^UC^ = *ε*_NIR_·*ϕ*^UC^ benefits twice from the large *ε*_NIR_ absorption coefficient of the cyanine dye (*ε*_NIR_ > 10^5^ M^−1^ cm^−1^):1
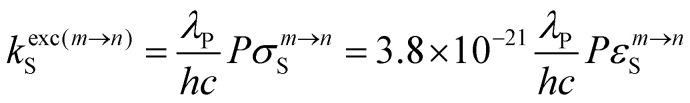


In eqn (1), *λ*_P_ is the pump wavelength (in cm), *P* is the incident pump intensity (in W cm^−2^), *σ*^*m*→*n*^_A_ is the absorption cross section (in cm^2^) of the sensitizer-centered *m* → *n* transition related to the decadic molar absorption coefficient *ε*^*m*→*n*^ (in M^−1^ cm^−1^) according to *σ*^*m*→*n*^ = 3.8 × 10^−21^*ε*^*m*→*n*^, *h* is the Planck constant (in J s) and *c*, the speed of light in vacuum (in cm s^−1^). The covalent connection of a cationic cyanine sensitizer to an erbium activator to give stable and characterized molecular complexes in solution has been reported for [L2Er(hfac)_3_]^+^ (Fig. 1b)^28^ and [L3Er(hfac)_3_]^+^ (Fig. 1c),^29^ which display record brightnesses for molecular UC in solution. The decrease of the sensitizer–activator distance in going from [L2Er(hfac)_3_]^+^ to [L3Er(hfac)_3_]^+^ boosts the UC quantum yield and associated brightness by a factor of three.

**Fig. 2:**
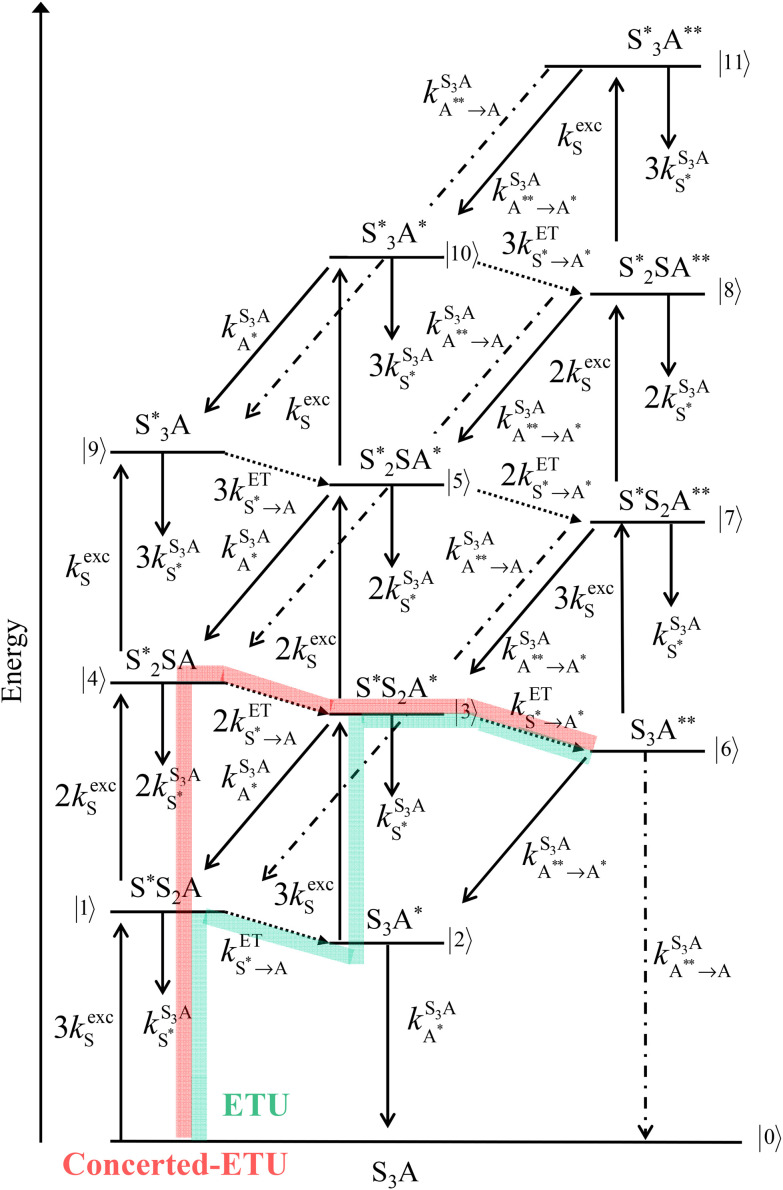
Kinetic twelve-level diagram showing the ETU mechanism programmed in a S_3_A single (supra)molecule (S = sensitizer, A = acceptor) and highlighting the two dominant ETU and concerted-ETU mechanisms.

Further gain can be predicted upon increasing the number of sensitizers per activator in a single S_*n*_A (supra)molecular assembly (illustrated for *n* = 3 in Fig. 2). Beyond the predicted improvement by a factor *n*^2^ of brightness *B*^UC^ (green pathway in Fig. 2), the possibility to accumulate excitations on the sensitizer provides a concomitant and supplementary mechanism, referred to as concerted-ETU (red pathway in Fig. 2),^15^ which may improve molecular UC when long-lived excited states are available on the sensitizers.^26,27^

**Scheme 1:**
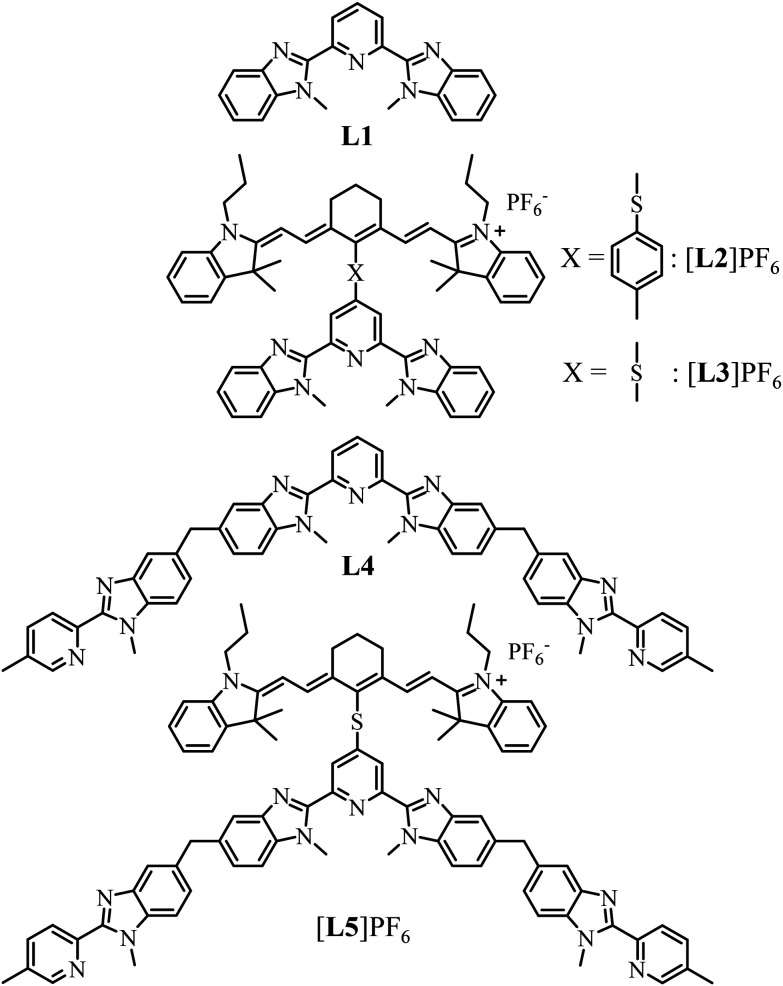
Chemical structures of ligands discussed in this work.

Accordingly, it appears both trivial and appealing to increase the number of cyanine-bearing ligands per erbium activator in a target [Er(L3)_*n*_]^(3+*n*)+^ (*n* = 2–3) molecular complex inspired by [L3Er(hfac)_3_]^+^.^29^ However, the stepwise increase of the positive charges brought by the connected dye-grafted ligands destabilizes the formation of the target complex in solution. Thus, we report below our effort to decipher the coulombic limits for the formation of multi-dye [Er(L3)_*n*_]^(3+*n*)+^ assemblies in solution, while taking the less charged [Ln(L1)_*n*_]^3+^ analogues as references. Forcing three cationic dyes to approach a trivalent Er^3+^ activator for designing a stable complex thus appeared only possible with the help of additional favorable contributions arising from multi-component interactions implemented in thermodynamic self-assemblies.^69–71^ Connecting the cyanine dye to the segmental ligand L4 provided cationic [L5]^+^ (Scheme 1), which is explored for (i) the quantitative formation of the stable multi-dye triple-stranded helicate [ZnErZn(L5)_3_]^10+^ and (ii) the ultimate molecular-based NIR to green light upconverter provided by the latter assembly.

## Results and discussion

### The thermodynamic limits of successive intermolecular association reactions for [Ln(L3)_*n*_]^(3+*n*)+^ (Ln = Eu, Y; *n* = 1–3) in solution

Three compact cyanine dye-grafted tridentate 2,6-bis(benzimidazole) cationic ligands [L3]^+^ (Scheme 1)^29^ were reacted with trivalent lanthanide Ln^3+^ according to equilibria (2)–(4):2
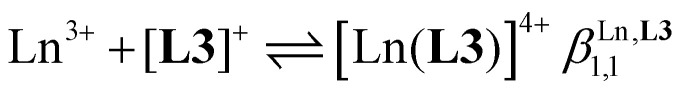
3
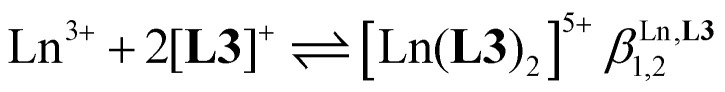
4
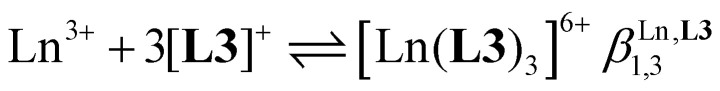


Spectrophotometric titrations of the ligand [L3]PF_6_ (2 × 10^−5^ M) with Eu(CF_3_SO_3_)_3_ (1.1 × 10^−4^ M) in dry acetonitrile exhibited stepwise changes in the UV part of the absorption spectra, which reflect the structural *trans* → *cis* reorganization of the benzimidazole–pyridine units (bzpy) upon complexation (Fig. S3†).^72^ An end point was detected for Eu/L3 = 1 ratio together with some inflexion around Eu/L3 = 0.5 (Fig. S4†). Evolving factor analysis^73–75^ confirmed the existence of only three UV-absorbing species corresponding to equilibria (2)–(3). Non-linear least-square fitting of the spectrophotometric data^76–78^ provided two rough stability constants 
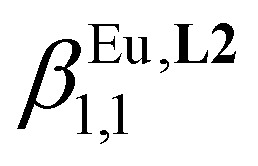
 and 
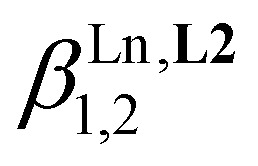
 (Table 1, column 2) together with acceptable reconstructed absorption spectra (Fig. S5b†). Based on the latter thermodynamic association constants, only 80% of the ligand speciation exists in the form [Eu(L3)_2_]^5+^ at 1 : 2 stoichiometric ratio when the total ligand concentration amounts to 2 × 10^−3^ M (Fig. S6†). This explains the non-detection of the desired 1 : 3 complex [Eu(L3)_3_]^6+^ during the spectrophotometric titration conducted at 2 × 10^−5^ M. Higher concentrations can be investigated using ^1^H NMR techniques and titrations of [L3]^+^ (0.5 mM) upon stepwise additions of Eu(CF_3_SO_3_)_3_ (Appendix 1, Fig. A1-1 and A1-2†) or Y(CF_3_SO_3_)_3_ (Appendix 1, Fig. A1-3 and A1-4†) in CD_3_CN at 298 K confirmed the formation of [Ln(L3)]^4+^ and [Ln(L3)_2_]^5+^, together with the formation of traces of [Ln(L3)_3_]^6+^. Non-linear least-squares fits of the binding isotherms with the help of equilibria (2)–(4) provided the stability constants 
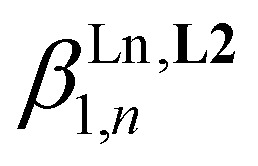
 (*n* = 1–3) gathered in Table 1 (columns 3 and 4; see Appendix 1† for the detailed procedure). In agreement with the operation of the anti-electrostatic trend along the lanthanide series for L1,^79,80^ ligand [L3]^+^ also prefers mid-range metals with 
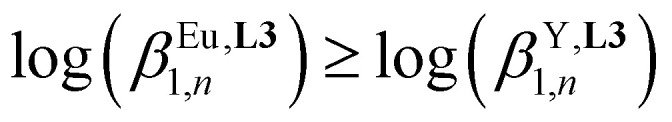
 (Table 1, columns 3 and 4). Comparing the affinities of L1 and [L3]^+^ for trivalent lanthanides with similar ionic radii reveals a striking decrease in affinity with 
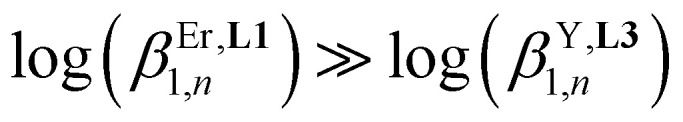
 (Table 1, columns 4 and 5), culminating (eight orders of magnitude) for the balance between triple-helical [Er(L1)_3_]^3+^ and [Y(L3)_3_]^6+^ complexes.

**Table 1:** Thermodynamic stability constants 
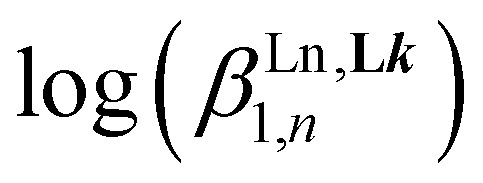
 of the tridentate ligands [L3]^+^ and L1 with Ln(CF_3_SO_3_)_3_ in CD_3_CN at 298 K

Method	Spectrophotometry	NMR	NMR	Spectrophotometry
Ligand	[L3]^+^	[L3]^+^	[L3]^+^	L1
Metal	Eu(CF_3_SO_3_)_3_	Eu(CF_3_SO_3_)_3_	Y(CF_3_SO_3_)_3_	Er(CF_3_SO_3_)_3_
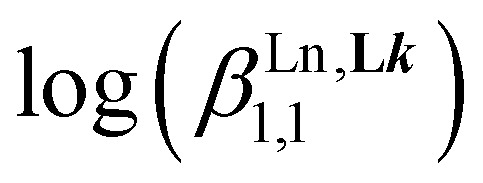	6.3(7)	7.9(4)	7.8(7)	9.2(1)
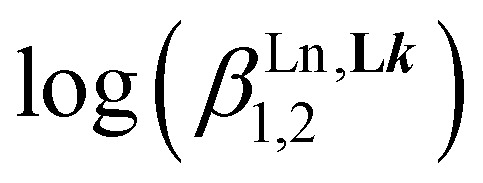	10.3(9)	12.8(6)	10.8(1.0)	16.5(3)
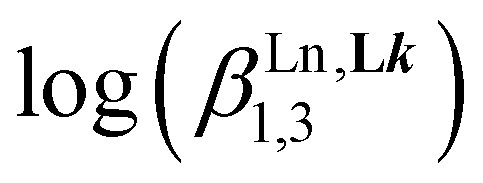	—	14.8(7)	12.7(1.1)	20.9(3)
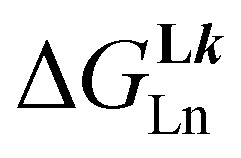 /kJ mol^−1^ a	—	−40.8(1.5)	−36.9(3.9)	−49(1)
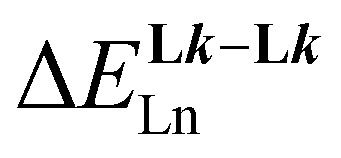 /kJ mol^−1^ b	—	14.9(1.1)	15.3(3.2)	11.6(1.5)
Ref.	This work	This work	This work	72

a Intermolecular ligand–metal affinity 
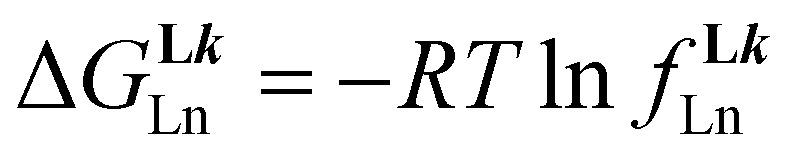
 (see Appendix 1†).

b Interligand interactions 
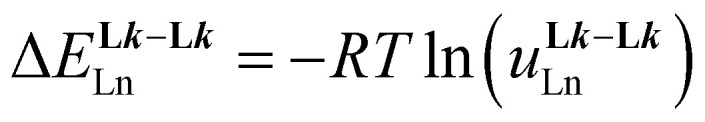
 (see Appendix 1†).

A didactic way of comparing the thermodynamic behaviors of L1 and [L3]^+^ relies on the site binding model (Appendix 1 in the ESI†),^80,81^ from which a free energy of intermolecular ligand–metal affinity 
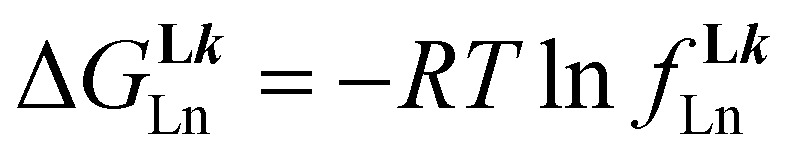
 modulated by an interligand interaction 
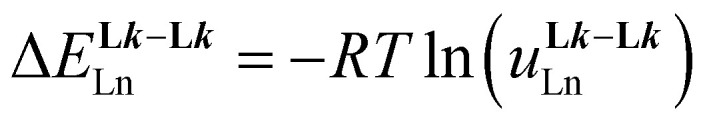
 fully characterizes the successive intermolecular ligand–metal binding association processes (Table 1, entries 6 and 7). The increase from 
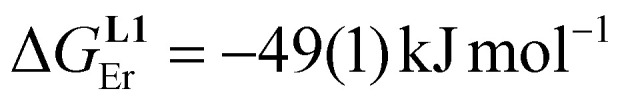
 to 
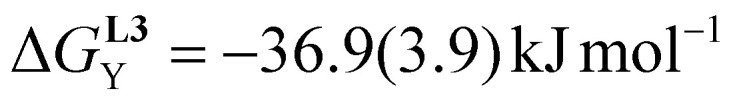
 quantifies a 25% reduction in affinity of Ln^3+^ for [L3]^+^, compared with that for L1, which is repeated each time a ligand is bound to the trivalent metallic center. Moreover, the concomitant 25% larger anti-cooperativity produced by repulsive interligand interactions estimated by 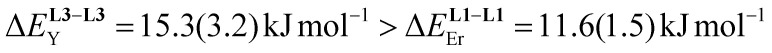 further penalizes the successive binding of [L3]^+^ to Ln^3+^ (Table 1, entry 7). The cationic character of the latter ligand, which is responsible for these drastic destabilizing effects, prevents the formation of the target saturated triple-helical [Ln(L3)_3_]^6+^ as a major component in acetonitrile solution at millimolar concentrations and prevents its exploitation as a potential multi-dye molecular-based upconverter.

### Combining intra- and intermolecular association in self-assembly reactions to provide stable [ZnLnZn(L5)_3_]^10+^ (Ln = Eu, Er, Y) helicates in solution

**Scheme 2:**
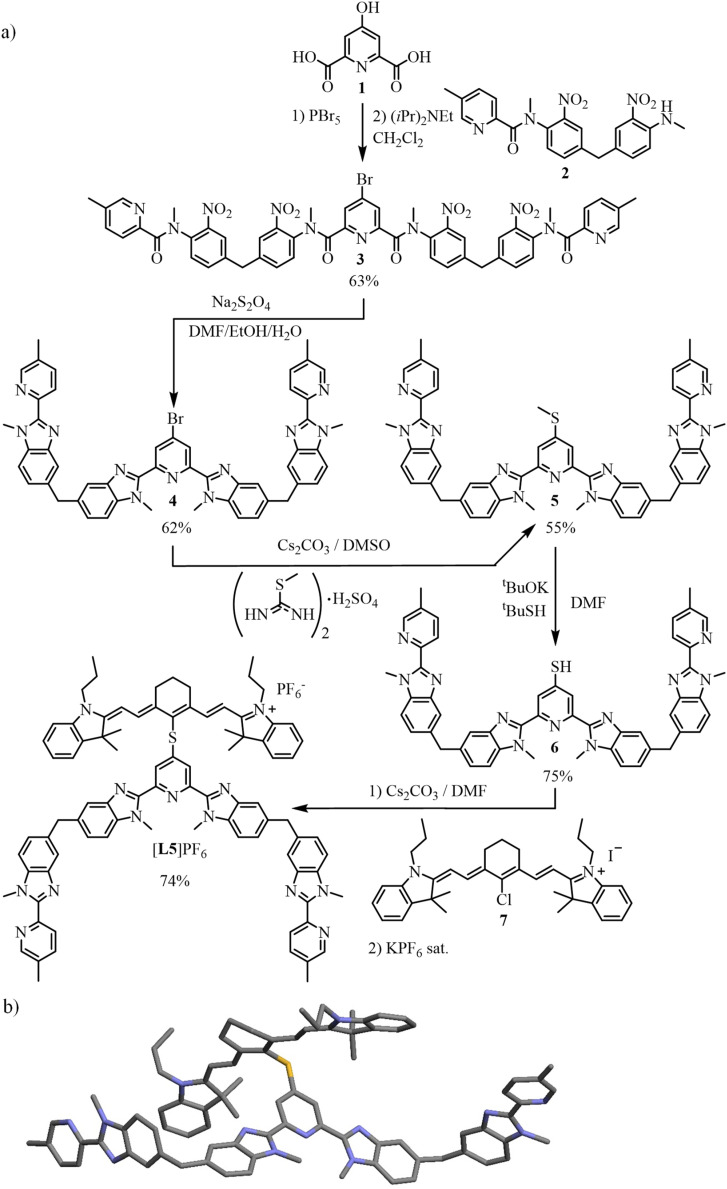
(a) Synthesis of the ligand [L5]PF_6_ and (b) molecular structure of [L5]^+^ in the crystal structure of [L5]PF_6_·C_3_H_5_N·0.25(C_5_H_12_O). Color codes: C = grey, N = dark blue, S = yellow.

The target segmental ligand [L5]^+^ combines the didentate–tridentate–didentate scaffold L4, which is programmed for the self-assembly of dimetallic trinuclear triple-stranded [ZnLnZn(L4)_3_]^7+^ helicates,^82,83^ with a cyanine [IR-780]I bound *via* a sulfur bridge at the 4-position of the central pyridine ring (Scheme 1). The multistep synthesis adapts previously published protocols^23,29,82,84^ to give 4, which was reacted with *S*-methylisothiourea^85^ and deprotected^86^ to yield thiol 6. Electrophilic attack with commercially available cyanine [IR-780]I (7) afforded the final ligand [L5]PF_6_ after metathesis (2% overall reaction yield for 13 steps; Scheme 2a, ESI Appendix 2†). Layering of *tert*-butyl methyl ether (C_5_H_12_O) on a propionitrile (C_3_H_5_N) solution of [L5]PF_6_ produced crystals of [L5]PF_6_·C_3_H_5_N·0.25(C_5_H_12_O) suitable for X-ray diffraction (Scheme 2b, Tables S1 and S2 and Fig. S7†).

**Fig. 3:**
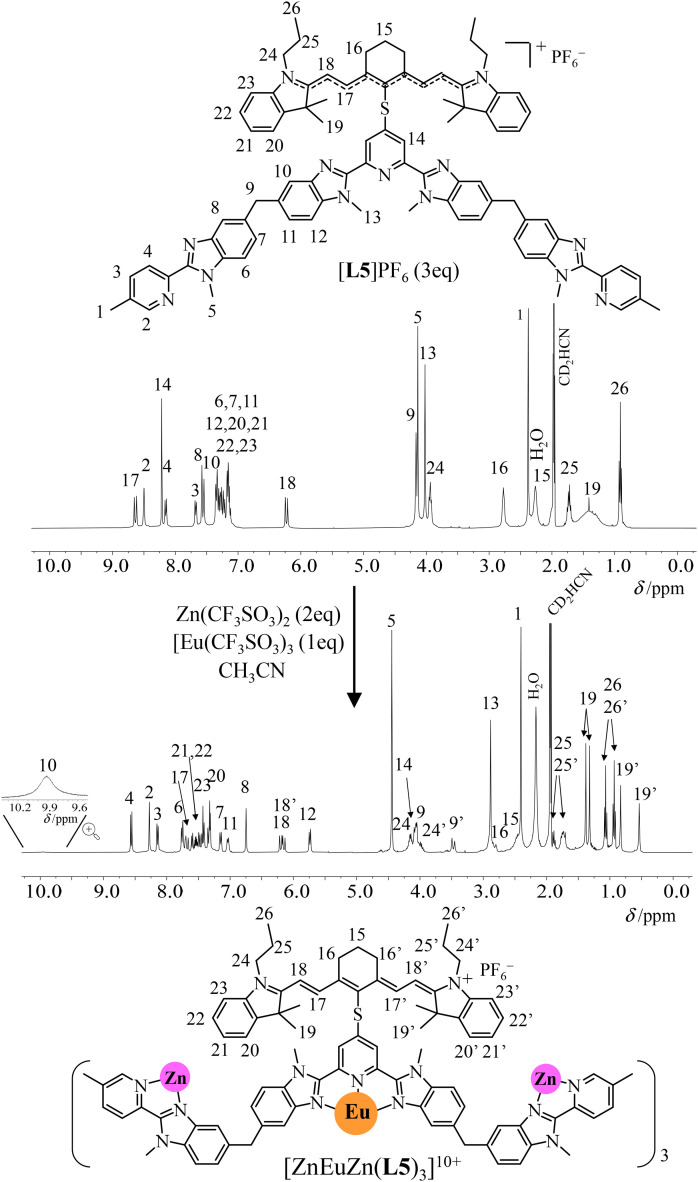
^1^H NMR spectra recorded for the self-assembly of [ZnEuZn(L5)_3_](PF_6_)_3_(CF_3_SO_3_)_7_ (CD_3_CN, 298 K).

In solution, the ^1^H NMR spectrum of [L5]^+^ display 26 signals in agreement with an average *C*_2v_ symmetry on the NMR time scale and the adoption of a symmetrical and delocalized form by the cyanine dye, a situation often referred to as the “*cyanine limit*” (Fig. 3, top).^87^ Notably, a significant signal broadening of the four H19 methyl groups of the cyanine backbone in [L5]^+^, which points to some hindered rotations around the C–S bonds due to the close distance between the cyanine and the segmental polyaromatic ligands. Among the possible d-block templating cations M^*z*+^ (M = Cr^2+^, Zn^2+^, Cr^3+^, Ga^3+^) compatible with the quantitative self-assembly of triple-helical [MLnM(L4)]^*n*+^ complexes,^26,82,83,88^ closed-shell diamagnetic Zn^2+^ appeared to be the best suited for (i) making detailed NMR analysis easier (paramagnetic Cr^2+^ and, especially, Cr^3+^ are not compatible with high-resolution spectra),^89^ (ii) allowing sufficiently fast kinetics for reaching thermodynamic equilibria within hours (Ga^3+^ requires days)^88^ and (iii) not affecting energy transfers between the dye and the emissive lanthanide (ETU mechanism). Consequently, the stepwise additions of two equivalents of Zn(CF_3_SO_3_)_2_ and one equivalent of Ln(CF_3_SO_3_)_3_ (Ln = Y, Fig. S8;† Ln = Eu, Fig. S9†) selectively and quantitatively provide the target self-assembled triple-stranded [ZnLnZn(L5)_3_]^10+^ helicates within a few hours at 50 °C (Fig. 3, bottom). The 15 signals observed for the 14 groups of protons attached to the ligand strands (numbered 1–14 in Fig. 3) points to a threefold symmetry. The loss of symmetry plane upon helication makes the protons H9 of the methylene bridges diastereotopic (H9 and H9′ in the final complex; Fig. 3 bottom and Fig. S8†) and confirms a global *D*_3_ point group for [ZnLnZn(L5)_3_]^10+^ as previously detailed for [ZnLnZn(L4)_3_]^7+^.^83^ The systematic doubling of the proton signals of the dye in the final [ZnLnZn(L5)_3_]^10+^ helices (for instance two different signals for H18–H18′ and four different signals for the diastereotopic methyl groups H19–H19′) corresponds to a local loss in symmetry, generally referred to as “*crossing the cyanine limit*”, induced by ion-pairing effects.^87,90^ The substantial increase of the total charge resulting from the complexation of [L5]^+^ to Zn^2+^ and Ln^3+^ is probably at the origin of the subsequent charge localization induced by ion pairing in the [ZnLnZn(L5)_3_]^10+^ helicate (Fig. 3, bottom).

In contrast to [Ln(L3)_3_]^6+^ (Ln = Eu, Y), which are quantitatively decomplexed at a total ligand concentration of 10^−4^ M (see previous section and Fig. A1-2 and A1-4†), the ^1^H NMR spectra of [ZnLnZn(L5)_3_]^10+^ recorded at total concentrations of 10^−4^–10^−5^ M show no change (Fig. S10 and S11†). This highlights the resistance to decomplexation boosted by the self-assembly process as the central ‘unstable’ [Ln(N^∩^N^∩^N)_3_]^6+^ unit is balanced by (i) the favorable formation of two stable [ZnN_6_]^2+^ scaffolds and (ii) the thermodynamic benefit^70,80^ of four preorganized intramolecular binding events to give the macrotetracyclic [ZnLnZn(L5)_3_]^10+^ helicate, which are lacking in the three successive anti-cooperative intermolecular binding processes leading to [Ln(L3)_3_]^6+^ (eqn (2)–(4)). Finally, whatever the order of addition of the metals to the solution of ligand [L5]^+^ is, the endpoint of the self-assembly remains invariant, which implies that all the possible kinetically accessible intermediates formed during the reaction will eventually fall into the thermodynamic minimum of the final helices (Fig. S12†). Evaporation of the solvent followed by (i) metathesis using an excess of KPF_6_ and (ii) size exclusion chromatography afforded [ZnYZn(L5)_3_](PF_6_)_10_·1.35H_2_O (yield 63%) and [ZnEuZn(L5)_3_](PF_6_)_10_·1.65H_2_O (yield 72%) (see Appendix 2 and Fig. S13†). The ESI-MS spectra display the expected series of multicharged adducts {[ZnLnZn(L5)_3_](PF_6_)_*n*_}^(10−*n*)+^ (*n* = 3–7; Fig. S14 and S15†), the isotopic distributions of which match the theoretical predictions (high-resolution mass spectroscopy time of flight, HR-MS ToF; Fig. S16 and S17†).

**Fig. 4:**
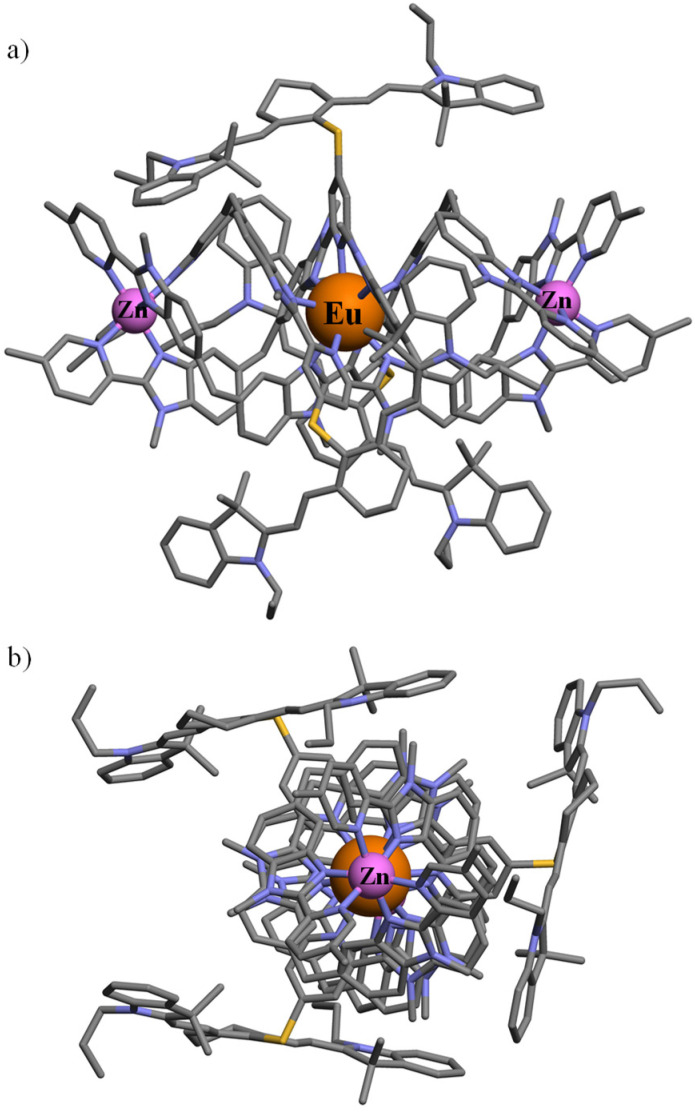
Proposed molecular structure of [ZnEuZn(L5)_3_]^10+^ built from the DFT-optimized gas-phase structure of [ZnEuZn(L4)_3_]^7+^ (ref. 83) and the molecular structure of [L3Er(hfac)_2_(CF_3_CO_2_)]^+^ (ref. 29) depicted (a) perpendicular to and (b) along the Zn⋯Eu⋯Zn axis (see text).

All attempts to obtain crystals suitable for X-ray diffraction studies failed in our hands, in a similar way to what was reported previously for the parent helicates [ZnEuZn(L4)_3_]X_7_ (X = ClO_4_^−^, CF_3_SO_3_^−^, PF_6_^−^).^83^ A conceivable molecular structure for [ZnEuZn(L5)_3_]^10+^ has been therefore built (Fig. 4) by combining the triple-helical platform reported for the DFT-optimized gas-phase structure of [ZnEuZn(L4)_3_]^7+^,^83^ which is isostructural with [CrEuCr(L4)_3_]^9+^ found in the X-ray crystal structure of [CrEuCr(L4)_3_](CF_3_SO_3_)_7_·(C_3_H_5_N)_30_ (CCDC 806425)^25^ or [GaErGa(L4)_3_]^9+^ found in the X-ray crystal structure of [GaErGa(L4)_3_](CF_3_SO_3_)_9_·(CH_3_CN)_35.5_ (CCDC 1003567),^27^ with the molecular structure of [L3Er(hfac)_2_(CF_3_CO_2_)]^+^ modelling the attached cyanine dyes (CCDC 2238348 in Fig. 1c).^29^

### Photophysical properties and ETU implemented in [ZnErZn(L5)_3_]^10+^ helicates in solution

**Fig. 5:**
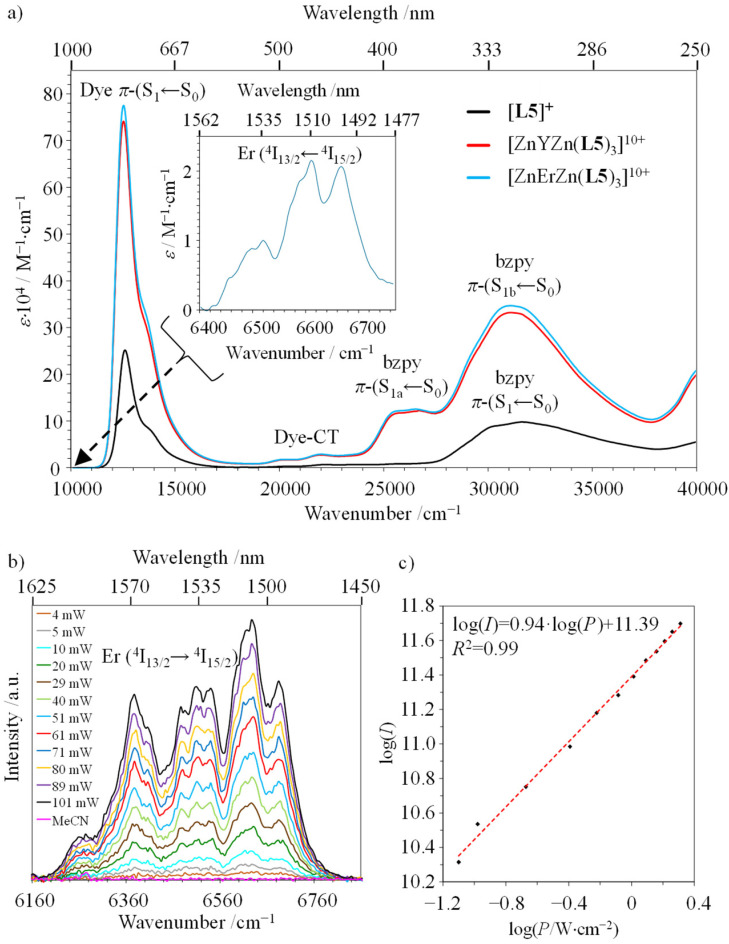
(a) Absorption spectra of ligand [L5]^+^ (3.1 × 10^−4^ M, black trace) and [ZnYZn(L5)_3_]^10+^ (1.4 × 10^−5^ M, red trace) and [ZnErZn(L5)_3_]^10+^ (1.7 × 10^−5^ M, blue trace) complexes recorded in acetonitrile solutions at 293 K. (b) Downshifted Er(^4^I_13/2_ → ^4^I_15/2_) emission of [ZnErZn(L5)_3_]^10+^ in acetonitrile solution (4 × 10^−5^ M, 293 K) upon laser excitation (*λ*_exc_ = 801 nm) for variable intensity powers and focused on a spot size of ∼0.06 cm^2^. (c) Associated log(*I*)–log(*P*) plot with *P* expressed in W cm^−2^.

The preparation of the erbium-containing triple-stranded [ZnErZn(L5)_3_]^10+^ helicate follows the same procedure used for its analogues [ZnLnZn(L5)_3_]^10+^ (Ln = Eu, Y; see above and Appendix 2†). It provides a diagnostic ^1^H NMR spectrum (Fig. S18†), in which the large paramagnetic moment and the slower electron relaxation rate associated with Er^3+^ ([Ar]4f^11^) result in broadened signals (Fig. S18†)^89^ and characteristic ESI-MS spectra (Fig. S19 and S20†). The absorption spectrum of the ligand [L5]^+^ is reminiscent of that of [L3]^+^, which combines the main NIR charge-transfer band of the cyanine dye around 780 nm (12 820 cm^−1^, Dye-π(S_1_ ← S_0_)) with several allowed intraligand π(S_*n*_ ← S_0_) transitions located on the polyaromatic backbone, and appearing in the UV part (300–250 nm; Fig. 5a).^29^ Upon complexation to the metallic cations to give [ZnLnZn(L5)_3_]^10+^ (Ln = Y, Er), the systematic *transoid*-to-*cisoid* rearrangements of the 2-benzimidazole-pyridine (bzpy) moieties are responsible for the reorganization of the polyaromatic scaffold, which results in a global splitting of the intraligand π(S_1_ ← S_0_) into two π(S_1a,b_ ← S_0_) bands in the UV-Vis domain (400–330 nm), while the dye-based NIR absorption is slightly broadened (Fig. 5a).^91^ Interestingly, the splitting of the π(S_1a,b_ ← S_0_) band can be exploited as a marker for the dissociation of the helical complexes in solution occurring at low concentrations. Its release indicates a stability limit in solution, which can be safely estimated at concentrations as low as 10^−5^ M in acetonitrile at 293 K (Fig. S21†). Moreover, the concomitant increase of the intensity of the Dye-π(S_1_ ← S_0_) transition upon dissociation of the complex (Fig. S21†) confirms the transformation of the localized polyene structure of the dye found in the highly charged [ZnLnZn(L5)_3_]^10+^ helicates (Fig. 3, bottom) into the delocalized *cyanine limit* in [L5]^+^ upon losing ion-pairing interactions (Fig. 3, top).^87,90^ The metal-centered Er(^2*S*+1^L_*J*_ ← ^4^I_15/2_) transitions are masked by intense ligand-based absorption covering the UV to NIR domain, and only the magnetic-allowed Er(^4^I_13/2_ ← ^4^I_15/2_) transition can be detected in the ‘free’ IR domain for [ZnErZn(L5)_3_]^10+^ (1510 nm; inset in Fig. 5a).

The emission spectra recorded upon ligand-centered excitation within the 280–320 nm range for both ligand [L5]^+^ and its complexes [ZnLnZn(L5)_3_]^10+^ (Ln = Y, Er) are dominated by the Stokes shifted NIR emission of the cyanine dye at 825–830 nm (Dye-π(S_1_ → S_0_)), together with some residual broad visible emission (400–450 nm) arising from the bound polyaromatic ligand strands π(S_1_ → S_0_) (Fig. S22, S24 and S26†). The associated excitation spectra (*λ*_em_ = 825 nm; Fig. S23, S25 and S27†) confirm the efficient communication between the appended cyanine dye and the polyaromatic scaffold, while the erbium-based IR Er(^4^I_13/2_ → ^4^I_15/2_) emissions at 1520 nm, induced upon either UV excitation (*λ*_exc_ = 325 nm; Fig. S28a†) or cyanine-based excitation (*λ*_exc_ = 805 nm; Fig. 5b and Fig. S28b†), provide the proof for the ultimate energy funneling toward the erbium emissive center and the operation of a linear one-photon downshifted emission mechanism (Fig. 5c, slope = 0.94).^67,92^

**Fig. 6:**
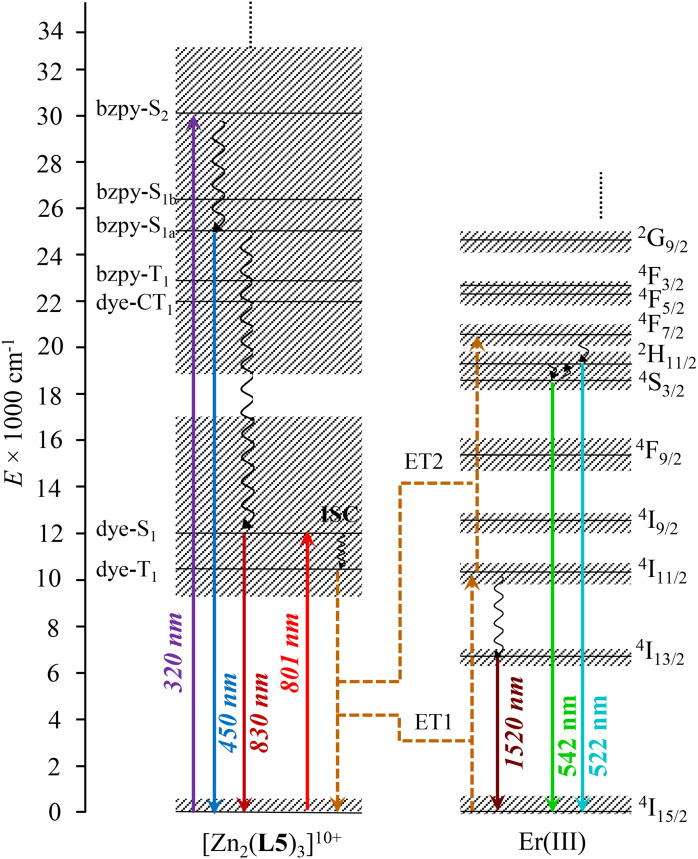
Jablonski diagram established for [ZnErZn(L5)_3_]^10+^, illustrating the mechanisms for inducing light downshifting and light UC through ligand-sensitized ETU. ET = intramolecular S → A energy transfer; ISC = intersystem crossing. The dye-centered triplet states are located according to ref. 62.

The associated Jablonski diagram established for [ZnErZn(L5)_3_]^10+^ (Fig. 6) appears to be similar to that previously found for [Er(L3)(hfac)_3_]^+^.^29^ Because of the thermodynamic requirement of using ≥10^−5^ M solution of complexes for avoiding any detectable dissociation, the emission spectra upon NIR dye-centered excitation of [ZnErZn(L5)_3_]^10+^ at 801 nm (*ε* = 778 900 M^−1^ cm^−1^, Fig. 5a) are recorded using the front face technique with a 1 mm cuvette to prevent primary and secondary inner filter effects due to the considerable absorptivity of the complexes.^93^

NIR time-gated phosphorescence upon ligand-centered excitation of [ZnLnZn(L5)_3_]^10+^ (Ln = Y, Er) within the 280–320 nm range was attempted at low temperature (77 K) but no emitted signal could be detected. This demonstrates that no excited triplet state from either the polyaromatic scaffold or the cyanine dye in these complexes induces phosphorescence. They probably relax non-radiatively (*via* vibrational quenching and/or energy transfer) as demonstrated by Garfield *et al*. for related cyanine dyes.^62^ Focusing on the dye, it is worth recalling here that ultrafast lifetimes recorded previously for the residual singlet Dye-π(S_1_ → S_0_) emission detected at 825–830 nm for [L3]^+^ and [L3Ln(hfac)_3_]^+^ (Ln = Er, Y) in solution established unambiguously that the Dye-π(T_1_) state indeed plays a pivotal role for feeding the Er(^4^I_11/2_) in [L3Er(hfac)_3_]^+^*via* intramolecular energy transfer (ET1 in Fig. 5).^38^

**Fig. 7:**
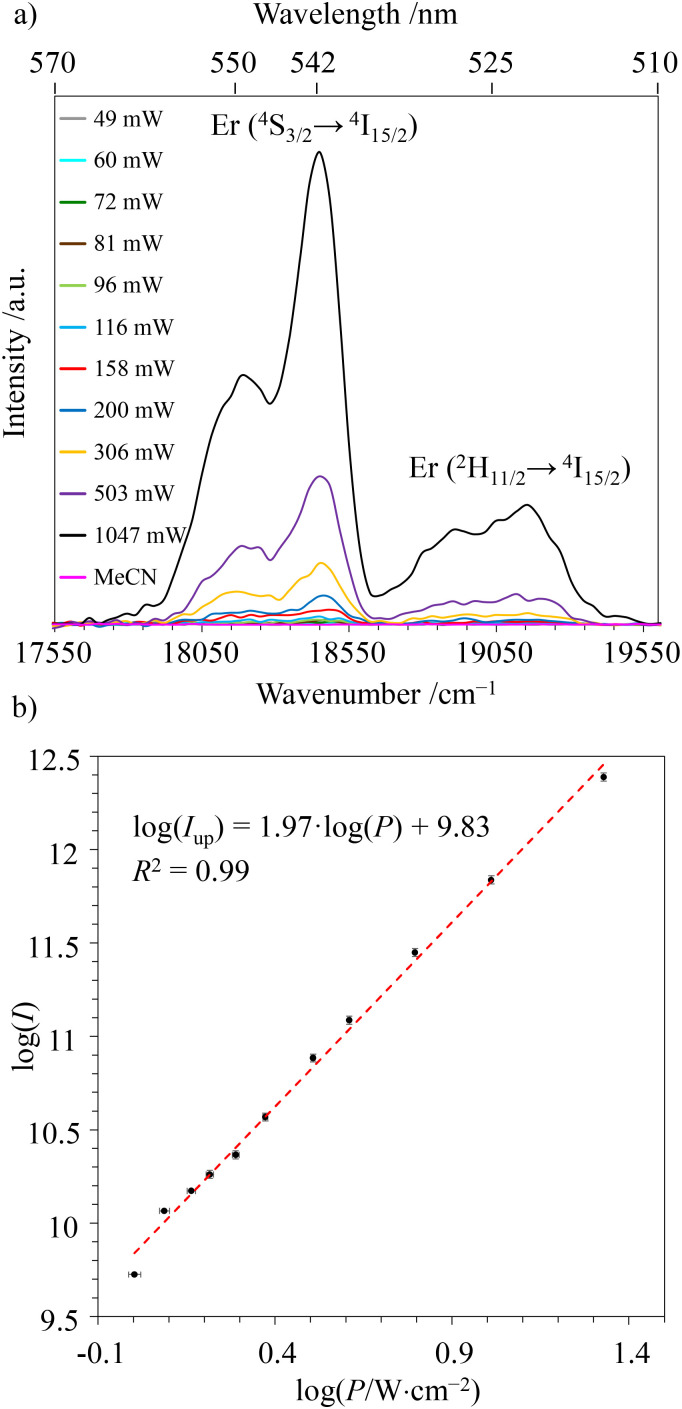
(a) Upconverted Er(^4^S_3/2_ → ^4^I_15/2_) and Er(^2^H_11/2_ → ^4^I_15/2_) signals of [ZnErZn(L5)_3_]^10+^ in acetonitrile solution (4 × 10^−5^ M, 293 K) upon continuous wave laser excitation (*λ*_exc_ = 801 nm) for variable intensity power and focused on a spot size of ∼0.06 cm^2^. The blank corresponds to pure acetonitrile excited at 801 nm with *P* = 16.7 W cm^−2^. (b) Associated log(*I*_up_)–log(*P*) plot with *P* expressed in W cm^−2^.

Switching now to NIR laser excitation at *λ*_exc_ = 801 nm of [ZnErZn(L5)_3_]^10+^ (10^−5^ M concentration), this produces a negligible metal-centered Er(^4^I_9/2_ ← ^4^I_15/2_) absorbance *A* = log(*I*_0_/*I*) = 2 × 10^−7^ (*ε*_Er_ ≈ 0.2 M^−1^ cm^−1^, 1 mm cell),^15^ which prevents the operation of detectable competitive ESA mechanism in these conditions. On the contrary, the latter NIR laser excitation beam at *λ*_exc_ = 801 nm is strongly absorbed by the dyes of [ZnErZn(L5)_3_]^10+^ at 10^−5^ M in acetonitrile at room temperature (*A* = log(*I*_0_/*I*) = 0.78 using *ε* = 78 × 10^4^ M^−1^ cm^−1^ for the Dye-π(S_1_ ← S_0_) transition, 1 mm cell). This results in the detection of not only the standard downshifted Er(^4^I_13/2_ → ^4^I_15/2_) transition at 1520 nm (Fig. 5b) but also two well-resolved green upconverted Er(^4^S_3/2_ → ^4^I_15/2_) (542 nm) and Er(^2^H_11/2_ → ^4^I_15/2_) (525 nm) emission bands (Fig. 7a and S29†) following the ETU mechanism (ET1 and ET2 in Fig. 6). The log–log treatment of the UC signal gives a slope of 1.97 which confirms the piling up of two successive photons (Fig. 7b). The UC quantum yield (*ϕ*^UC^) of [ZnErZn(L5)_3_]^10+^ in acetonitrile was determined using the relative method with parent [L3Er(hfac)_3_]^+^ as reference (Table 2; see Appendix 2† for details).^29^

**Table 2:** Upconverted quantum yield *ϕ*^UC^ and brightness *B*^UC^ recorded for molecular complexes in acetonitrile at room temperature at *P* = 25 W cm^−2^

Compound	*ε*_max_/M^−1^ cm^−1^	*ϕ* ^UC^	*B* ^UC^/M^−1^ cm^−1^	Ref.
[L2Er(hfac)_3_]^+^	237 000	3.4(4) × 10^−9^	8.0(8) × 10^−4^	28
[L3Er(hfac)_3_]^+^	194 200	1.1(2) × 10^−8^	2.0(1) × 10^−3^	29
[ZnErZn(L5)_3_]^10+^	778 900	3.6(4) × 10^−8^	2.8(3) × 10^−2^	This work

Compared with those for the mono-dye model complex [L3Er(hfac)_3_]^+^, *ϕ*^UC^ increases by a factor of 3.3(7) and *B*^UC^ by a factor of 14(2) for [ZnErZn(L5)_3_]^10+^. These boosting components align well with the predictions of a threefold increase in the quantum yield and a ninefold increase in brightness according to the ETU mechanism highlighted by a green pathway in Fig. 2. Any additional contribution originating from the concerted-ETU mechanism (red pathway in Fig. 2) appears to be small, if not negligible, as previously established for the [CrErCr(L4)_3_]^9+^ analogue.^23^ Theoretical modelling of the concerted-ETU mechanism^26^ applied to [S_*n*_A] chromophores highlighted the delicate balance between a favorable long excited-state lifetime located on the sensitizer (S) for accumulating the incident photons and fast sensitizer-to-activator (S → A) energy transfers, which contribute to reduce the sensitizer-based excited-state lifetime, for ultimately optimizing quantum yields. In the absence of accessible, reliable, and detailed kinetic analysis of the UC mechanism in [ZnErZn(L5)_3_]^10+^, no definitive rationalization is at hand, but the short lifetime of the feeding level of [L3]^+^, previously measured and reported for [L3Er(hfac)_3_]^+^,^29^ strongly suggests that a similar scenario operates for [L5]^+^ dye triplet state in [ZnErZn(L5)_3_]^10+^. This prevents sufficient accumulation of photons on the sensitizers prior to successive intramolecular energy transfers onto the activator.

## Conclusion

The connection of a cationic cyanine dye to a tridentate 2,6-bis(benzimidazol-2-yl)pyridine ligand in [L3]^+^ limits the intermolecular affinity for binding Ln^3+^ to such an extent that the target triple-helical [Ln(L3)_3_]^6+^ complex is not accessible, and a maximum of two guests can be connected to give 80% of [Ln(L3)_2_]^5+^ at millimolar concentrations in acetonitrile. Taking advantage of self-assembly processes overcomes this thermodynamic restriction, and three cationic dyes can be successfully attached to a central trivalent lanthanide in triple-stranded [ZnErZn(L5)_3_]^10+^ helicate at 10^−5^ M in acetonitrile solution at room temperature. NIR laser excitation (*λ*_exc_ = 801 nm) of the Dye-π(S_1_ ← S_0_) transition in [ZnErZn(L5)_3_]^10+^ results in the detection of both downshifted Er(^4^I_13/2_ → ^4^I_15/2_) emission at 1520 nm (one-photon process) and green upconverted Er(^4^S_3/2_ → ^4^I_15/2_) (542 nm) and Er(^2^H_11/2_ → ^4^I_15/2_) (525 nm) emission bands (two-photon processes). As expected by ETU modeling (green pathway in Fig. 2), the UC quantum yield is boosted by a factor of ≈3 going from [L3Er(hfac)_3_]^+^ (one sensitizer)^29^ to [ZnErZn(L5)_3_]^10+^ (three sensitizers). The brightness of [ZnErZn(L5)_3_]^10+^, *B*^UC^ = 2.8(3) × 10^−2^ M^−1^ cm^−1^, benefits a second time from a boosting factor of ≈three due to the presence of three dye sensitizers attached to the erbium emitter, which makes it finally one order of magnitude larger than the previous record held by [L3Er(hfac)_3_]^+^ (*B*^UC^ = 2.0(1) × 10^−3^ M^−1^ cm^−1^, Table 2).

## Conflicts of interest

The authors declare no conflict of interest.

## Supplementary Material

DT-054-D5DT00438A-s001

DT-054-D5DT00438A-s002

## Data Availability

The data that support the findings of this study are available from the corresponding authors upon reasonable request.
