# Supplementary material for: Self-assembled multidye-sensitized erbium single molecules for boosting energy transfer light upconversion in solution
Source: Dalton Trans. 2025 May 29;54(25):9937–48. doi: 10.1039/d5dt00438a (PMC12150277; doi:10.1039/d5dt00438a)
Supplement: DT-054-D5DT00438A-s001 [file DT-054-D5DT00438A-s001.pdf]

Supplementary Information (SI) for Dalton Transactions.
This journal is © The Royal Society of Chemistry 2025

Self-assembled Multidyes-Sensitized Erbium Single Molecules for Boosting Energy Transfer Light-Upconversion in Solution.

*Filipe Alves, Inès Taarit, Laure Guénée, Claude Piguet**

Supporting Information

(54 pages)

Appendix 1. ^{1}H NMR titrations and site-binding model applied to equilibria (2)-(4).

Titrations of [**L3**]$^+$ (0.5 mM) by stepwise additions of $Eu(CF_3SO_3)_3$ in CD_3CN at 298 K (Fig. A1-1) confirmed the formation of [Eu(**L3**)]$^{4+}$ and [Eu(**L3**)$_2$]$^{5+}$, characterized by well-resolved sets of specific ^{1}H NMR signals, together with the formation of traces of [Eu(**L3**)$_3$]$^{6+}$. The existence of the latter complex is suggested by weak broadened signals (blue markers in Fig. A1-1) which are diagnostic for the operation of a [Eu(**L3**)$_2$]$^{5+}$ +[**L3**]$^+$ $\rightleftharpoons$ [Eu(**L3**)$_3$]$^{6+}$ exchange process with intermediate kinetic rate on the NMR time scale.

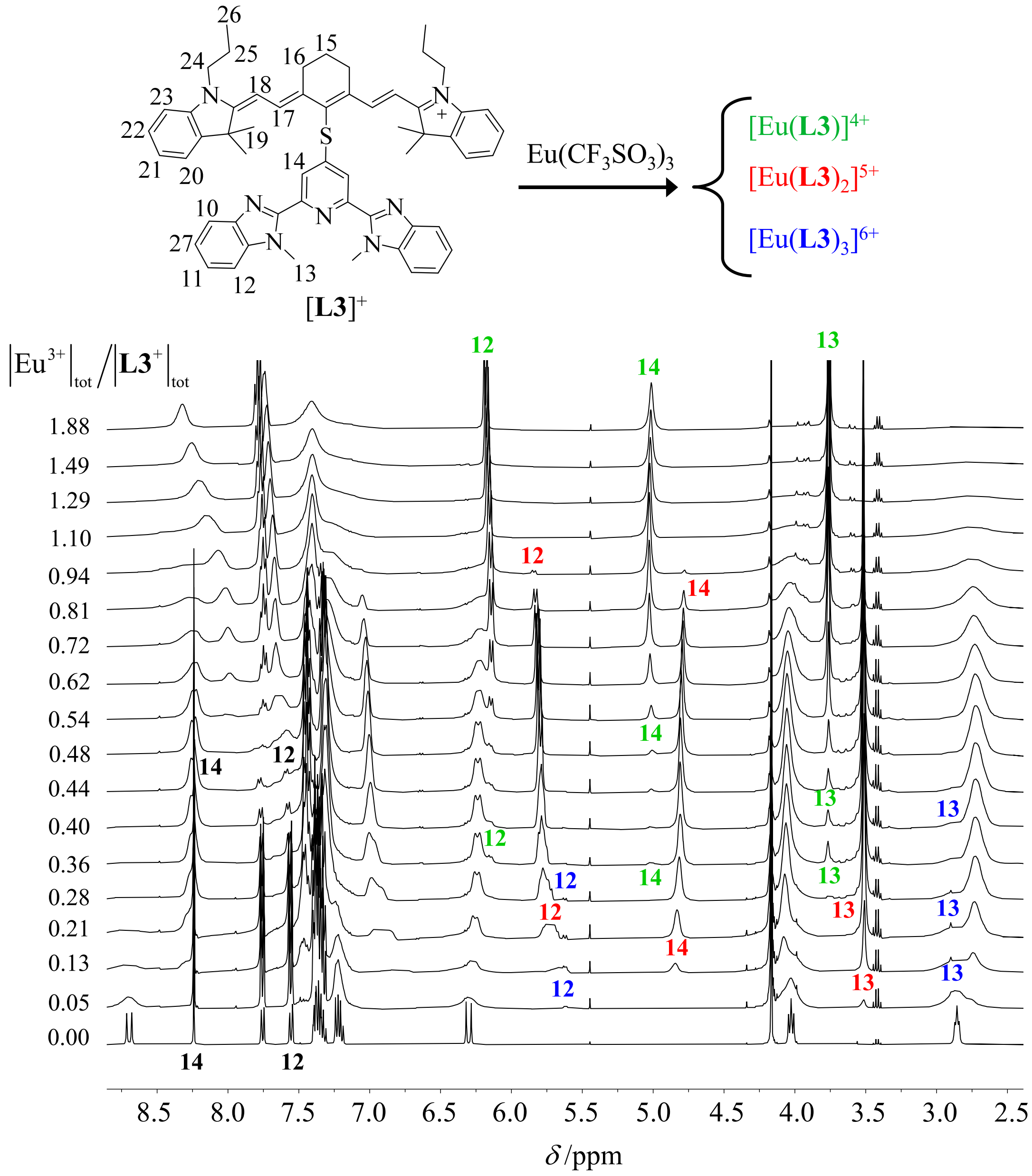

Fig. A1-1 ^{1}H NMR (400 MHz) spectra of the titration of [**L3**]$^+$ with $Eu(CF_3SO_3)_3$ in CD_3CN at 298 K with partial numbering scheme used for integration ($8.39\cdot10^{-3} \leq |\mathbf{L3}|_{tot} \leq 5.21\cdot10^{-3}$ M and $4.25\cdot10^{-4} \leq |Eu|_{tot} \leq 9.79\cdot10^{-3}$ M).

It was possible to estimate the concentrations of each species by simple integrations of the various ^{1}H NMR signals (Fig. A1-2) so that the experimental occupancy factor of the (host) metal $\theta_{\mathrm{Eu}}^{\mathrm{exp}}$ and the free concentration of (guest) ligand $|\mathbf{L3}| = |\mathbf{L3}|_{\mathrm{tot}} - 3\theta_{\mathrm{Ln}}^{\mathrm{exp}}|\mathrm{Ln}|_{\mathrm{tot}}$ become available (eq A1-1).

$$\theta_{\mathrm{Ln}}^{\mathrm{exp}} = \frac{1}{3}\frac{|\mathbf{L3}|_{\mathrm{bound}}}{|\mathrm{Ln}|_{\mathrm{tot}}} = \frac{1}{3}\frac{\left(I_{\mathrm{Ln}\mathbf{L3}} + I_{\mathrm{Ln}(\mathbf{L3})_2} + I_{\mathrm{Ln}(\mathbf{L3})_3}\right)|\mathbf{L3}|_{\mathrm{tot}}}{\left(I_{\mathbf{L3}} + I_{\mathrm{Ln}\mathbf{L3}} + I_{\mathrm{Ln}(\mathbf{L3})_2} + I_{\mathrm{Ln}(\mathbf{L3})_3}\right)|\mathrm{Ln}|_{\mathrm{tot}}} = \frac{1}{3}\frac{|\mathbf{L3}|_{\mathrm{tot}} - |\mathbf{L3}|}{|\mathrm{Ln}|_{\mathrm{tot}}} \qquad \text{(A1-1)}$$

Non-linear least-squares fits of $\theta_{\mathrm{Eu}}^{\mathrm{exp}}$ with respect to the theoretical binding isotherms $\theta_{\mathrm{Eu}}^{\mathrm{cald}}$ (eq A1-2) derived from equilibria (2)–(4) provide rough stability constants $\beta_{1,n}^{\mathrm{Ln},\mathbf{L3}}$ (n = 1–3, Fig. A1-2) gathered in Table 1 (entries 2–3).

$$\theta_{\mathrm{Ln}}^{\mathrm{cald}} = \frac{1}{3}\frac{\sum_{n=1}^{3} n\beta_{1,n}^{\mathrm{Ln},\mathbf{L3}}\left(|\mathbf{L3}|\right)^n}{1+\sum_{n=1}^{3}\beta_{1,n}^{\mathrm{Ln},\mathbf{L3}}\left(|\mathbf{L3}|\right)^n} \qquad \text{(A1-2)}$$

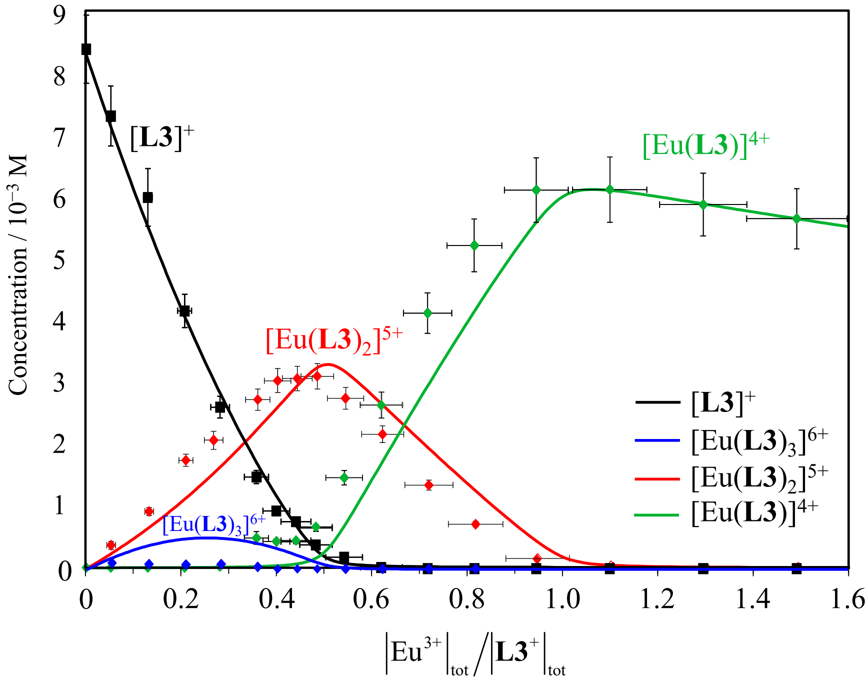

Fig. A1-2. Speciation deduced by integration of ^{1}H NMR signals collected during the titration of $[\mathbf{L3}]^+$ with $Eu(CF_3SO_3)_3$ in CD_3CN at 298 K. The full traces correspond to the concentrations computed[A1-1] by using the fitted stability constants $\beta_{1,n}^{\mathrm{Eu},\mathbf{L3}}$ gathered in Table 1.

Repeating the titration of $[\mathbf{L3}]^+$ in the same conditions, but using diamagnetic and more compact $Y(CF_3SO_3)_3$ gave related results (Figs A1-3and A1-4) , but with weaker cumulative stability constants (Table 1, entry 3) in agreement with the operation of an anti-electrostatic trend along the lanthanide series previously established for the successive binding of **L1** around trivalent lanthanides.[79,80]

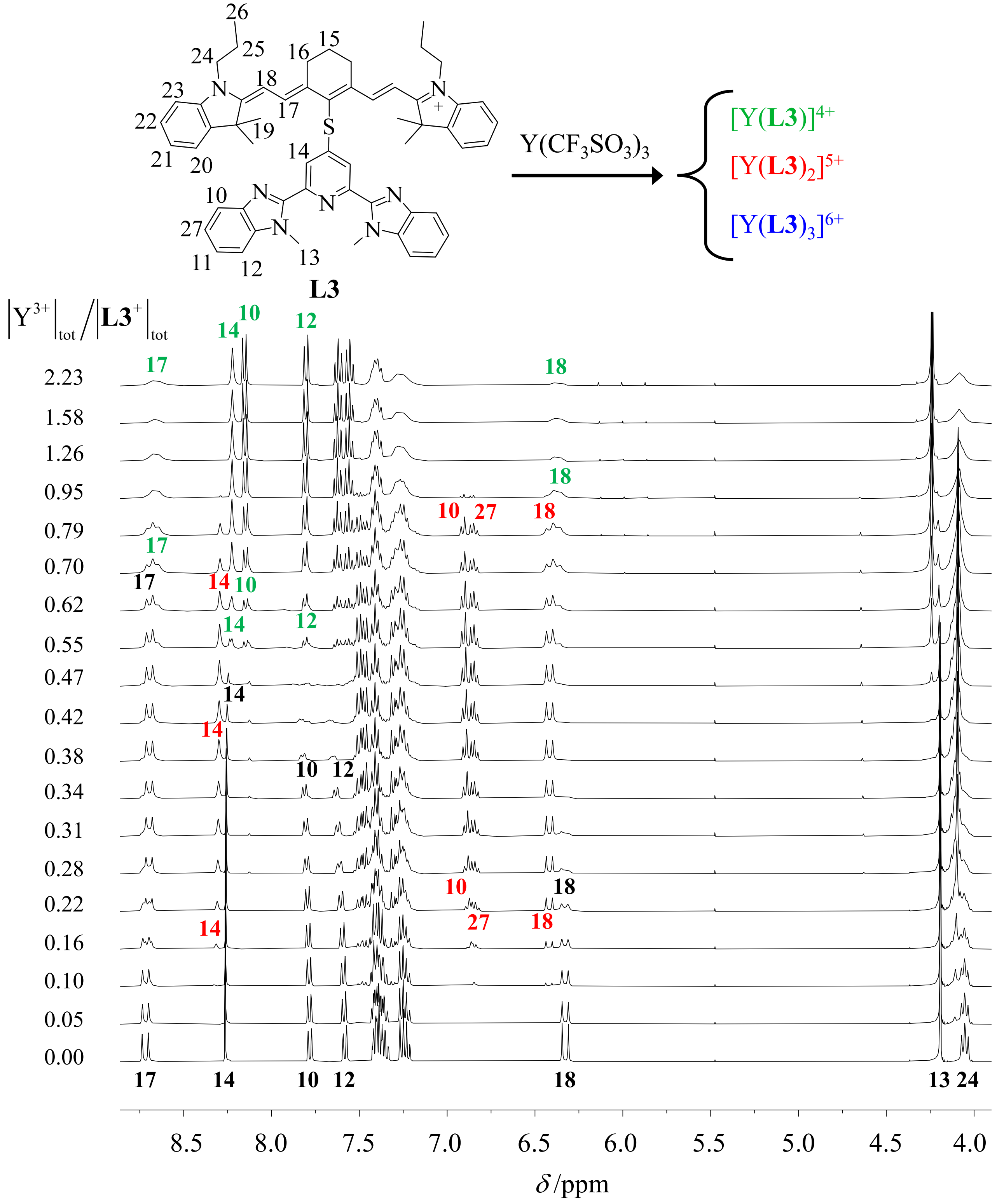

Fig. A1-3. ^{1}H NMR (400 MHz) spectra of the titration of [**L3**]$^+$ with $Y(CF_3SO_3)_3$ in CD_3CN at 298 K with partial numbering scheme used for integration ($8.52\cdot10^{-3} \leq |\mathbf{L3}|_{tot} \leq 4.93\cdot10^{-3}$ M and $4.03\cdot10^{-4} \leq |Y|_{tot} \leq 1.10\cdot10^{-2}$ M).

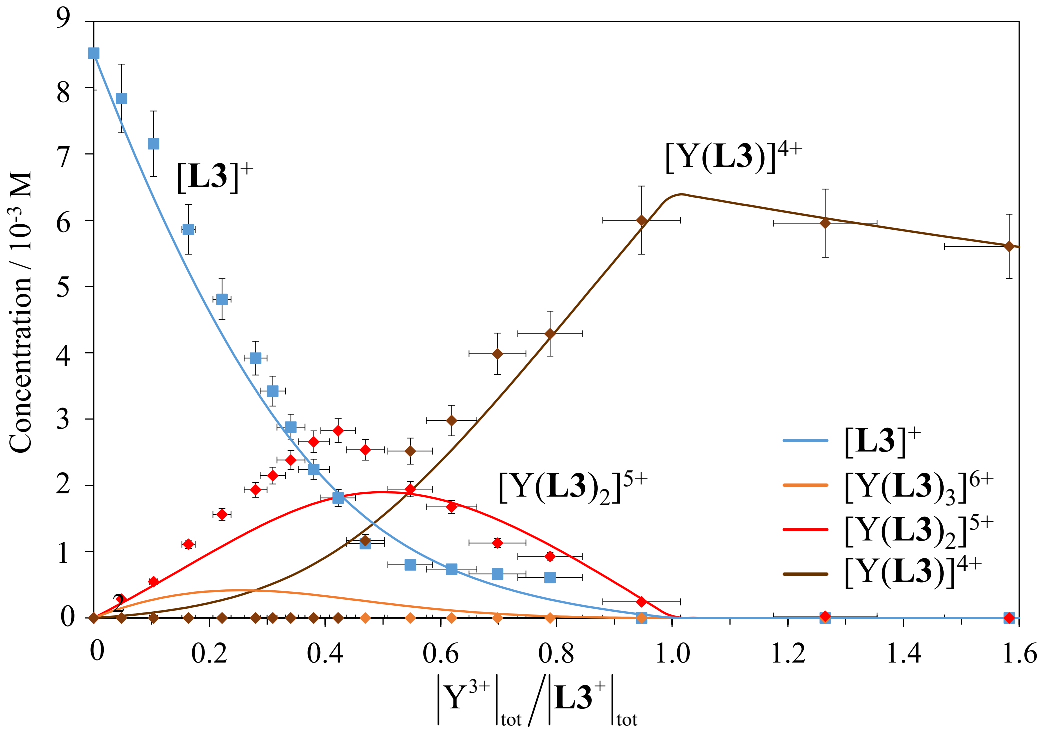

Fig. A1-4. Speciation deduced by integration of ^{1}H NMR signals collected during the titration of [**L3**]$^{+}$ with $Y(CF_3SO_3)_3$ in CD_3CN at 298 K. The full traces correspond to the concentrations computed[A1-1] by using the fitted stability constants $\beta_{1,n}^{\mathrm{Y,L3}}$ gathered in Table 1.

The site binding model considers ligands [**L3**]$^{+}$ as guests, for which one can extract their intrinsic intermolecular affinity toward the metal host $\Delta G_{\mathrm{Ln}}^{\mathbf{L}k} = -RT \ln f_{\mathrm{Ln}}^{\mathbf{L}k}$, together with the interligand interactions $\Delta E_{\mathrm{Ln}}^{\mathbf{L}k\text{-}\mathbf{L}k} = -RT \ln\left(u_{\mathrm{Ln}}^{\mathbf{L}k\text{-}\mathbf{L}k}\right)$ which are related to the standard cooperativity factor $u_{\mathrm{Ln}}^{\mathbf{L3\text{-}L3}}$.[A1-2 to A1-4] In fact, any formation constant β can be decomposed into two constants in eq. (A1-3),[A1-5] where K_{stat} takes into account the purely statistical contribution produced by the change in rotational entropies [A1-6] and K_{chem} measures (i) the chemical affinity between the host and the guests $f_{\mathrm{Ln}}^{\mathbf{L3}}$ and (ii) the cooperative $u_{\mathrm{Ln}}^{\mathbf{L3\text{-}L3}}$ (or anti-cooperative) effect produced by the interactions between multiple neighboring guests on the same host.[A1-4,A1-7]

$$\beta = K_{\mathrm{stat}} \cdot K_{\mathrm{chem}} \qquad \text{(A1-3)}$$

The statistical contributions computed by using the symmetry number technique are gathered in Fig. A1-5, which leads to eqs (A1-4) to (A1-6) for modeling equilibria (2)-(4).

$$\beta_{1,1}^{\mathrm{Ln},\mathbf{L3}} = 6 \cdot f_{\mathrm{Ln}}^{\mathbf{L3}} \qquad \text{(A1-4)}$$

$$\beta_{1,2}^{\mathrm{Ln},\mathbf{L3}} = 12 \cdot \left(f_{\mathrm{Ln}}^{\mathbf{L3}}\right)^2 \cdot u_{\mathrm{Ln}}^{\mathbf{L3\text{-}L3}} \qquad \text{(A1-5)}$$

$$\beta_{1,3}^{\mathrm{Ln},\mathbf{L3}} = 16 \cdot \left(f_{\mathrm{Ln}}^{\mathbf{L3}}\right)^3 \cdot \left(u_{\mathrm{Ln}}^{\mathbf{L3\text{-}L3}}\right)^3 \qquad \text{(A1-6)}$$

Multi-linear least-squares techniques (eq A1-7 and A1-8) are used to extract $f_{\mathrm{Ln}}^{\mathbf{L3}}$ and $u_{\mathrm{Ln}}^{\mathbf{L3\text{-}L3}}$ from which $\Delta G_{\mathrm{Ln}}^{\mathbf{L3}} = -RT\ln f_{\mathrm{Ln}}^{\mathbf{L3}}$, together with the interligand interactions $\Delta E_{\mathrm{Ln}}^{\mathbf{L3\text{-}L3}} = -RT\ln\left(u_{\mathrm{Ln}}^{\mathbf{L3\text{-}L3}}\right)$ can be estimated and collected in Table 1.

a)

$[Ln(CH_3CN)_9]^{3+}$ + 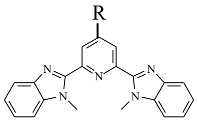 $\underset{}{\overset{\beta_{1,1}^{\mathrm{Ln,L3}}}{\rightleftarrows}}$ $[Ln(\mathbf{L3})(CH_3CN)_6]^{4+}$ + 3 CH_3CN

$[\mathbf{L3}]^{+}$

Point group	D_{3h}	C_{2v}	C_{2v}	C_{3v}
σ_{ext}	6	2	2	3
σ_{int}	3^9	3^2	$3^2\times3^6$	1
σ_{mix}	1	1	1	1

$$\omega_{1,1}^{\mathrm{chiral}} = \frac{1\times1}{1\times(1)^3} = 1,\ \omega_{1,1}^{\mathrm{Ln},\mathbf{L3}} = \frac{6\times3^9\times2\times3^2}{2\times3^2\times3^6\times(3)^3} = 6 \Rightarrow K_{1,1}^{\mathrm{stat}} = \omega_{1,1}^{\mathrm{chiral}}\cdot\omega_{1,1}^{\mathrm{Ln},\mathbf{L3}} = 6$$

b)

$[Ln(CH_3CN)_9]^{3+}$ + 2 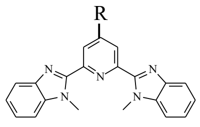 $\overset{\beta_{1,2}^{\mathrm{Ln,L3}}}{\rightleftarrows}$ $[Ln(\mathbf{L3})_2(CH_3CN)_3]^{5+}$ + 6 CH_3CN

$[\mathbf{L3}]^{+}$

Point group	D_{3h}	C_{2v}	C_{2v}	C_{3v}
σ_{ext}	6	2	2	3
σ_{int}	3^9	3^2	$3^4\times3^3$	1
σ_{mix}	1	1	1	1

$$\omega_{1,2}^{\mathrm{chiral}} = \frac{1\times1^2}{1\times(1)^6} = 1,\ \omega_{1,2}^{\mathrm{Ln},\mathbf{L3}} = \frac{6\times3^9\times(2)^2\times(3^2)^2}{2\times3^4\times3^3\times(3)^6} = 12 \Rightarrow K_{1,2}^{\mathrm{stat}} = \omega_{1,2}^{\mathrm{chiral}}\cdot\omega_{1,2}^{\mathrm{Ln},\mathbf{L3}} = 12$$

c)

$[Ln(CH_3CN)_9]^{3+}$ + 3 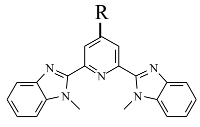 $\overset{\beta_{1,3}^{\mathrm{Ln,L3}}}{\rightleftarrows}$ $[Ln(\mathbf{L2})_3]^{6+}$ + 9 CH_3CN

$[\mathbf{L3}]^{+}$

Point group	D_{3h}	C_{2v}	D_3	C_{3v}
σ_{ext}	6	2	6	3
σ_{int}	3^9	3^2	3^6	1
σ_{mix}	1	1	$\frac{1}{2}$	1

$$\omega_{1,3}^{\mathrm{chiral}} = \frac{1\times1^3}{\frac{1}{2}\times(1)^9} = 2,\ \omega_{1,3}^{\mathrm{Ln},\mathbf{L3}} = \frac{6\times3^9\times(2)^3\times(3^2)^3}{6\times3^6\times(3)^9} = 8 \Rightarrow K_{1,3}^{\mathrm{stat}} = \omega_{1,3}^{\mathrm{chiral}}\cdot\omega_{1,3}^{\mathrm{Ln},\mathbf{L3}} = 16$$

Fig. A1-5 Symmetry numbers obtained by *Benson*'s method and associated statistical factors computed for the formation of a) $[Ln(\mathbf{L3})]^{4+}$, b) $[Ln(\mathbf{L3})_2]^{5+}$ and c) $[Ln(\mathbf{L3})_3]^{6+}$.

$$\log\left(\beta_{1,1}^{\mathrm{Ln},\mathbf{L3}}\right)=\log(6)+\log\left(f_{\mathrm{Ln}}^{\mathbf{L3}}\right)$$
$$\log\left(\beta_{1,2}^{\mathrm{Ln},\mathbf{L3}}\right)=\log(12)+2\log\left(f_{\mathrm{Ln}}^{\mathbf{L3}}\right)+\log\left(u_{\mathrm{Ln}}^{\mathbf{L3}\text{-}\mathbf{L3}}\right) \quad \text{(A1-7)}$$
$$\log\left(\beta_{1,3}^{\mathrm{Ln},\mathbf{L3}}\right)=\log(16)+3\log\left(f_{\mathrm{Ln}}^{\mathbf{L3}}\right)+3\log\left(u_{\mathrm{Ln}}^{\mathbf{L3}\text{-}\mathbf{L3}}\right)$$

$$\begin{pmatrix}\log\left(\beta_{1,1}^{\mathrm{Ln},\mathbf{L3}}\right)\\ \log\left(\beta_{1,2}^{\mathrm{Ln},\mathbf{L3}}\right)\\ \log\left(\beta_{1,3}^{\mathrm{Ln},\mathbf{L3}}\right)\end{pmatrix}-\begin{pmatrix}\log(6)\\ \log(12)\\ \log(16)\end{pmatrix}=\log\left(f_{\mathrm{Ln}}^{\mathbf{L3}}\right)\begin{pmatrix}1\\2\\3\end{pmatrix}+\log\left(u_{\mathrm{Ln}}^{\mathbf{L3}\text{-}\mathbf{L3}}\right)\begin{pmatrix}0\\1\\3\end{pmatrix} \quad \text{(A1-8)}$$

References

A1-1 L. Alderighi, P. Gans, A. Ienco, D. Peters, A. Sabatini and A. Vacca, *Coord. Chem. Rev.,* 1999, **184**, 311–318.

A1-2 G. Koper and M. Borkovec, *J. Phys. Chem. B,* 2001, **105**, 6666-6674.

A1-3 J. Hamacek, M. Borkovec and C. Piguet, *Dalton Trans.,* 2006, 1473-1490.

A1-4 C. Piguet, *Chem. Commun.*, 2010, **46**, 6209-6231.

A1-5 S. W. Benson, *J. Am. Chem. Soc.*, 1958, **80**, 5151-5154.

A1-6 G. Ercolani, C. Piguet, M. Borkovec, J. Hamacek, *J. Phys. Chem. B*, 2007, **111**, 12195-12203.

A1-7 B. Golesorkhi, L. Guénée, H. Nozary, A. Fürstenberg, Y. Suffren, S. V. Eliseeva, S. Petoud, A. Hauser and C. Piguet, *Chem. Eur. J.*, 2018, **24**, 13158-13169.

Appendix 2. Experimental section

All chemicals were purchased from commercial sources and used without further purification if not stated otherwise. Silica-gel plates (Merck, 60 F254) and aluminum oxide plates (ALUGRAM® ALOX N / UV_{254}) were used for thin-layer chromatography. Preparative column chromatography was performed using either neutral alumina gel from Fluka (Type 507 C) 100-125 mesh or SilicaFlash® silica gel P60 (0.04–0.063 mm). Size exclusion chromatography was performed with Bio-Beads™ S-X1 Resin.

Spectroscopic and analytical measurements

^{1}H, ^{13}C, ^{19}F, COSY, NOESY, HSQC, HMBC and DOSY NMR spectra were recorded on a Bruker Avance 400 MHz spectrometer equipped with a variable temperature unit. Chemical shifts were given in ppm with respect to tetramethylsilane $Si(CH_3)_4$. Pneumatically-assisted electrospray (ESI) mass spectra were recorded on an Applied Biosystems API 150EX LC/MS System equipped with a Turbo Ionspray source®. HR mass spectra were recorded on a Xevo G2-TOF HRMS instrument equipped with a Zspray™ Lockspray™ ESI | APCI | ESCi® electrospray by Waters™. Elemental analyses were performed by K. L. Paglia from the Microchemical Laboratory of the University of Geneva. Spectrophotometric titrations were performed with a J&M diode array spectrometer (Tidas series) connected to an external computer. Mathematical treatment of the spectrophotometric titrations was performed with factor analysis and with ReactLab™ Equilibria (previously Specfit/32). Absorption spectra in solution were recorded using a Lambda 1050 PerkinElmer spectrometer (quartz cell path length 1 cm, 0.1 cm or 0.01 cm). Emission and excitation spectra were recorded with a Fluorolog (Horiba Jobin-Yvon) instrument, equipped with iHR320, a xenon lamp 450 W illuminator (FL-1039A/40A), a water-cooled photomultiplier tube (PMT Hamamatsu R928 for 200–900 nm range) and a photomultiplier tube (PMT Hamamatsu R5509 for 900–1600 nm range) with its dedicated liquid nitrogen cooler C9940-02. The spectra were corrected for the spectral response of the system. Continuous NIR laser excitation was achieved with a diode laser MLL-H-800-2.5W (801 nm) from Changchun New Industries Optoelectronics Technology Co., Ltd (CNI). A 550/88 nm bandpass filter (Semrock) was placed directly after the sample for visible emission measurements to remove the second-order Rayleigh scattering of the laser line. Crystallographic data was recorded by mounting the crystals on MiTeGen cryoloops with protection oil. X-ray data collections were performed with an Agilent SuperNova Dual diffractometer equipped with a CCD Atlas detector (Cu[Kα] radiation) or a Rigaku synergy S equipped with an hypix detector. The structures were solved in SHELXT by using dual space methods. Full-matrix least square refinements were performed with SHELXLS6 within the Olex2 program. Electronic absorption spectra in the visible and NIR regions were recorded at 293 K from acetonitrile solutions with a Perkin-Elmer Lambda 1050 absorption spectrometer using

quartz cells of 0.1, 1- and 10-mm path length. Solution emission spectra were recorded in non-deuterated acetonitrile (using quartz cells of 10 mm path length. The emission spectra were corrected for the instrumental response function.

The upconversion quantum yields Φ^{UC} of the parent [**L2**Er(hfac)$_3$]$^+$ [28] and [**L3**Er(hfac)$_3$]$^+$ [29] complexes were determined in ref. 28 and 29 through the relative method (eqn A2-1) using indocyanine green as the reference (ICG, λ_{exc} = 801 nm, Φ^{ref} = 0.132 in ethanol at 293 K, $n_{ethanol}$ = 1.361).[A2-1] For the sake of safe comparison purposes, the upconversion quantum yields ϕ^{UC} of [ZnErZn(**L5**)$_3$]$^{10+}$ were obtained by using [**L3**Er(hfac)$_3$]$^+$ as the reference (λ_{exc} = 801 nm, Φ^{ref} = $1.1\cdot10^{-8}$ in acetonitrile at 293 K, $n_{acetonitrile}$ = 1.344)[29] and eqn (A2-1), where Φ is the quantum yield, E is the integrated emission spectrum, A is the absorbance at the excitation wavelength λ, n is the refractive index, P_{exc} is the power intensity of the excitation source at the excitation wavelength and $h\nu_{exc}$ is the energy of the incident photon at frequency $\nu_{exc} = (c/\lambda_{exc})$ so that $I_{exc} = P_{exc}/h\nu_{exc}$ is the spectral radiant power measuring the incident excitation intensity.

$$\frac{\Phi^{UC}}{\Phi^{ref}} = \frac{E_{up}}{E_{ref}} \cdot \frac{A_{ref}}{A_{up}} \cdot \frac{n_{ref}^2}{n_{up}^2} \cdot \frac{P_{exc,ref}}{P_{exc,up}} \cdot \frac{h\nu_{exc,up}}{h\nu_{exc,ref}} \qquad \text{(A2-1)}$$

The geometry of the measuring cell was optimized (position and rotation) so that the laser-diode 801 nm beam went through a width compatible with the detection of transmitted light at 801 nm along the original excitation direction. The detector was then focused on the part of the solution in the cell which was excited by the incident beam. Having fixed all these parameters, the upconversion emission spectra was recorded. The concentration of the reference [**L3**Er(hfac)$_3$]$^+$ was adapted so that its emission intensity was recordable with that of the sample using the same excitation power.

X-ray crystallography

Summary of crystal data, intensity measurements and structure refinements for the ligand [**L5**]$PF_6\cdot C_3H_5N\cdot0.25(C_5H_{12}O)$ were collected in Table S1. Pertinent bond lengths, bond angles and interplanar angles were collected (Table S2) together with ORTEP views (Fig. S7). Crystals were mounted on Hampton cryoloops with protection oil. X-ray data collections were performed with a XtaLAB Synergy-S diffractometer equipped with a hybrid pixel array “hypix arc 150” detector. The structures were solved with ShelXT[A2-2] and all other calculations were performed with SHELXL,[A2-3] OLEX2[A2-4] and ORTEP[A2-5] programs. CCDC 2376478 contains the supplementary crystallographic data for this paper. These data can be obtained free of charge from The Cambridge Crystallographic Data Centre via www.ccdc.cam.ac.uk/data_request/cif.

Comments about crystal structure and refinement: [**L5**]$PF_6\cdot C_3H_5N\cdot0.25(C_5H_{12}O)$ crystallizes in triclinic system (space group *P*-1) and shows some disordered parts due to the flexibility of such a ligand. PF_6 counter ion is also disordered in the structure and is located on two different sites.

Occupancies were fixed 0.5 for each part but one is still slightly disordered around its position. This later was split into 2 parts with fixed occupancies 0.25-0.25. Finally, the total count of PF_6 counter ion is 1. A solvent propionitrile molecule is disordered and occupancies where fixed to the best value that gives reasonable atomic displacement parameters (ADPs). Another disordered propionitrile molecule was found very close to (and slightly superimposed with) a disordered tert-butyl-methyl-ether and a PF_6 ion. Occupancies of both ether and propionitrile were fixed to 0.25 each and were refined with isotropic ADPs. The following chemical formula moiety $C_{87}H_{86}N_{13}S$, F_6P, C_3H_5N, $0.25(C_5H_{12}O)$ was finally obtained.

Synthesis of ligands and complexes

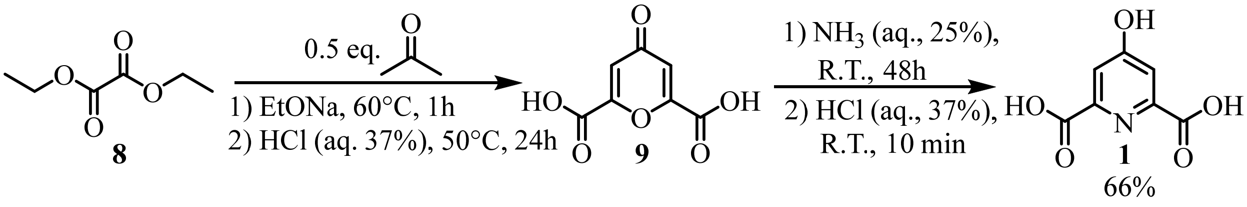

Scheme A2-1 Synthesis of chelidamic acid (**1**).[29]

Preparation of chelidonic acid (9).

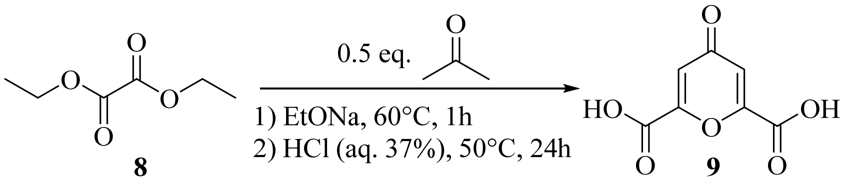

Dry ethanol (1 L) was prepared by adding CaH_2 (2 g) and refluxing overnight under an argon atmosphere. The solvent was then distilled under argon. In a similar way, acetone (50 g, 63.8 mL, 0.86 mol, 1 eq.) was dried by adding K_2CO_3 (20 g) and refluxed overnight under an argon atmosphere. The solvent was then distilled under argon. Sodium (62 g, 2.7 mol, 3.14 eq.) was dissolved in the dry ethanol. Diethyl oxalate (**8**, 267.1 g, 246 mL, 1.83 mol, 2.13 eq.) was added to the distilled acetone and this mixture was added dropwise to the sodium ethanolate solution. A yellow-orange precipitate was formed. The mixture was stirred at 60 °C for 1 hour. Then, HCl (aq. 37%, 530 mL) and water (250 mL) were added, and the solution stirred at 50 °C for 1 day. Around 1 L of ethanol-water mixture was removed under reduced pressure and then a mixture of water (600 mL) and HCl (37%, 140 mL) was added while stirred and heated at 50 °C until a silica gel TLC (eluent: 3/7 (v/v) 10% NaCl/ethanol) showed only one spot for the desired product. After 3 days of reaction, the mixture was cooled to room temperature and filtered off. It was washed a first time with water and then with cold acetone. The crude wet product (296.87 g) was used directly for the next step without any further purification.

A small amount of the crude product (291 mg) was dissolved in boiling water (7 mL) and charcoal was added. The mixture was refluxed for 15 minutes and then filtered over celite (diatomaceous earth) and gave 56.4 mg of pure chelidonic acid (**9**) as a beige powder.

^{1}H NMR (400 MHz, DMSO-d_6) δ 6.87 (s, 2H).

ESI-MS ($CHCl_3$, soft pos. mode): *m/z* calcd for $C_7H_5O_6$ $[M+H]^+$ 185.0, found 185.0.

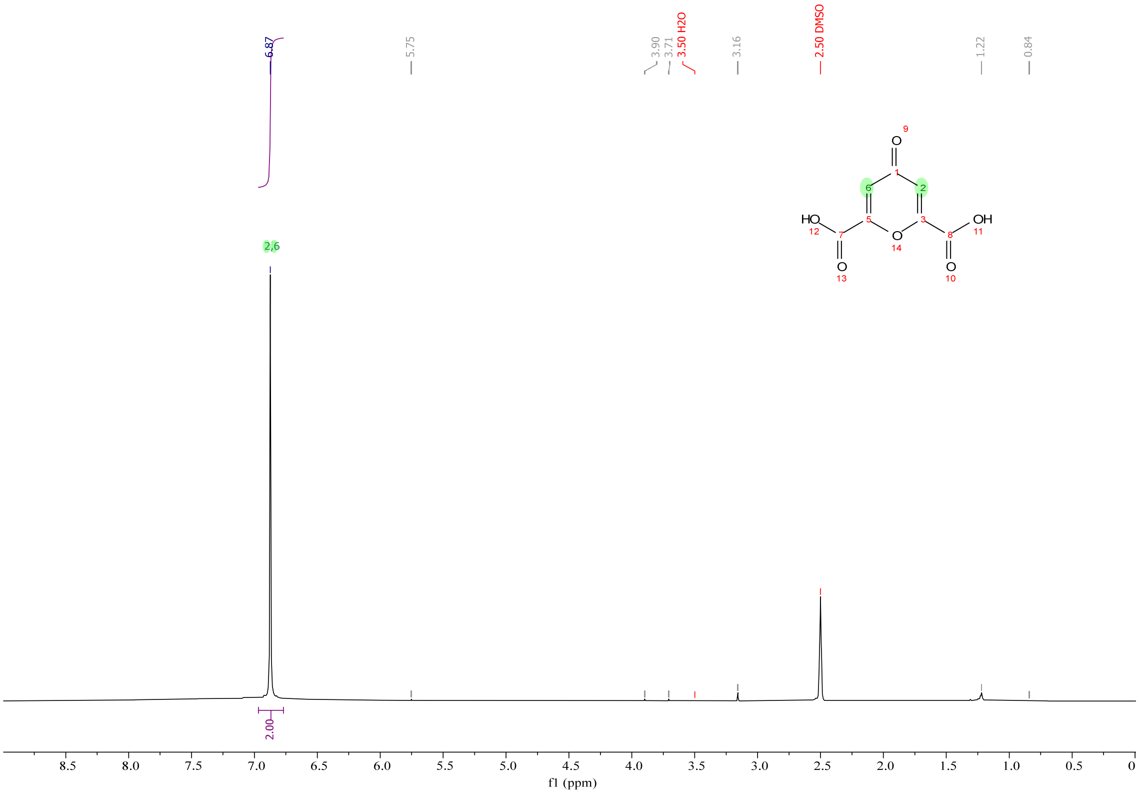

Fig. A2-1. ^{1}H NMR (400 MHz) of **9** in DMSO-d_6.

Preparation of chelidamic acid (1)

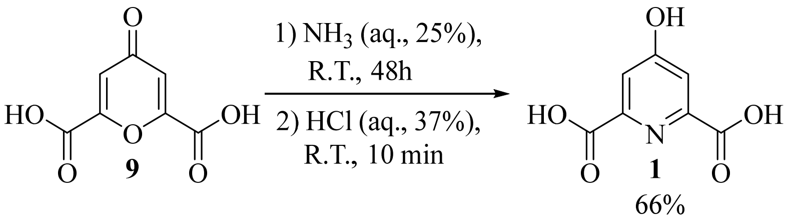

The crude chelidonic acid (**9**) was put in a 2 L flask and aqueous hydroxide ammonium (NH_3 25%, 1.5 L) was added dropwise while keeping it stirred at 0 °C. It was then stirred for 48 hours at room temperature. The mixture went from orange to beige after about 1 hour and finally turned to dark orange over the first night. The excess of ammonia was removed completely by boiling the mixture and the released gas was quenched over water trap. Charcoal (30 g) was added to the mixture and boiled for 1 hour. The mixture was then filtered and cooled to about 5 °C. The solution was then acidified to pH = 1 with a conc. HCl solution (37%). The pale brown crystals (expected white) were

filtered off and washed several times with ice-cold water and dried overnight under vacuum at 50 °C to give chelidamic acid (**1**, 103.633 g, yield over 2 steps: 66%).

A part of the crude product (25.60 g) was purified by dissolving it in 200 mL of NH_4OH (25%) and the mixture was kept at pH = 9–10. The solution was washed with CH_2Cl_2 (3x150 mL), the aqueous phase was isolated and the product was precipitated by adding an HCl solution (37%) until pH = 1 and stirred for 10 min. The crystals were filtered, washed with ice-cold water and then dried overnight under vacuum at 65 °C to give 21.83 g of chelidamic acid.

^{1}H NMR (400 MHz, DMSO-d_6) δ 7.55 (s, 2H).

ESI-MS ($CHCl_3$, soft pos. mode): *m/z* calcd for $C_7H_6NO_5$ $[M+H]^+$ 184.0, found 184.0.

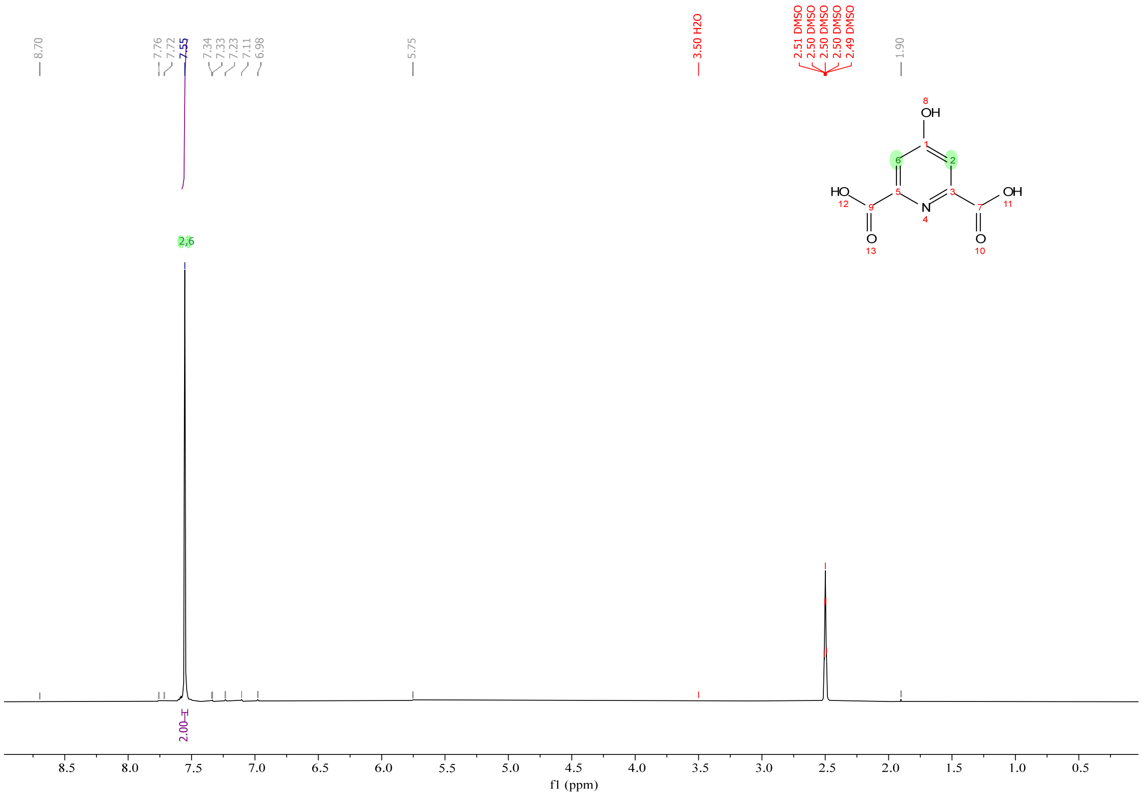

Fig. A2-2 ^{1}H NMR (400 MHz) of **1** in DMSO-d_6.

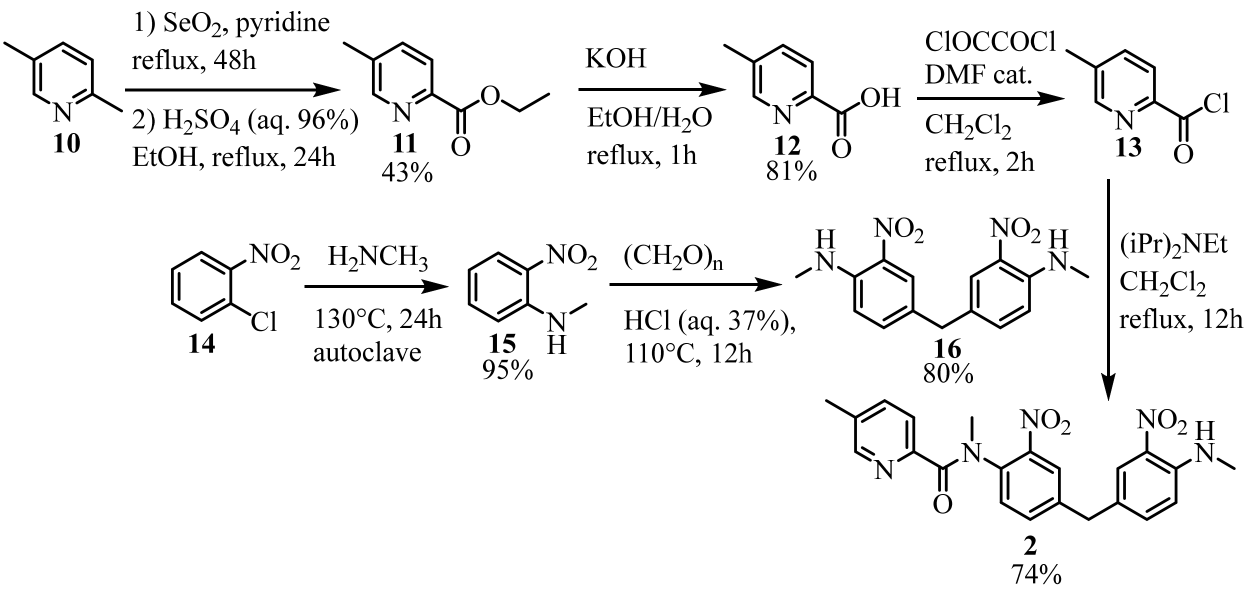

Scheme A2-2 Synthesis of N,5-dimethyl-N-(4-(4-(methylamino)-3-nitrobenzyl)-2-nitrophenyl)picolinamide (**2**).[84]

Preparation of *N*-methyl-2-nitroaniline (15).

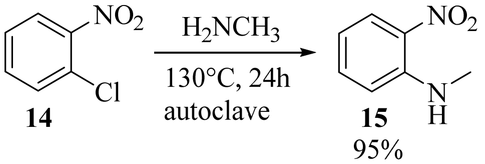

1-Chloro-2-nitrobenzene (**14**, 30.69 g, 194.8 mmol, 1 eq.) and methylamine (195 mL, 40% in water, 1.9 eq.) were heated in an autoclave at 130 °C for 24 h. The dark mixture (2 phases: red on top and black at the bottom) was evaporated to dryness, partitioned between dichloromethane (150 mL) and aqueous NH_4Cl solution (150 mL, half-saturated), the organic layer was separated and the aqueous phase further extracted with CH_2Cl_2 (2 x 100 mL). The combined organic phases were dried over Na_2SO_4, evaporated to dryness and the crude product purified by column chromatography (Silicagel, CH_2Cl_2 100%) to give **15** (28.19 g, 185.3 mmol, yield: 95%).

^{1}H NMR (400 MHz, CD_2Cl_2) δ 8.14 (dd, *J* = 8.6, 1.6 Hz, 1H), 7.99 (br s, 1H), 7.49 (dddd, *J* = 8.6, 6.9, 1.7, 0.7 Hz, 1H), 6.88 (dd, *J* = 8.7, 1.3 Hz, 1H), 6.66 (ddd, *J* = 8.4, 6.9, 1.3 Hz, 1H), 3.02 (d, *J* = 5.1 Hz, 3H).

ESI-MS ($CHCl_3$, soft pos. mode): *m/z* calcd for $C_7H_9N_2O_2$ $[M+H]^+$ 153.1, found 152.4.

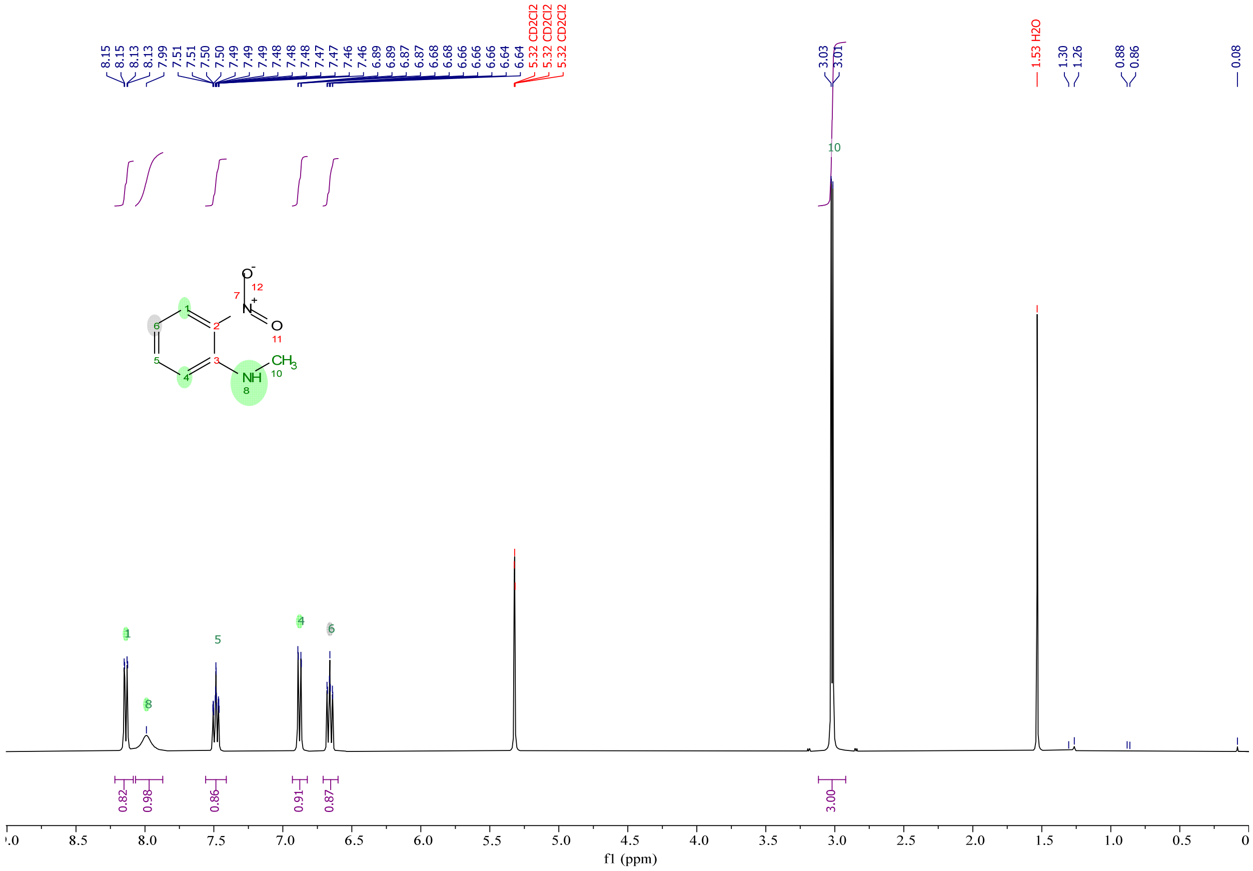

Fig. A2-3 ^{1}H NMR (400 MHz) of **15** in CD_2Cl_2.

Preparation of 4,4'-methylene-2,2'-dinitro-bis(N-methyl-aniline) (16)

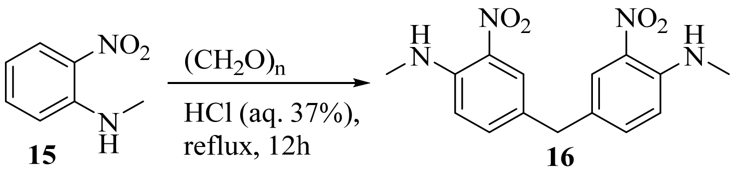

N-methyl-2-nitro-aniline (**15**, 6.28 g, 41 mmol), paraformaldehyde (0.6144 g, 21 mmol) and concentrated HCl (aq. 37%, 63 mL) were stirred at room temperature for 2 h, under an inert atmosphere, then heated progressively: 1 h at 40 °C, 1 h at 60 °C, and then 12 h at reflux (115 °C). After cooling the mixture at room temperature, water (200 mL) was added, and the excess of acid was neutralized to pH = 9 with 24% of aq. NH_4OH. The resulting mixture was extracted with dichloromethane (3×100 mL). The combined organic layers were dried (Na_2SO_4), evaporated to dryness and the crude product purified by column chromatography (Silicagel, CH_2Cl_2/cyclohexane 70:30) to give **16** as a red crystalline powder (5.1938 g, yield: 80%).

^{1}H NMR (400 MHz, CD_2Cl_2) δ 7.97 (dq, *J* = 2.0, 0.7 Hz, 2H), 7.95 (s, 2H), 7.76 (d, *J* = 2.3 Hz, 0H), 7.32 (ddd, *J* = 8.8, 2.3, 0.6 Hz, 2H), 6.84 (d, *J* = 8.9 Hz, 2H), 3.83 (s, 2H), 3.00 (d, *J* = 5.1 Hz, 6H). ESI-MS ($CHCl_3$, soft pos. mode): *m/z* calcd for $C_{15}H_{17}N_4O_4$ $[M+H]^+$ 317.1, found 317.5.

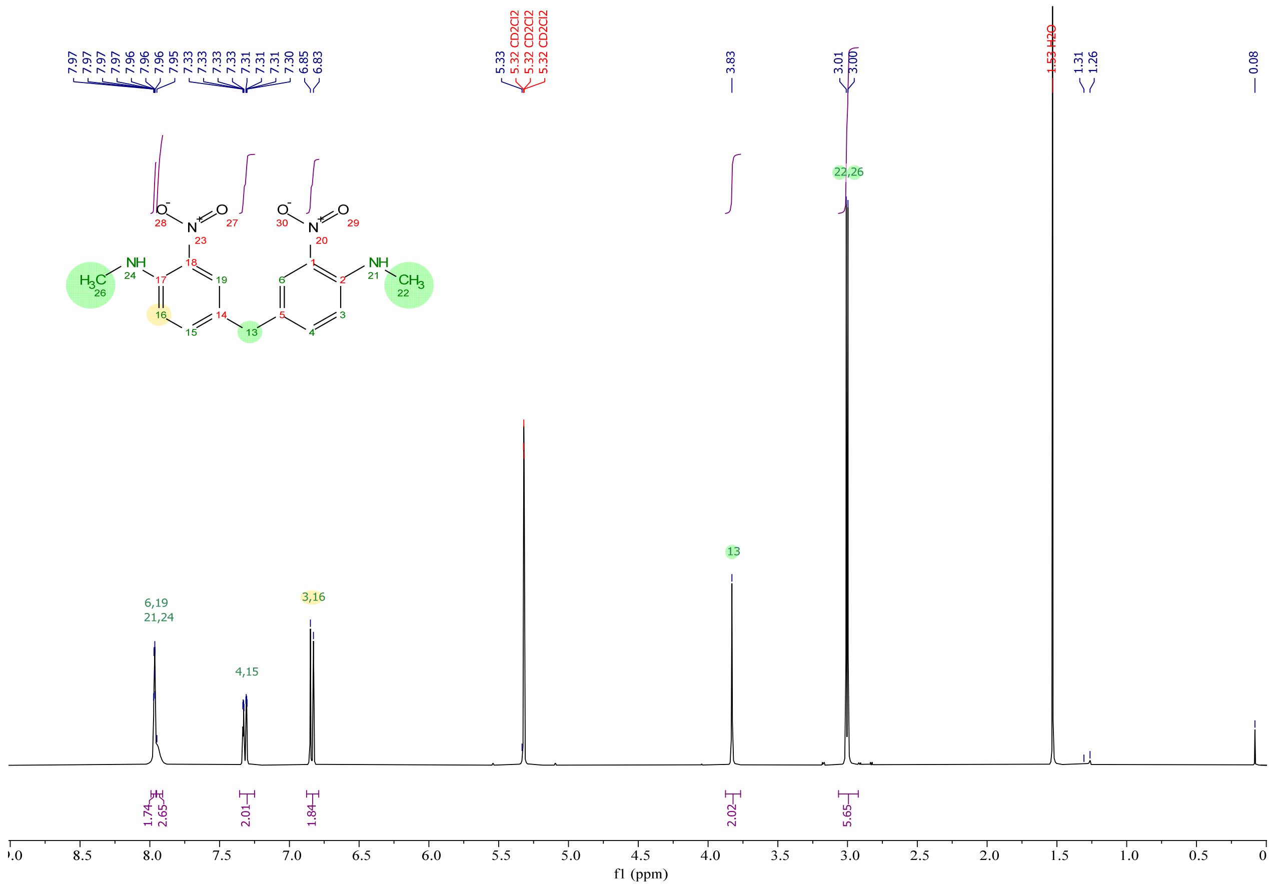

Fig. A2-4 ^{1}H NMR (400 MHz) of **16** in CD_2Cl_2.

Preparation of 5-methyl-2-pyridinecarboxylic acid-ethyl ester (11)

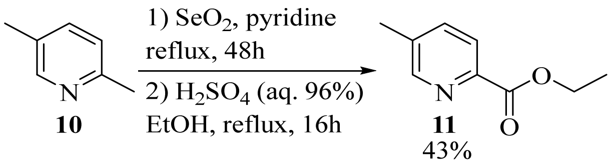

2,5-lutidine (**10**, 15.0 g, 16.2 mL, 140 mmol, 1 eq.) and selenium dioxide (23.24 g, 209 mmol, 1.5 eq.) were dissolved in pyridine (45 mL) and refluxed for 48 h. The mixture was cooled to room temperature and filtered over celite to remove the excess selenium dioxide and washed with pyridine (60 mL) and distilled water (60 mL). The pyridine was removed from the mixture under reduced pressure and water was added several times to help the evaporation by the formation of azeotrope. The resulting black oil/wax was suspended in ethanol (600 mL) and concentrated H_2SO_4 (aq. 96%, 12 mL). The mixture was refluxed overnight (16 h). Distilled water (50 mL) was added and the solution was neutralized by addition of NaOH (aq. 5 M). Ethanol was removed by rotavapor and the remaining aqueous solution was extracted with CH_2Cl_2 (4 x 100 mL). The combined organic fractions were dried (Na_2SO_4), filtered and evaporated to dryness. The residual black oil was distilled through a small Vigreux column (T_{vapour} = 62–65 °C, $2.4 \cdot 10^{-2}$ mbar) to give **11** as a colourless liquid (9.94 g, 60.2 mmol, yield: 43%).

^{1}H NMR (400 MHz, Chloroform-*d*) δ 8.53 (dt, *J* = 2.2, 0.8 Hz, 1H), 7.99 (dd, *J* = 8.0, 0.8 Hz, 1H), 7.59 (ddq, *J* = 8.0, 2.3, 0.8 Hz, 1H), 4.43 (q, *J* = 7.1 Hz, 2H), 2.37 (d, *J* = 1.0 Hz, 3H), 1.40 (t, *J* = 7.1 Hz, 3H).

ESI-MS (CH_3CN, soft pos. mode): *m/z* calcd for $C_9H_{12}NO_2$ $[M+H]^+$ 166.1, found 166.1.

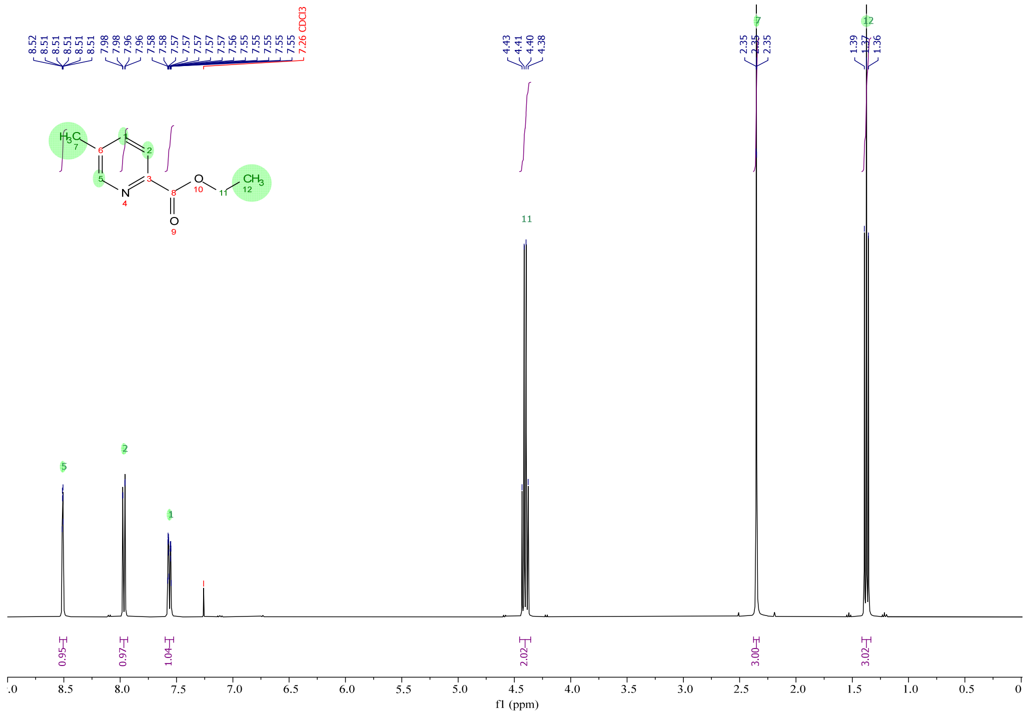

Fig. A2-5 ^{1}H NMR (400 MHz) of **11** in $CDCl_3$.

Preparation of 5-methyl-2-pyridinecarboxylic acid (12)

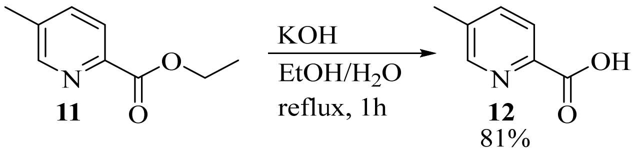

5-Methyl-2-pyridinecarboxylic acid-ethyl ester (**11**, 9.83 g, 59.5 mmol) was dissolved in ethanol (100 mL) and KOH (7.33 g in 100 mL water). The mixture was refluxed for 1 h, and ethanol was evaporated. The solution was washed with CH_2Cl_2 (3×50 mL), acidified with HCl (aq. 37%) to pH = 3.5 and evaporated to dryness under vacuum. The residual solid was dissolved in 600 mL of ethylacetate, refluxed for 3 h and filtered while hot. The volume of the filtrate was reduced by rotatory evaporation until precipitation. After cooling at −20 °C for 12 h, the precipitate was separated by filtration. The volume of the remaining organic phase was again reduced, cooled and filtered and this procedure was repeated several times to give **12** as a white solid (6.5983 g, 48.1 mmol, yield: 81%).

^{1}H NMR (400 MHz, Chloroform-*d*) δ 8.53 (d, *J* = 2.1 Hz, 1H), 8.16 (d, *J* = 7.9 Hz, 1H), 7.82 – 7.75 (m, 1H), 2.50 (s, 3H).

ESI-MS ($CHCl_3$, soft pos. mode): *m/z* calcd for $C_7H_8NO_2$ $[M+H]^+$ 138.0, found 138.4.

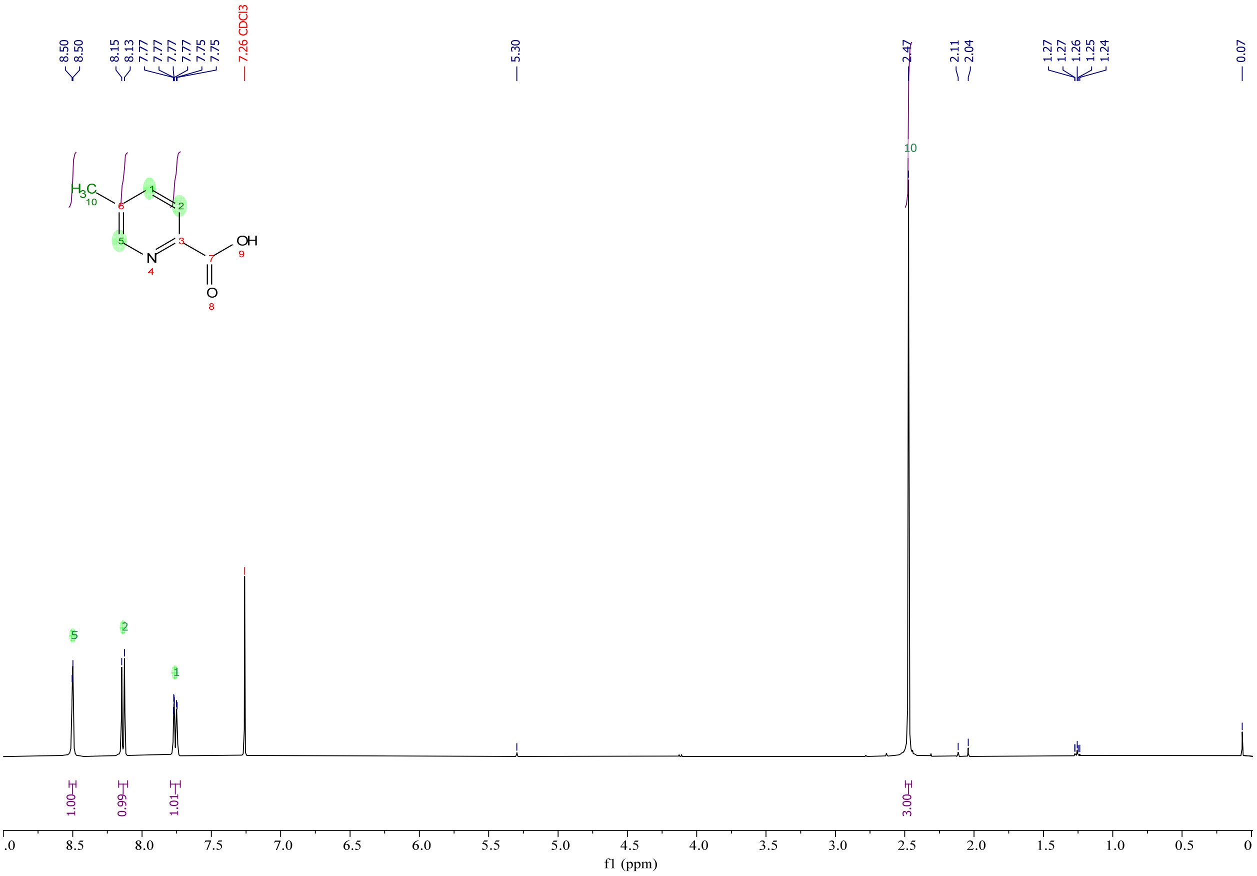

Fig. A2-6 ^{1}H NMR (400 MHz) of **12** in $CDCl_3$.

Preparation of 5-methyl-2-picolinoyl chloride (13)

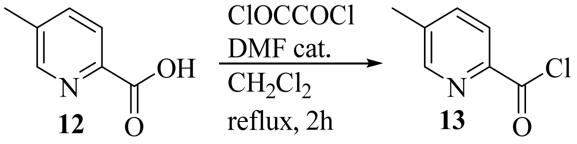

To a suspension of 5-methyl-2-pyridinecarboxylic acid (**12**, 1.7044 g, 12.39 mmol, 1 eq.) in dry CH_2Cl_2 (35 mL) oxalyl chloride (4.3 mL, 50.13 mmol, 4 eq.) and a catalytic amount of DMF (5 drops) were added dropwise under an inert atmosphere. The stirred solution was refluxed for 2 h and then evaporated to dryness to give **13** as a brown-black solid (1.93 g, quant.). Compound **13** was directly used without further purification or analysis.

Preparation of *N*,5-dimethyl-*N*-(4-(4-(methylamino)-3-nitrobenzyl)-2-nitrophenyl)picolinamide (2)

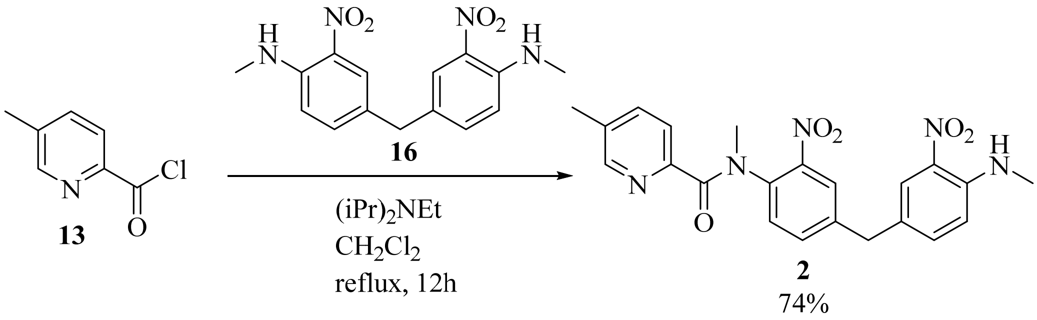

To a solution of **16** (12.1715 g, 38.48 mmol, 3 eq.) in dry CH_2Cl_2 (120 mL) was added dropwise a solution of **13** (1.93 g, 12.41 mmol, 1 eq.) in dry CH_2Cl_2 (60 mL), under an inert atmosphere. In the middle of the addition, (*i*-Pr)$_2$NEt (1.1 mL) was added to the mixture. The mixture was stirred at reflux and a second crop of (*i*-Pr)$_2$NEt (3.2 mL) was progressively added. After 12h, the mixture was evaporated to dryness affording a black oil. The oil was suspended in CH_2Cl_2 (50 mL) and an aqueous solution of NH_4Cl (50 mL, half-saturated). The organic phase was isolated and the aqueous phase was extracted with CH_2Cl_2 (3x50 mL). The combined organic phases were dried over Na_2SO_4, filtered and evaporated to dryness affording an orange oil. The crude product was purified by column chromatography (Silicagel, CH_2Cl_2/MeOH 100:0 → 99:1) to give **2** as an orange powder (3.92 g, 9.00 mmol, yield: 74%).

^{1}H NMR (400 MHz, Methylene Chloride-*d*$_2$) δ 8.01 (s, 1H), 7.96 (t, *J* = 2.7 Hz, 2H), 7.72 (d, *J* = 2.1 Hz, 1H), 7.64 (d, *J* = 8.0 Hz, 1H), 7.53 – 7.47 (m, 1H), 7.37 (dd, *J* = 8.2, 2.1 Hz, 1H), 7.32 (dd, *J* = 8.8, 2.2 Hz, 1H), 7.27 (d, *J* = 8.1 Hz, 1H), 6.89 (d, *J* = 8.8 Hz, 1H), 3.98 (s, 2H), 3.46 (s, 3H), 3.05 (d, *J* = 5.1 Hz, 3H), 2.26 (s, 3H).

ESI-MS (CH_3CN, soft pos. mode): *m/z* calcd for $C_{22}H_{22}N_5O_5$ $[M+H]^+$ 436.2, found 436.1.

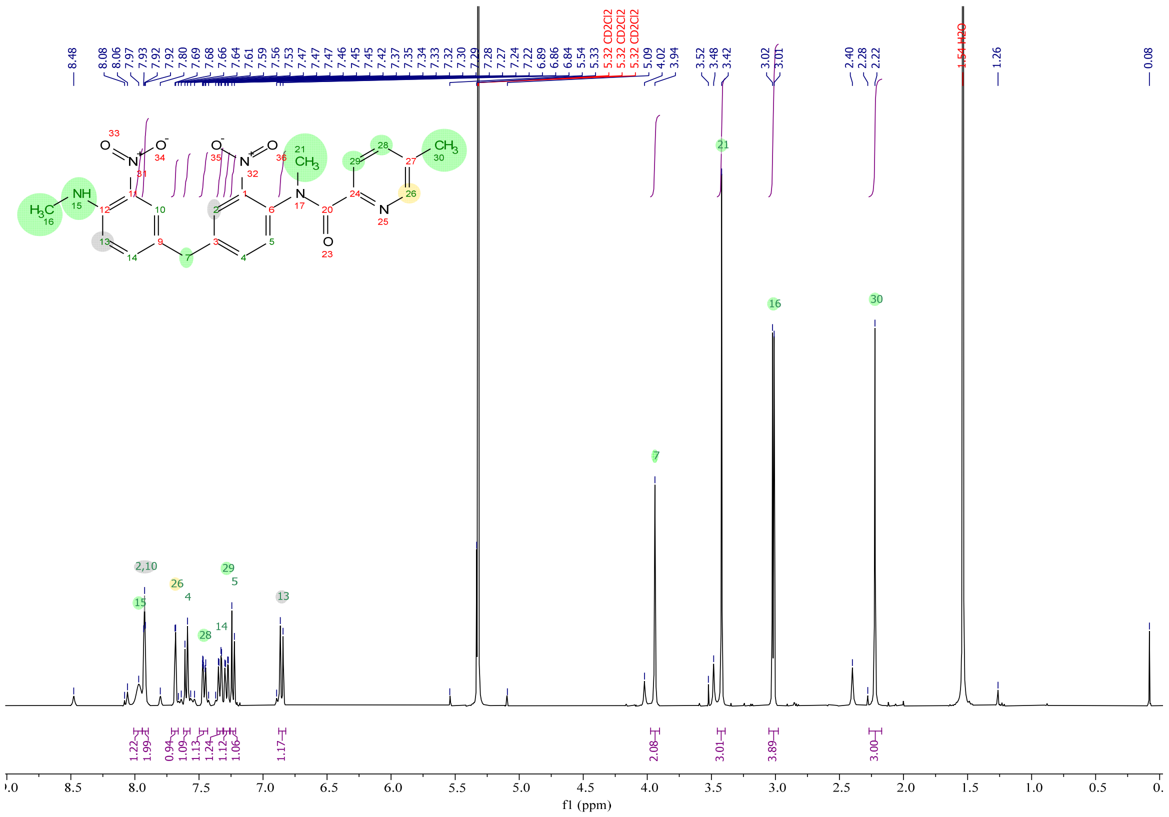

Fig. A2-7 ^{1}H NMR (400 MHz) of **2** in CD_2Cl_2.

Preparation of 4-bromo-*N*^{2},*N*^{6}-bis(4-(4-(*N*,5-dimethylpicolinamido)-3-nitrobenzyl)-2-nitrophenyl)-*N*^{2},*N*^{6}-dimethylpyridine-2,6-dicarboxamide (3)

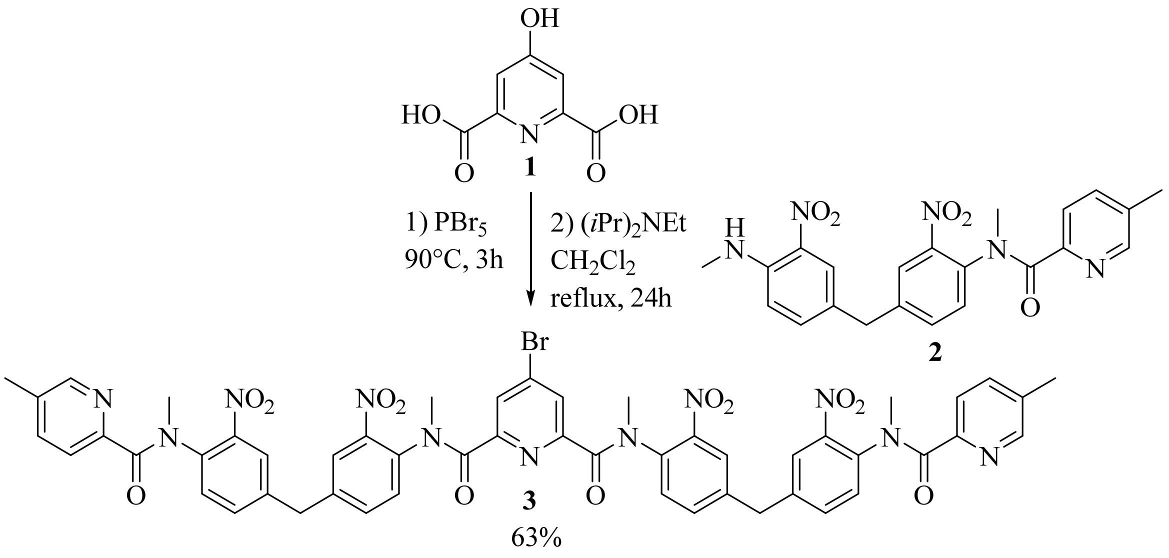

Chelidamic acid (**1**, 900.9 mg, 4.92 mmol) was added to a 50 mL Schlenk flask containing PBr_5 (6.97 g, 16.19 mmol, 3 eq.). The temperature was raised to 90 °C for 3 h under an inert atmosphere. The mixture was cooled to room temperature and dissolved with dry CH_2Cl_2 (60 mL) and filtered (under inert atmosphere) and directly added to a solution of **2** (4.27 g, 9.81 mmol, 2.1 eq.) and (*i*-Pr)$_2$NEt (18 mL) in dry CH_2Cl_2 (60 mL). The mixture was stirred at reflux for 24h. The reaction mixture was evaporated to dryness and the product was suspended in CH_2Cl_2 (50 mL) and washed with an aqueous

solution of NH_4Cl (half-saturated, 50 mL). The organic phase was isolated, dried over Na_2SO_4, filtered and evaporated to dryness. The crude product was purified by column chromatography (SiO_2, CH_2Cl_2/MeOH 100 : 0 → 98 : 2) to give **3** (3.32 g, 3.07 mmol, yield: 63%) as a yellow solid (unreacted **2** was recovered). ^{1}H- and ^{13}C-NMR (400/100 MHz, CD_2Cl_2) show the co-existence of many conformers at room temperature which prevents the full attribution of the spectra.

ESI-MS (CH_3OH/formic acid 0.1%, soft pos. mode): *m/z* calcd for $C_{51}H_{43}BrN_{11}O_{12}$ $[M+H]^+$ 1082.2 (^{81}Br) and 1080.2 (^{79}Br), found 1082.4 and 1080.4.

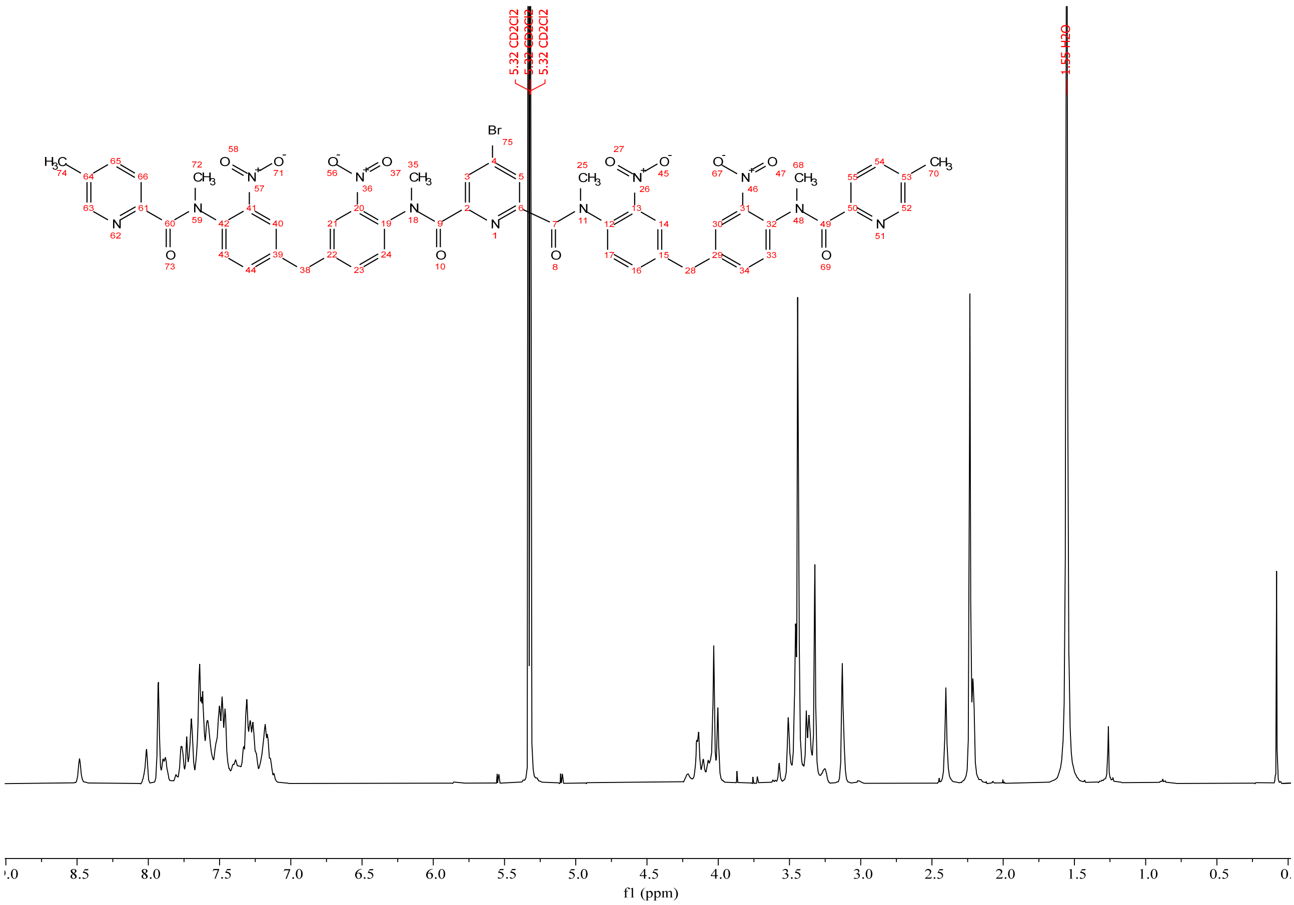

Fig. A2-8 ^{1}H NMR (400 MHz) of **3** in CD_2Cl_2. Attribution was not possible because of the presence of different conformers in the NMR timescale.

Preparation of Br-(extd)bzpy (4)

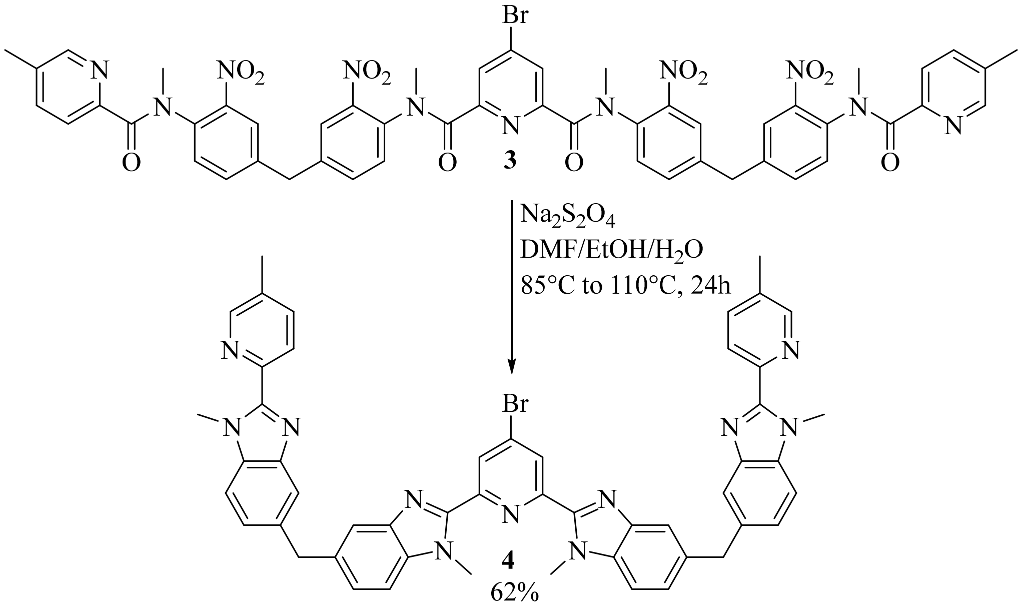

A solution of **3** (3.32 g, 3.07 mmol, 1 eq.) and $Na_2S_2O_4$ (5.5 g, 31.59 mmol, 10 eq.) in DMF (26 mL) and EtOH (130 mL) was stirred and warm up to 85 °C. Then, H_2O (130 mL) was added dropwise and the mixture was stirred at reflux (110 °C) for 24h. The reaction mixture was cooled to room temperature and a 1M Na_2CO_3 solution was added (to reach pH = 9–10). The reaction mixture was then evaporated to dryness. The resulting solid was suspended in CH_2Cl_2 (300 mL), mixed thoroughly in an ultrasonic bath at 35 °C during 30 minutes and filtered roughly on Büchner. The resulting solution was centrifuged (11'000 rpm, 11 minutes) and the clear supernatant was separated. The solution was evaporated to dryness and the crude product was dissolved in a minimum amount of CH_2Cl_2 and added to cold MeCN to precipitate the product. The beige solid was filtered over a nylon membrane, washed with cold MeCN and dried to give Br-(extd)bzpy (**4**, 1.68 g, 1.89 mmol, yield: 62%).

^{1}H NMR (400 MHz, Methylene Chloride-d_2) δ 8.59 (s, 2H), 8.55 – 8.45 (m, 2H), 8.23 (dd, J = 8.0, 0.8 Hz, 2H), 7.68 (dd, J = 1.6, 0.8 Hz, 2H), 7.67 – 7.64 (m, 2H), 7.62 (dd, J = 1.6, 0.7 Hz, 2H), 7.39 (dd, J = 8.4, 0.7 Hz, 2H), 7.35 (dd, J = 8.4, 0.7 Hz, 2H), 7.28 (dd, J = 8.4, 1.6 Hz, 2H), 7.22 (dd, J = 8.3, 1.6 Hz, 2H), 4.27 (s, 4H), 4.22 (s, 6H), 4.21 (s, 6H), 2.40 (s, 6H).

^{13}C NMR (101 MHz, CD_2Cl_2) δ 151.17, 151.06, 149.48, 149.33, 148.64, 143.40, 143.37, 137.67, 137.47, 136.58, 136.47, 136.44, 134.49, 134.06, 128.17, 125.62, 124.65, 124.42, 120.28, 119.90, 110.37, 110.18, 42.56, 33.05, 33.02, 18.59.

ESI-MS ($CHCl_3$, soft pos. mode): *m/z* calcd for $C_{51}H_{43}BrN_{11}$ $[M+H]^+$ 890.3 (^{81}Br) and 888.3 (^{79}Br), found 890.1 and 888.4.

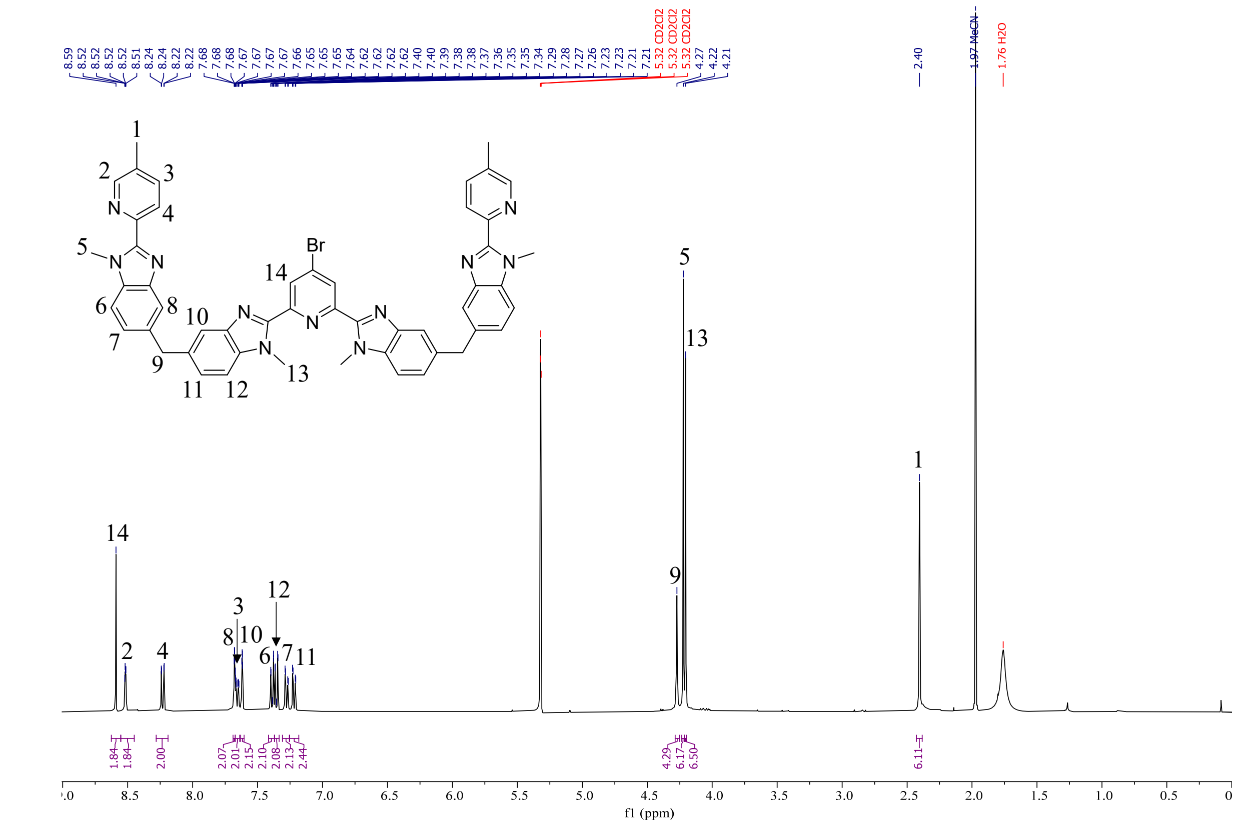

Fig. A2-9 ^{1}H NMR (400 MHz) of **4** in CD_2Cl_2.

Preparation of SMe-(extd)bzpy (5)

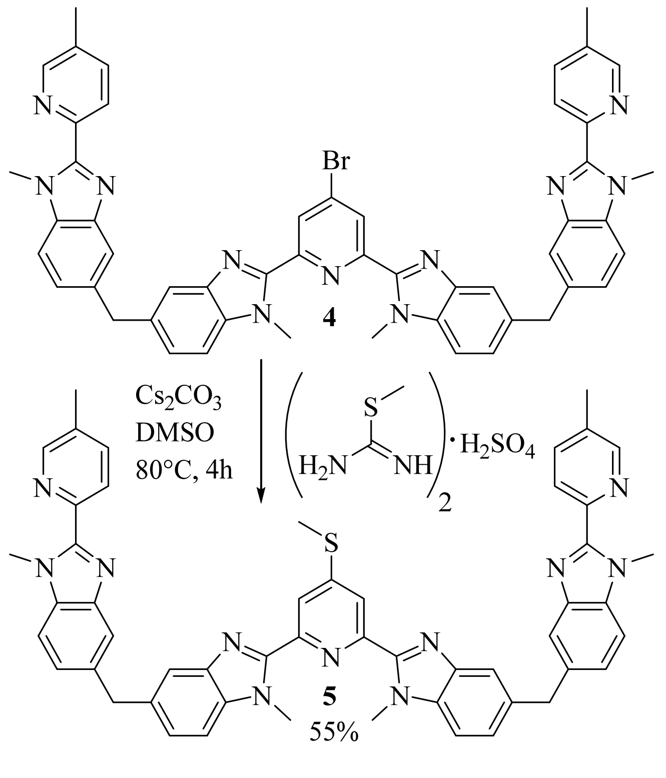

Br-(extd)bzpy (**4**, 0.36 g, 0.41 mmol, 1 eq.), *S*-methylisothiourea sulfate (0.2281 g, 0.81 mmol, 2 eq.) and cesium carbonate (0.5571 g, 1.7 mmol, 4 eq.) were dissolved in dry DMSO (5 mL). The mixture was mixed under inert atmosphere and heated at 80 °C during 4h. After cooling down to room

temperature, the mixture was poured into water (20 mL) and extracted with CH_2Cl_2 (3x25 mL). The combined organic phases were washed with brine (20 mL), dried over anhydrous Na_2SO_4, filtered and evaporated to dryness. The remaining solid was dissolved in a minimum amount of CH_2Cl_2 and precipitated by pouring in cold MeCN (5 mL). The whitish solid was filtered on nylon membrane and dried under vacuum to give SMe-(extd)bzpy (**5**, 0.1909 g, 0.223 mmol, yield: 55%).

^{1}H NMR (400 MHz, Methylene Chloride-*d*$_2$) δ 8.57 – 8.41 (m, 2H), 8.24 (dd, *J* = 8.1, 0.9 Hz, 2H), 8.20 (s, 2H), 7.67 (d, *J* = 0.7 Hz, 2H), 7.68 – 7.64 (m, 2H), 7.62 (d, *J* = 0.8 Hz, 2H), 7.37 (ddd, *J* = 9.9, 8.4, 0.7 Hz, 4H), 7.26 (dd, *J* = 8.4, 1.6 Hz, 2H), 7.22 (dd, *J* = 8.3, 1.6 Hz, 2H), 4.27 (s, 4H), 4.22 (s, 6H), 4.20 (s, 6H), 2.66 (s, 3H), 2.41 (s, 6H).

^{13}C NMR (101 MHz, CD_2Cl_2) δ 153.15, 151.04, 150.62, 149.69, 149.32, 148.64, 143.38, 143.34, 137.67, 137.21, 136.68, 136.42, 136.39, 134.05, 125.24, 124.67, 124.42, 120.97, 120.09, 119.88, 110.28, 110.16, 42.57, 33.04, 32.99, 18.59, 14.30.

ESI-MS ($CHCl_3$, soft pos. mode): *m/z* calcd for $C_{52}H_{46}N_{11}S$ $[M+H]^+$ 856.4, found 856.5.

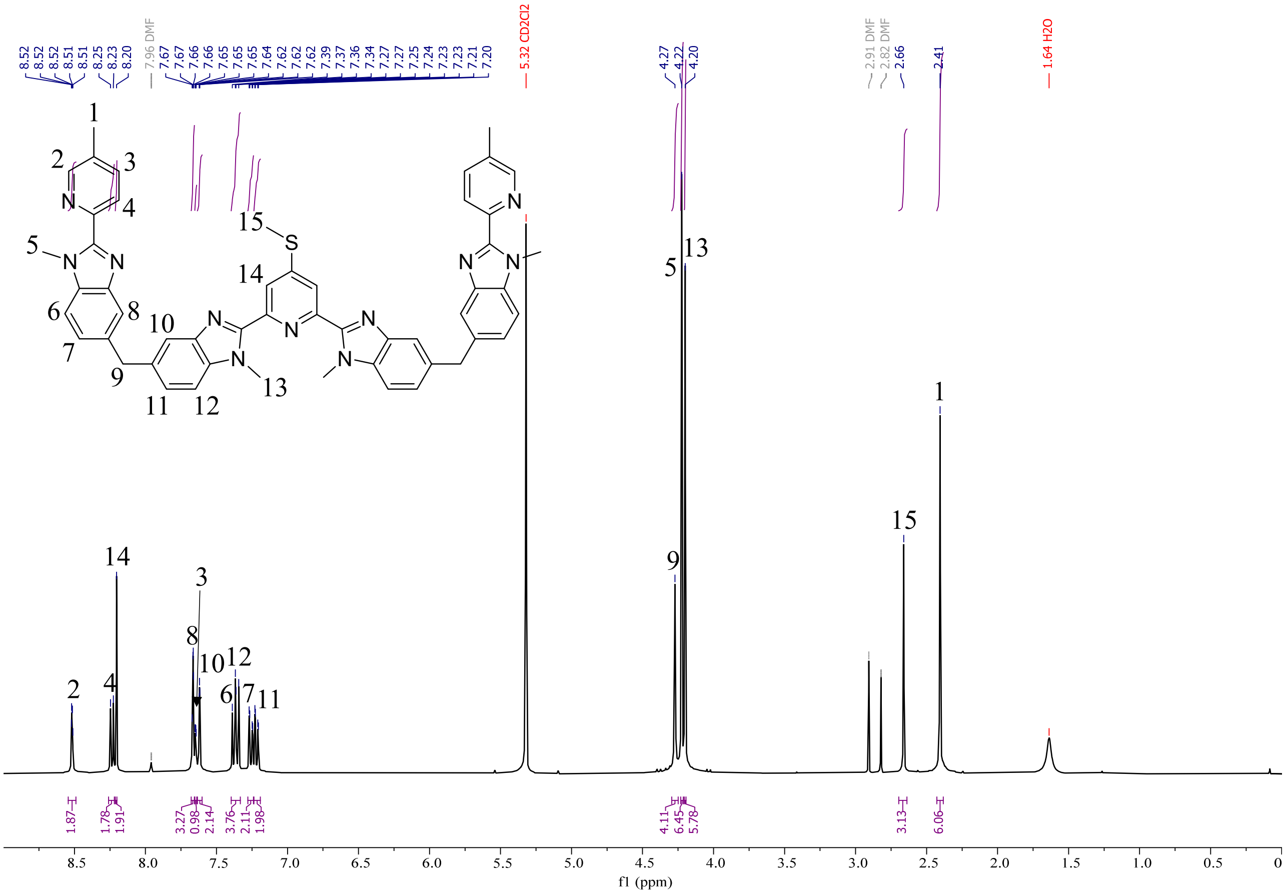

Fig. A2-10 ^{1}H NMR (400 MHz) of **5** in CD_2Cl_2.

Preparation of SH-(extd)bzpy (6)

SMe-(extd)bzpy (**5**, 0.1307 g, 0.15 mmol, 1 eq.), *t*-BuOK (0.1369 g, 1.22 mmol, 8 eq.) were dissolved in dry DMF (2 mL) under inert atmosphere. To the mixture, *t*-BuSH (0.069 g, 0.086 mL, 0.77 mol, 5 eq.) was injected and the reaction was heated to reflux during 16h. The mixture was cooled down in an ice-bath and then poured in a quasi-saturated NH_4Cl aqueous solution (40 mL, water/saturated NH_4Cl aq. Solution 1:4, pH = 5) and a pale yellow solid precipitated. This solid was filtered on nylon membrane, washed several times with ice-cold water and dried under vacuum to give SH-(extd)bzpy (**6**, 0.096 g, 0.114 mmol, yield: 75%).

^{1}H NMR (400 MHz, Methylene Chloride-*d*$_2$) δ 8.63 – 8.44 (m, 2H), 8.27 (s, 2H), 8.23 (dd, *J* = 8.0, 0.9 Hz, 2H), 7.66 (d, *J* = 1.4 Hz, 2H), 7.65 (dd, *J* = 8.4, 2.3 Hz, 2H), 7.62 (dd, *J* = 1.6, 0.8 Hz, 2H), 7.36 (ddd, *J* = 9.1, 8.3, 0.7 Hz, 4H), 7.26 (dd, *J* = 8.4, 1.6 Hz, 2H), 7.22 (dd, *J* = 8.3, 1.6 Hz, 2H), 4.27 (s, 4H), 4.22 (s, 6H), 4.19 (s, 6H), 2.40 (s, 6H).

^{13}C NMR (101 MHz, CD_2Cl_2) δ 151.24, 150.99, 150.11, 149.33, 148.57, 143.29, 143.25, 137.67, 137.31, 136.68, 136.42, 136.39, 134.48, 134.09, 125.39, 124.69, 124.44, 123.88, 120.13, 119.84, 110.32, 110.18, 42.56, 33.05, 33.01, 30.72, 18.59.

ESI-MS ($CHCl_3$, soft pos. mode): *m/z* calcd for $C_{51}H_{44}N_{11}S$ [M+H]$^+$ 842.3, found 842.2.

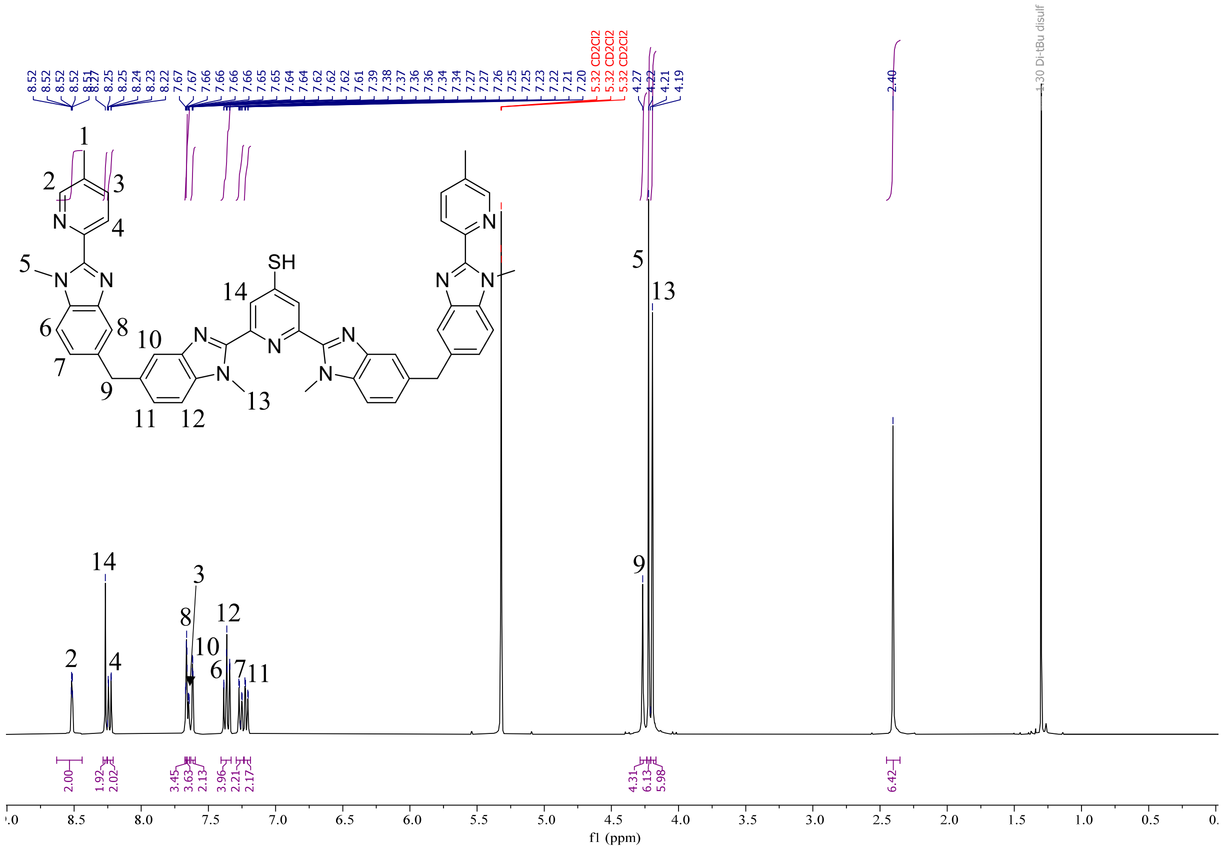

Fig. A2-11 ^{1}H NMR (400 MHz) of **6** in CD_2Cl_2.

Preparation of Sdye-(extd)bzpy [L5]PF_6

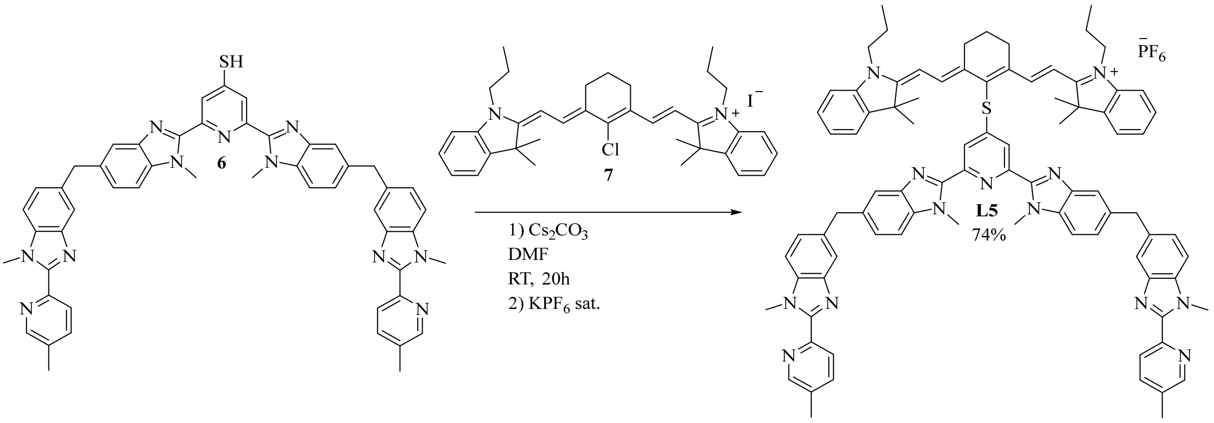

SH-(extd)bzpy (**6**, 0.09197 g, 0.11 mmol, 1.1 eq.), cyanine [IR-780]I (**7**, 0.06552 g, 0.098 mmol, 1 eq.) and Cs_2CO_3 (0.108 g, 0.14 mmol, 1.4 eq.) were dissolved in dry DMF (3 mL) under inert atmosphere. The mixture was stirred at room temperature during 20h. The yellowish solution was then evaporated to dryness and dissolved back in CH_2Cl_2 (15 mL). A saturated aqueous solution of potassium hexafluorophosphate (10 mL) was added and gently shacked. The organic phase was separated, and the aqueous phase was extracted with CH_2Cl_2 (2x15 mL). The combined organic phases were washed with water (3x15 mL), dried over Na_2SO_4, filtered and evaporated to dryness. The solid was dissolved in a minimum amount of MeCN, filtered and precipitated by slow addition of Et_2O. The solid was filtered, washed with Et_2O and dried under vacuum to give Sdye-(extd)bzpy as a green powder ([**L5**]PF_6, 0.108 g, 0.0724 mmol, yield: 74%). Layering of *tert*-butyl methyl ether on a mM propionitrile solution of the latter powder in a closed NMR tube led to precipitation as an irregular deposit on the glass tube. The tube was opened and upon evaporation of the solvents, suitable for X-ray diffraction crystals of [**L5**]PF_6 were formed around one of the spots of precipitate.

^{1}H NMR (400 MHz, Methylene Chloride-d_2) δ 8.70 (d, J = 14.1 Hz, 2H), 8.56 – 8.51 (m, 2H), 8.25 (s, 2H), 8.23 (dd, J = 8.1, 0.8 Hz, 2H), 7.67 (ddd, J = 8.2, 2.3, 0.8 Hz, 2H), 7.64 (dd, J = 1.6, 0.8 Hz, 2H), 7.60 (dd, J = 1.6, 0.7 Hz, 2H), 7.39 – 7.32 (m, 6H), 7.32 – 7.27 (m, 4H), 7.24 – 7.18 (m, 4H), 7.08 (d, J = 8.0 Hz, 2H), 6.17 (d, J = 14.2 Hz, 2H), 4.27 (s, 4H), 4.23 (s, 6H), 4.12 (s, 6H), 3.95 (t, J = 7.5 Hz, 4H), 2.86 – 2.74 (m, 4H), 2.41 (d, J = 1.0 Hz, 6H), 2.18 – 2.07 (m, 2H), 1.84 (h, J = 7.4 Hz, 4H), 1.65 – 1.36 (m, 12H), 1.00 (t, J = 7.4 Hz, 6H).

^{13}C NMR (100 MHz, CD_2Cl_2) δ 173.39, 151.13, 151.00, 150.47, 150.01, 149.38, 148.57, 148.11, 146.05, 143.40, 143.31, 142.52, 141.62, 137.68, 137.50, 136.60, 136.44, 136.39, 134.16, 133.90, 129.05, 125.88, 125.61, 124.60, 124.38, 122.75, 121.66, 120.05, 119.85, 111.20, 110.36, 110.19, 101.99, 49.83, 46.42, 42.54, 33.07, 32.98, 28.10, 26.84, 21.21, 18.60, 11.69.

ESI-MS ($CHCl_3$, soft pos. mode): *m/z* calcd for $C_{87}H_{86}N_{13}S$ $[M]^+$ 1344.7, found 1344.9.

HR-MS ESI-ToF (MeCN, soft pos. mode): *m/z* calcd for $C_{87}H_{86}N_{13}S$ $[M]^+$ 1344.6845, found 1344.6915.

Elemental analysis for [**L5**]$PF_6 \cdot 2.65H_2O$ (calcd C 67.92, H 5.98, N 11.84; found C 67.52, H 5.55, N 11.74).

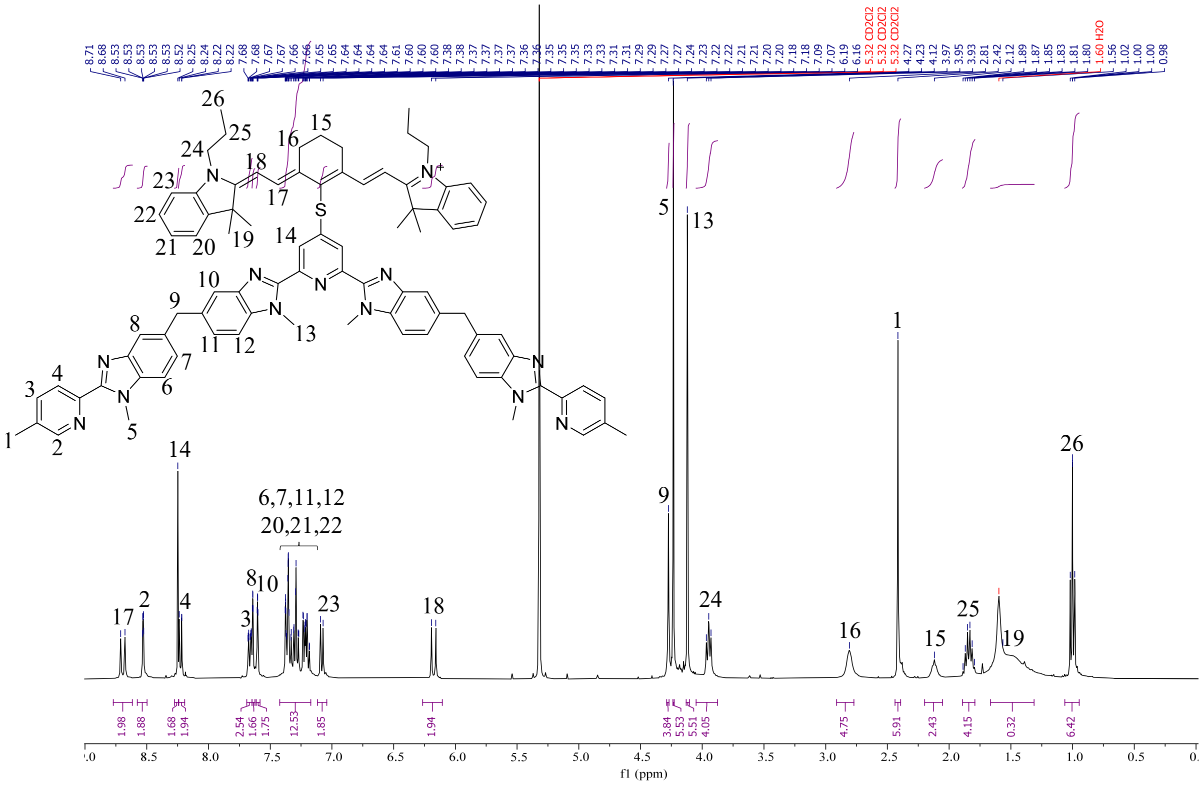

Fig. A2-12 ^{1}H NMR (400 MHz) of [**L5**]$^+$ in CD_2Cl_2.

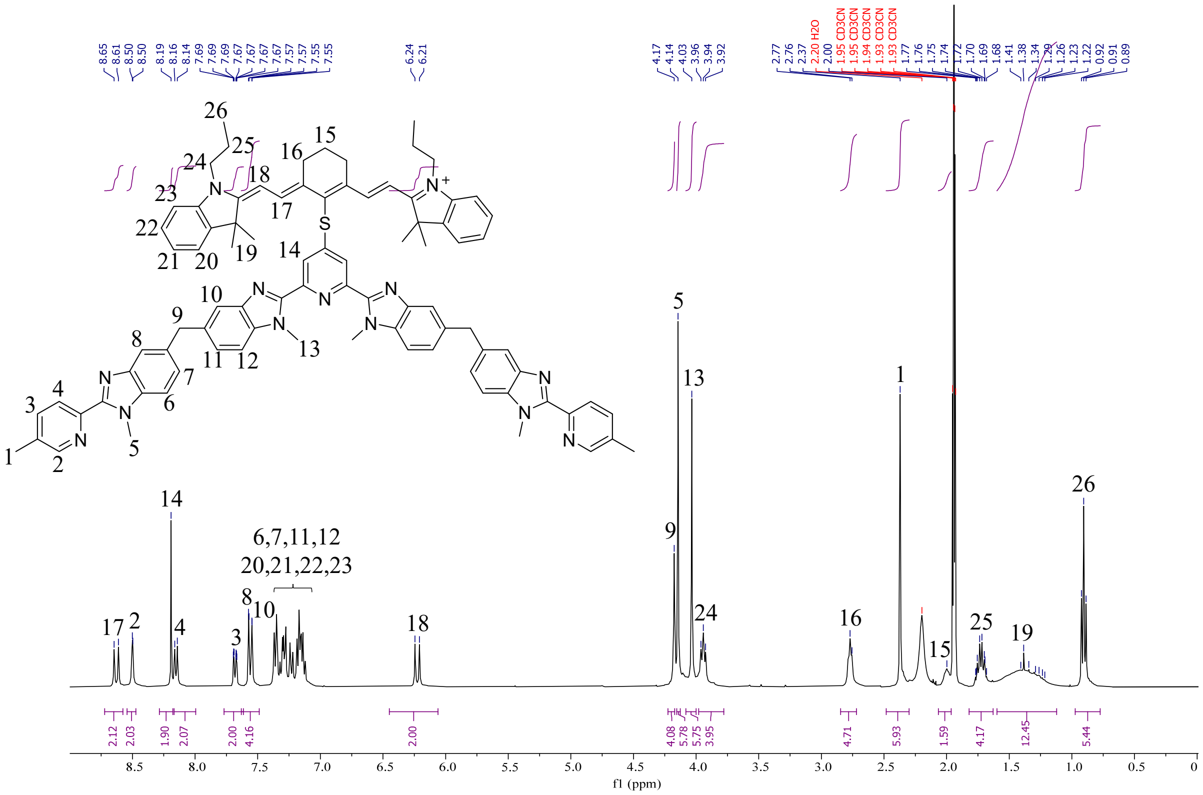

Fig. A2-13 ^{1}H NMR (400 MHz) of [**L5**]$^+$ in CD_3CN.

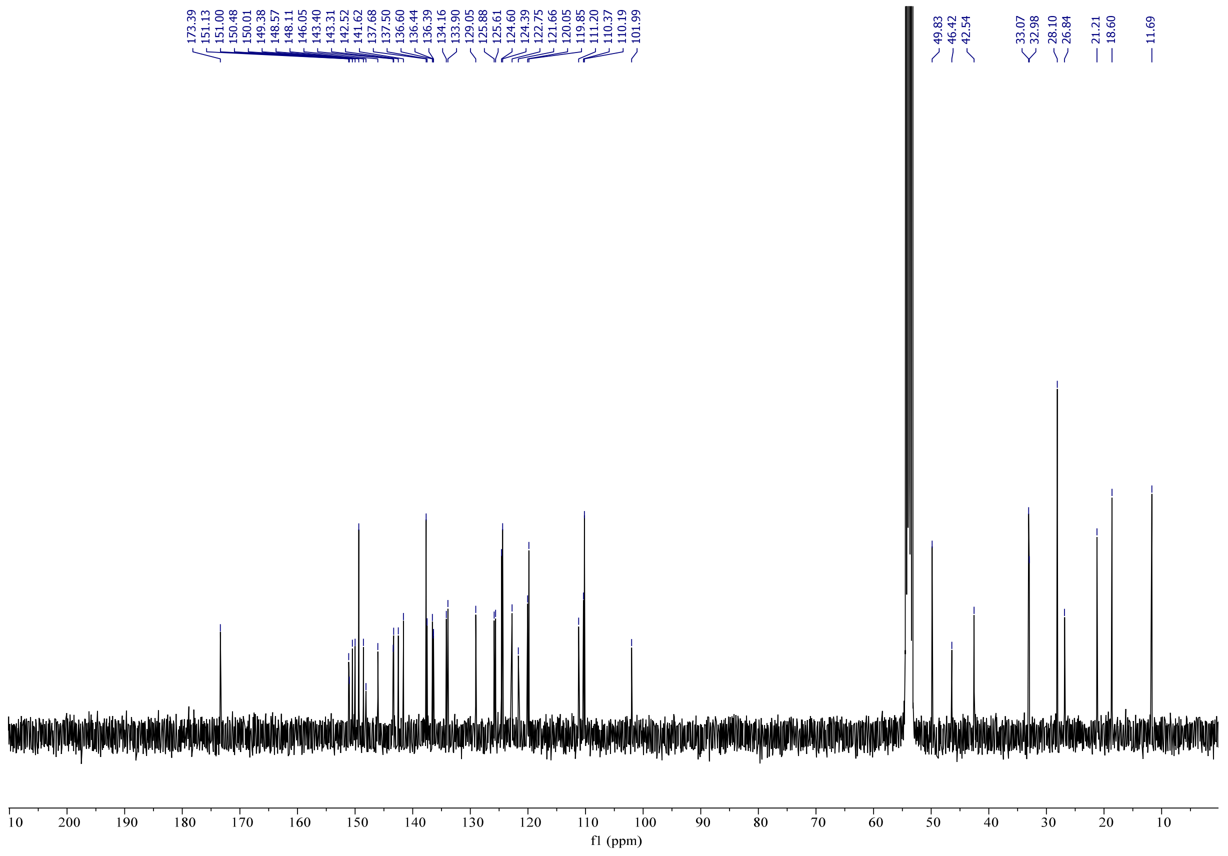

Fig. A2-14 ^{13}C NMR (100 MHz) of [**L5**]$^{+}$ in CD_2Cl_2.

Preparation of helicate [ZnYZn(L5)$_3$](PF$_6$)$_{10}$

To a solution of [**L5**]PF$_6$ (29.2 mg, 18.45 μmol, 3 eq.) in deuterated MeCN (0.4420 g, 489.1 μL) in an NMR tube was added 272.5 mg (319.0 μL, 1 eq.) of a solution of Y(Otf)$_3$ (4.9 mg, 8.77 μmol) in deuterated MeCN (0.4001 g, 474.1 μL) and 206.3 mg (239.2 μL, 2 eq.) of a solution of Zn(Otf)$_2$ (9.0 mg, 24.0 μmol) in deuterated MeCN (0.4106 g, 486.5 μL). The tube was protected from light with aluminum foils and heated to 50 °C in a water bain-marie. The reaction was followed by NMR and after no more changes in the spectra were recorded (around 20 hours), the solution was evaporated to dryness. The residual solid was dissolved in CH_2Cl_2 (5 mL) and a saturated aqueous solution of potassium hexafluorophosphate (5 mL) was added and gently shacked. The organic phase was separated, and the aqueous phase was extracted with CH_2Cl_2 (2x5 mL). The combined organic phases were washed with water (3x5 mL), dried over Na_2SO_4, filtered and evaporated to dryness. The solid was dissolved in a minimum amount of CH_2Cl_2 and the solution was purified by size exclusion chromatography (Bio-Beads S-X1 Support, CH_2Cl_2). The resulting powder was dissolved in a minimum amount of CH_2Cl_2, precipitated by slow addition of pentane and filtered on nylon filter to give [ZnYZn(**L5**)$_3$](PF$_6$)$_{10}$ (21.6 mg, 3.76 μmol, yield: 64%) as a green powder.

^{1}H NMR (400 MHz, CD_3CN) δ 8.30 (d, J = 2.5 Hz, 3H), 8.26 (d, J = 3.2 Hz, 3H), 8.17 (d, J = 8.3 Hz, 6H), 7.90 (dd, J = 8.4, 1.2 Hz, 6H), 7.84 (d, J = 2.0 Hz, 6H), 7.63 (s, 6H), 7.71 – 7.54 (m, 12H), 7.52 – 7.41 (m, 12H), 7.30 (d, J = 8.4 Hz, 6H), 7.16 (d, J = 8.4 Hz, 6H), 6.83 (d, J = 8.4 Hz, 6H), 6.74 (d, J = 8.5 Hz, 6H), 6.46 (d, J = 14.2 Hz, 3H), 6.21 (d, J = 13.9 Hz, 3H), 5.20 (s, 6H), 4.93 (s, 6H), 4.26 – 4.10 (m, 12H), 4.07 (s, 18H), 3.79 (s, 18H), 3.58 (d, J = 16.5 Hz, 6H), 3.09 (d, J = 17.0 Hz, 6H), 2.91 – 2.82 (m, 12H), 2.71 – 2.56 (m, 6H), 2.18 (s, 18H), 1.92 – 1.85 (m, 12H), 1.63 (s, 9H), 1.63 (s, 9H), 1.38 (s, 9H), 1.26 (s, 9H), 1.08 (dt, J = 11.5, 7.3 Hz, 18H).

ESI-MS (MeCN, soft pos. mode): *m/z* calcd for $C_{261}H_{258}F_{42}N_{39}P_7S_3YZn_2$ $[M{\cdot}(PF_6)_7]^{3+}$ 1757.2, found 1756.9; $C_{261}H_{258}F_{36}N_{39}P_6S_3YZn_2$ $[M{\cdot}(PF_6)_6]^{4+}$ 1281.6, found 1281.9; $C_{261}H_{258}F_{30}N_{39}P_5S_3YZn_2$ $[M{\cdot}(PF_6)_5]^{5+}$ 996.1, found 996.4; $C_{261}H_{258}F_{24}N_{39}P_4S_3YZn_2$ $[M{\cdot}(PF_6)_4]^{6+}$ 806.1, found 806.1; $C_{261}H_{258}F_{18}N_{39}P_3S_3YZn_2$ $[M{\cdot}(PF_6)_3]^{7+}$ 670.2, found 670.1.

HR-MS ESI-ToF (MeCN, soft pos. mode): *m/z* calcd for $C_{261}H_{258}F_{42}N_{39}P_7S_3YZn_2$ $[M{\cdot}(PF_6)_7]^{3+}$ 1756.8577, found 1756.8524; $C_{261}H_{258}F_{36}N_{39}P_6S_3YZn_2$ $[M{\cdot}(PF_6)_6]^{4+}$ 1281.6522, found 1281.6520; $C_{261}H_{258}F_{30}N_{39}P_5S_3YZn_2$ $[M{\cdot}(PF_6)_5]^{5+}$ 996.3290, found 996.3219.

Elemental analysis for [ZnYZn(**L5**)$_3$](PF$_6$)$_{10}$·1.35H$_2$O (calcd C 54.70, H 4.59, N 9.53; found C 54.33, H 4.21, N 9.53)

Preparation of helicate $[ZnErZn(L5)_3](PF_6)_{10}$

To a solution of [**L5**]PF_6 (43.9 mg, 27.74 µmol, 3 eq.) in deuterated MeCN (0.4361 g, 516.7 µL) in an NMR tube was added 467.7 mg (545.9 µL, 1 eq.) of a solution of $Er(Otf)_3$ (9.6 mg, 12.42 µmol) in deuterated MeCN (0.6330 g, 750.0 µL) and 0.3036 mg (351.7 µL, 2 eq.) of a solution of $Zn(Otf)_2$ (10.4 mg, 27.74 µmol) in deuterated MeCN (0.4544 g, 538.4 µL). The tube was protected from light with aluminum foils and heated to 50 °C in a water bain-marie. The reaction was followed by NMR and after no more changes in the spectra were recorded (around 20 hours), the solution was evaporated to dryness. The residual solid was dissolved in CH_2Cl_2 (5 mL) and a saturated aqueous solution of potassium hexafluorophosphate (5 mL) was added and gently shaken. The organic phase was separated, and the aqueous phase was extracted with CH_2Cl_2 (2x5 mL). The combined organic phases were washed with water (3x5 mL), dried over Na_2SO_4, filtered and evaporated to dryness. The solid was dissolved in a minimum amount of CH_2Cl_2 and the solution was purified by size exclusion chromatography (Bio-Beads S-X1 Support, CH_2Cl_2). The resulting powder was dissolved in a minimum amount of CH_2Cl_2, precipitated by slow addition of pentane, filtered on nylon filter and dried to dryness under vacuum to give [ZnErZn(**L5**)$_3$]$(PF_6)_{10}$ (37.8 mg, 6.53 µmol, yield: 72%) as a green powder.

ESI-MS (MeCN, soft pos. mode): *m/z* calcd for $C_{261}H_{258}ErF_{42}N_{39}P_7S_3Zn_2$ $[M\cdot(PF_6)_7]^{3+}$ 1783.2, found 1783.0; $C_{261}H_{258}ErF_{36}N_{39}P_6S_3Zn_2$ $[M\cdot(PF_6)_6]^{4+}$ 1301.1, found 1301.8; $C_{261}H_{258}ErF_{30}N_{39}P_5S_3Zn_2$ $[M\cdot(PF_6)_5]^{5+}$ 1011.9, found 1011.4; $C_{261}H_{258}ErF_{24}N_{39}P_4S_3Zn_2$ $[M\cdot(PF_6)_4]^{6+}$ 819.1, found 819.6; $C_{261}H_{258}ErF_{18}N_{39}P_3S_3Zn_2$ $[M\cdot(PF_6)_3]^{7+}$ 681.4, found 681.6, $C_{261}H_{258}ErF_{12}N_{39}P_2S_3Zn_2$ $[M\cdot(PF_6)_2]^{8+}$ 578.1, found 577.5.

HR-MS ESI-ToF (MeCN, soft pos. mode): *m/z* calcd for $C_{261}H_{258}ErF_{42}N_{39}P_7S_3Zn_2$ $[M\cdot(PF_6)_7]^{3+}$ 1783.1997, found 1783.1989; $C_{261}H_{258}ErF_{36}N_{39}P_6S_3Zn_2$ $[M\cdot(PF_6)_6]^{4+}$ 1301.1588, found 1301.1543.

Elemental analysis for [ZnErZn(**L5**)$_3$]$(PF_6)_{10}\cdot 1.65H_2O$ (calcd C 53.91, H 4.53, N 9.39; found C 53.39, H 4.01, N 9.34)

References

A2-1 K. Rurack and M. Spieles, *Anal. Chem.*, 2011, **83**, 1232-1242.

A2-2 G. M. Sheldrick, *Acta Crystallogr. C: Structural Chemistry*, 2015, **71**, 3-8.

A2-3 G. M. Sheldrick, *Acta Crystallogr. A*, 2008, **64**, 112-122.

A2-4 O. V. Dolomanov, L. J. Bourhis, R. J. Gildea, J. A. K. Howard and H. Puschmann, *J. Appl. Crystallogr.*, 2009, **42**, 339-341.

A2-5 L. Farrugia, *J. Appl. Crystallogr.*, 1997, **30**, 565.

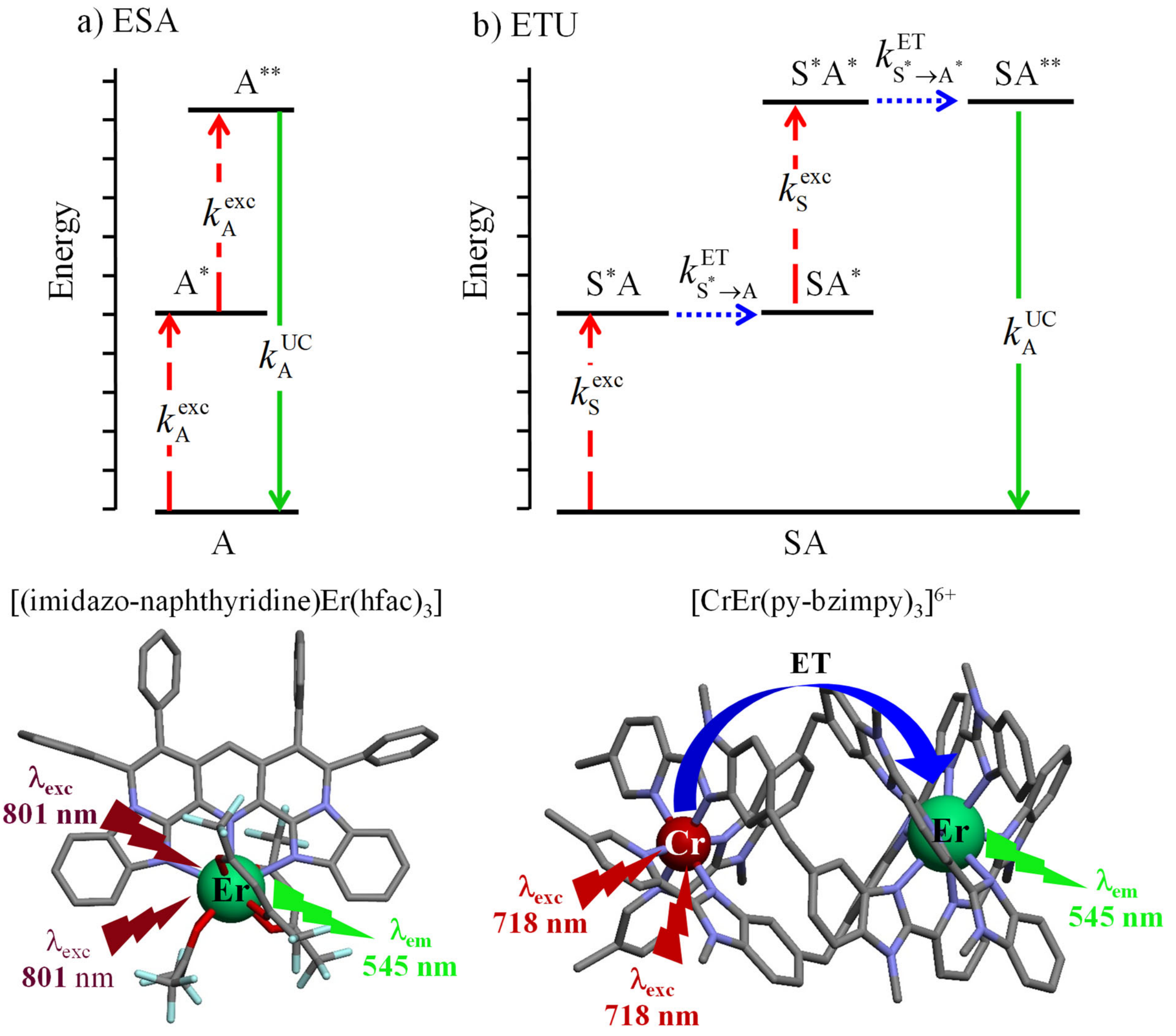

$\phi^{UC} = 1.0(1)\cdot 10^{-6}$ (P = 25 W/cm², CH_2Cl_2, 298 K)

$\phi^{UC} = 3.5(4)\cdot 10^{-8}$ (P = 25 W/cm², CH_3CN, 298 K)

Fig. S1 Light upconversion (UC) mechanisms using linear optics and electronic transitions of single-operator nature *via* a) excited state absorption (ESA) and b) energy transfer upconversion (ETU) in single molecule (S = sensitizer, A = activator). The molecular structures of [(imidazo-naphthyridine)Er(hfac)3] (ESA, A = Er^{3+}; CCDC 2201191)[24] and $[CrEr(py\text{-}bzimpy)_3]^{6+}$ (ETU, S = Cr^{3+}, A = Er^{3+}; CCDC 1003568)[23,27] with UC quantum yields determined in non-deuterated solvents at room temperature are shown as illustrations. Color codes : C = grey, N = dark blue, F = light blue).

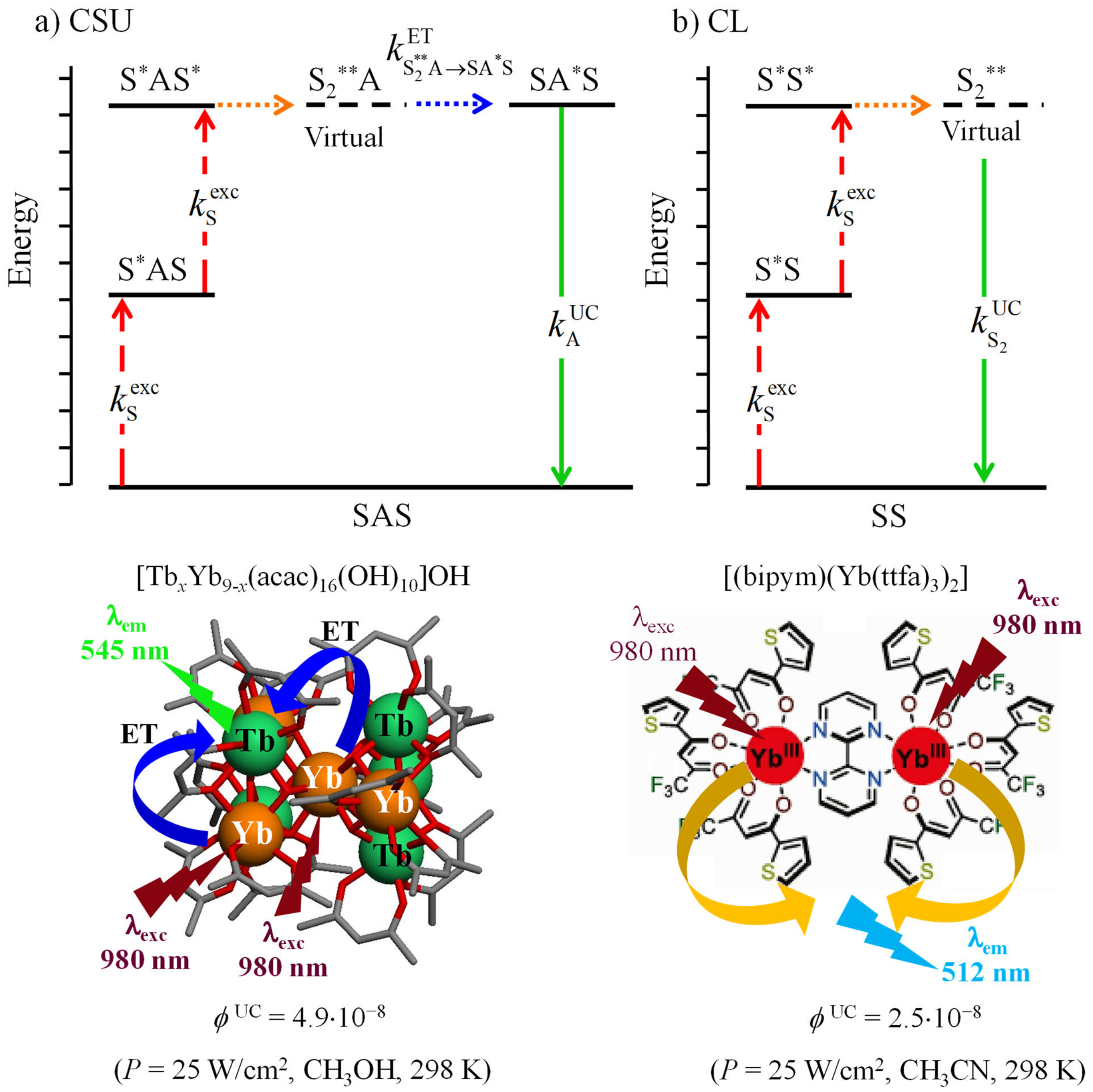

Fig. S2 Light upconversion (UC) mechanisms using linear optics and electronic transitions of double-operator nature *via* a) cooperative sensitization upconversion (CSU) and b) cooperative luminescence (CL) in single molecule (S = sensitizer = Yb, A = activator = Tb). The molecular structures of $[Tb_xYb_{9-x}(acac)_{16}(OH)_{10}]OH$ (CSU, S = Yb^{3+}, A = Tb^{3+}; CCDC 711970)[31] and of $[(bipym)(Yb(ttfa)_3)_2]$ (CL, S = Yb^{3+})[37] with associated UC quantum yields determined in non-deuterated solvent at room temperature are shown as illustrations. Color codes : C = grey, N = blue. O = red.

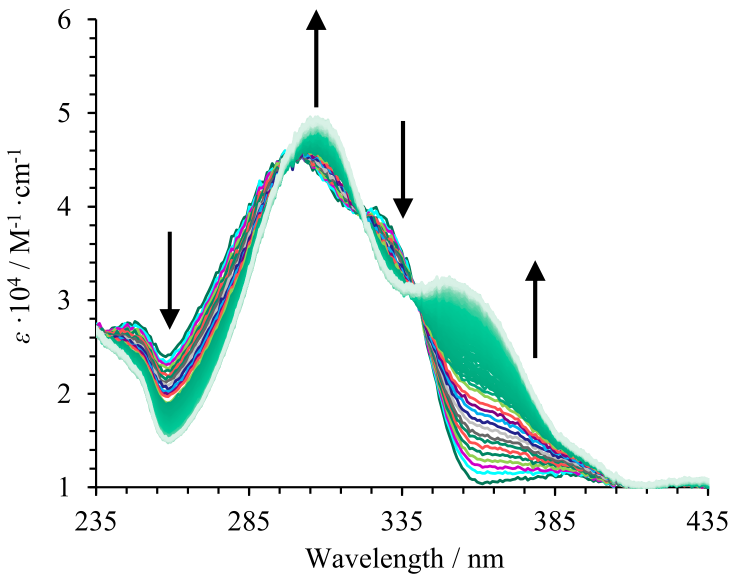

Fig. S3 Spectrophotometric titration of a solution of [**L3**]$^+$ at $2{\cdot}10^{-5}$ M with $Eu(CF_3SO_3)_3$ at $1.1{\cdot}10^{-4}$ M. The changes in absorbance due to complexation are evidenced. Measurements were conducted in dry acetonitrile at 293 K.

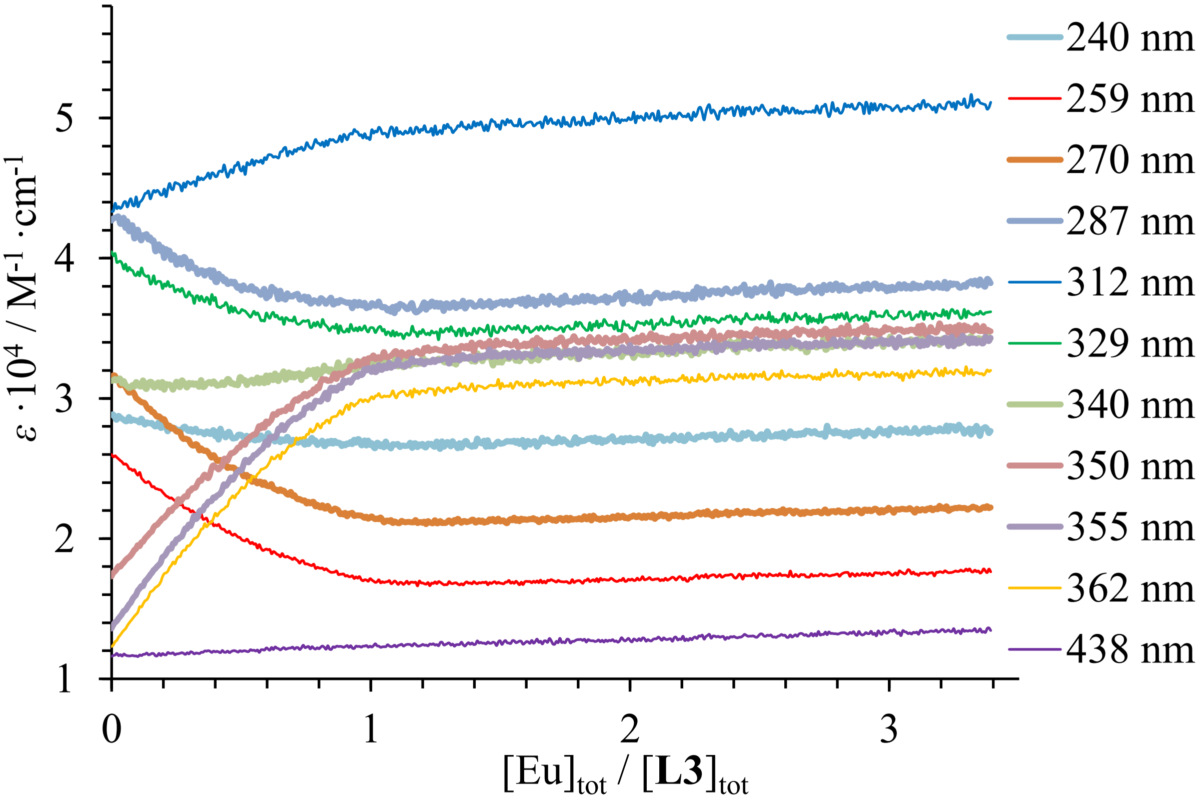

Fig. S4 Variation of molar extinction at different wavelengths observed for the spectrophotometric titration of [**L3**]$^+$ with $Eu(CF_3SO_3)_3$ (total ligand concentration: $2{\cdot}10^{-5}$ M in acetonitrile, 293 K).

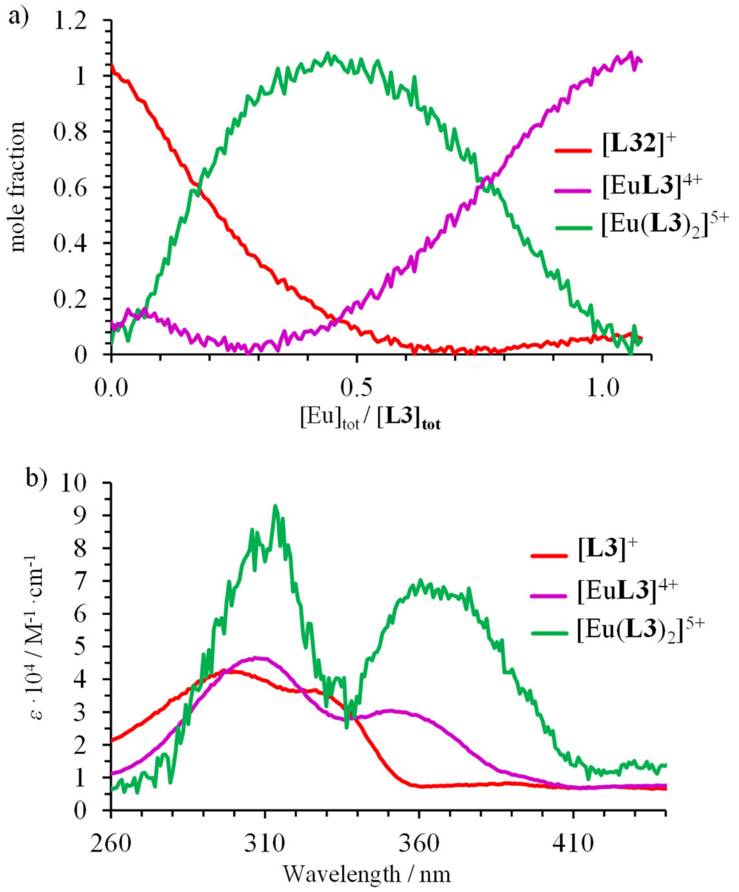

Fig. S5 a) Evolving factor analysis using three absorbing eigenvectors and b) re-constructed individual electronic absorption spectra.[73–75]

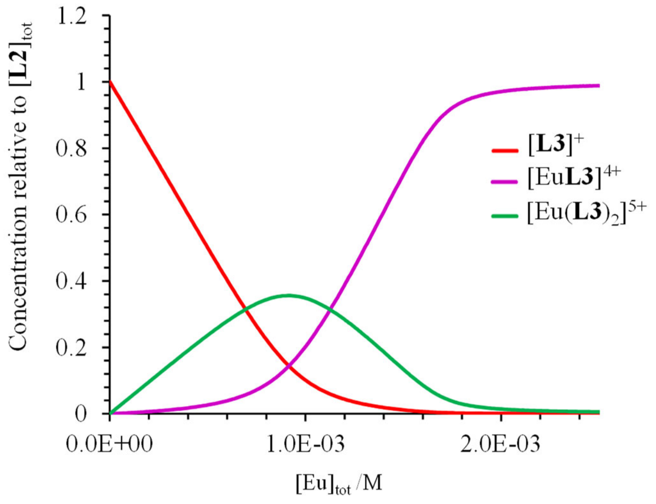

Fig. S6 Concentration profiles from a HySS2009 (L. Alderighi, P. Gans, A. Ienco, D. Peters, A. Sabatini and A. Vacca, *Coord. Chem. Rev.*, 1999, **184**, 311–318) simulation of the complex species $[Eu(\mathbf{L3})_n]^{(3+n)+}$ fort a total ligand concentration of $2 \cdot 10^{-3}$ M.

Table S1. Crystal data and structure refinement for [**L5**]$PF_6 \cdot C_3H_5N \cdot 0.25(C_5H_{12}O)$.

Compound	[**L5**]$PF_6 \cdot C_3H_5N \cdot 0.25(C_5H_{12}O)$	CCDC 2376478
Empirical formula	$C_{91.25}H_{94}F_6N_{14}O_{0.25}PS$	
Chemical formula moiety	$C_{87}H_{86}N_{13}S$, F_6P, $0.75(C_3H_5N)$, $0.25(C_3H_5N)$, $0.25(C_5H_{12}O)$	
Formula weight	1567.83	
Temperature	99.98(10) K	
Wavelength	1.54184 Å	
Crystal system	Triclinic	
Space group	P -1	
Unit cell dimensions	*a* = 14.55269(18) Å	α= 65.7564(12)°.
	b = 17.0818(3) Å	β= 69.7158(11)°.
	c = 20.3355(2) Å	γ = 74.9477(12)°.
Volume	4282.57(11) $Å^3$	
Z	2	
Density (calculated)	1.216 Mg/m^3	
Absorption coefficient	1.055 mm^{-1}	
F(000)	1653	
Crystal size	0.27 x 0.15 x 0.05 mm^3	
Theta range for data collection	2.477 to 76.236°.	
Index ranges	−18<=*h*<=18, −21<=*k*<=21, -25<=*l*<=16	
Reflections collected	70432	
Independent reflections	17309 [*R*(int) = 0.0195]	
Completeness to theta = 67.684°	99.8 %	
Absorption correction	Analytical	
Max. and min. transmission	0.946 and 0.818	
Refinement method	Full-matrix least-squares on F^2	
Data / restraints / parameters	17309 / 315 / 1178	
Goodness-of-fit on F^2	1.023	
Final *R* indices [*I*>2sigma(*I*)]	*R*1 = 0.0979, w*R*2 = 0.2832	
R indices (all data)	*R*1 = 0.1079, w*R*2 = 0.2940	
Extinction coefficient	n/a	
Largest diff. peak and hole	1.317 and −0.643 e.$Å^{-3}$	

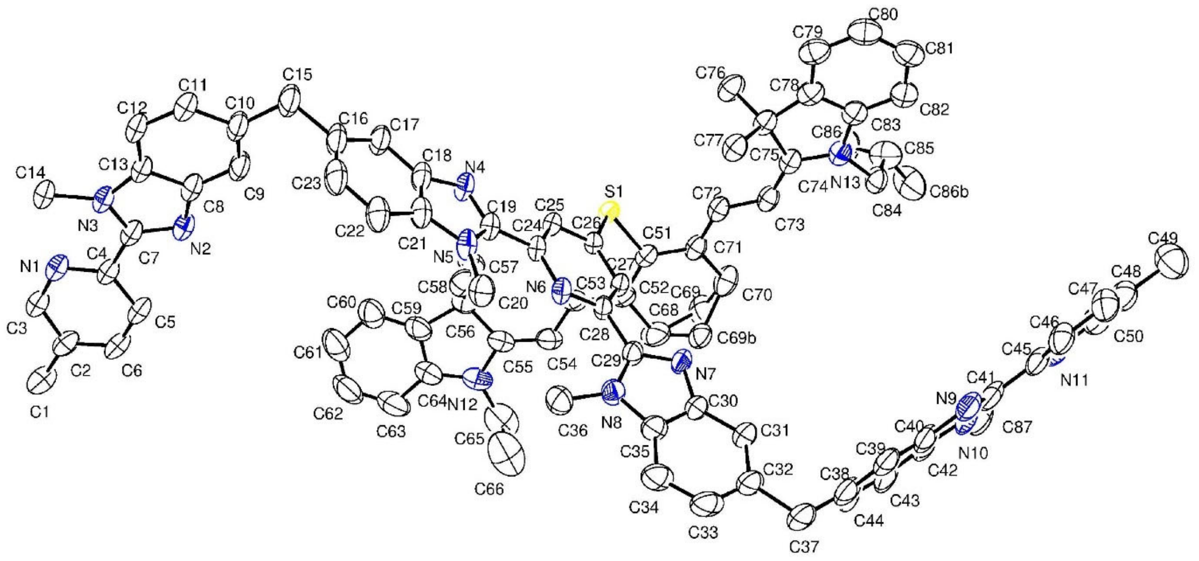

Fig. S7 ORTEP view of $C_{87}H_{86}N_{13}S$ (thermal ellipsoids are drawn at 40% probability) with numbering scheme.

Table S2 Bond lengths [Å] and angles [°] for the sulphur in [**L5**]PF_6·C_3H_5N·0.25($C_5H_{12}O$).

Atom	Atom	Length /Å	Atom	Atom	Atom	Angle /°
S(1)	C(26)	1.769(3)	C(26)	S(1)	C(51)	102.14(13)
S(1)	C(51)	1.794(3)				

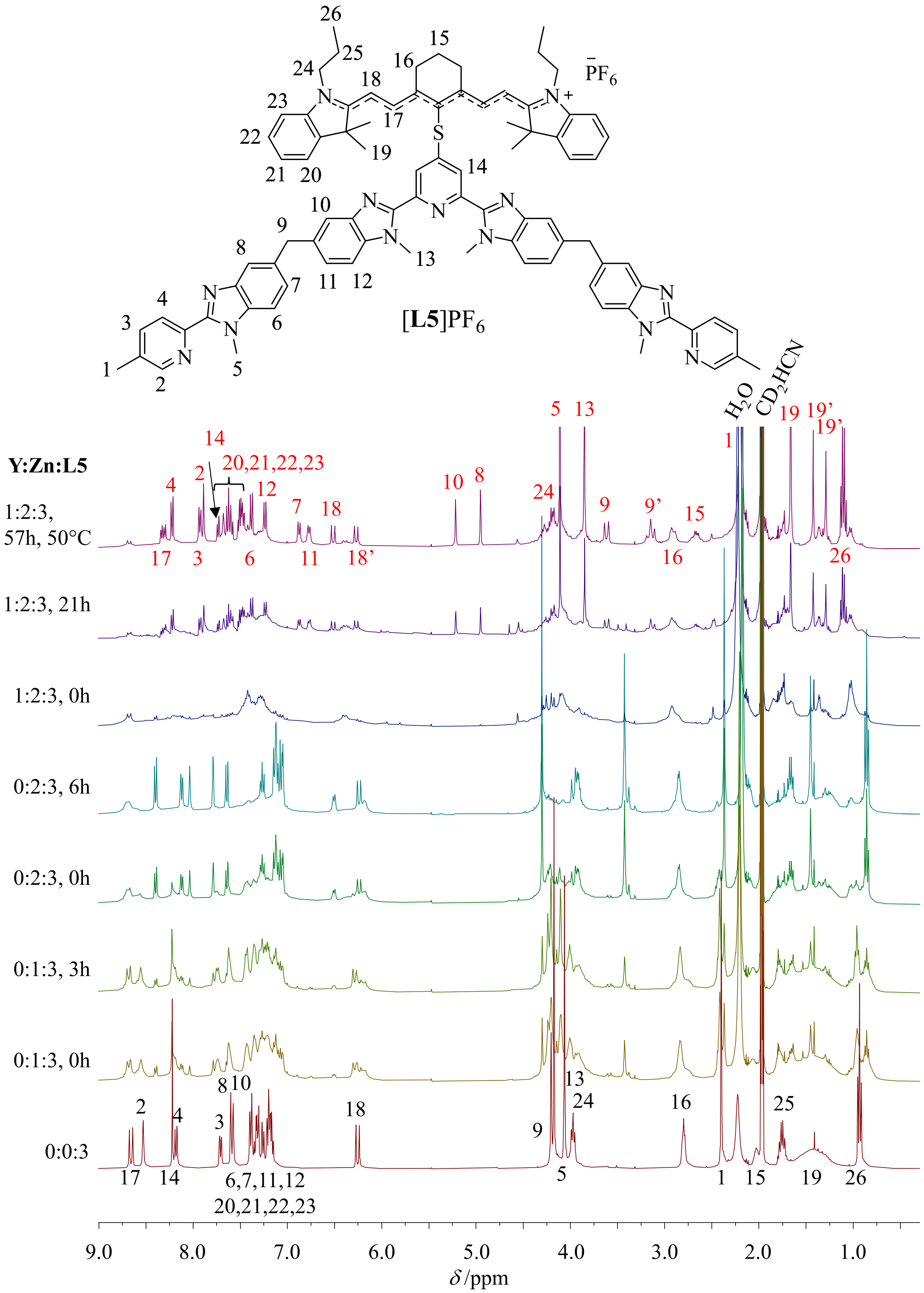

Fig. S8 ^{1}H NMR titration of [**L5**]PF_6 by $Zn(CF_3SO_3)_2$ and $Y(CF_3SO_3)_3$ to give $1.54 \cdot 10^{-3}$ M of [ZnYZn(**L5**)$_3$]$(PF_6)_3(CF_3SO_3)_7$ (CD_3CN, 298 K).

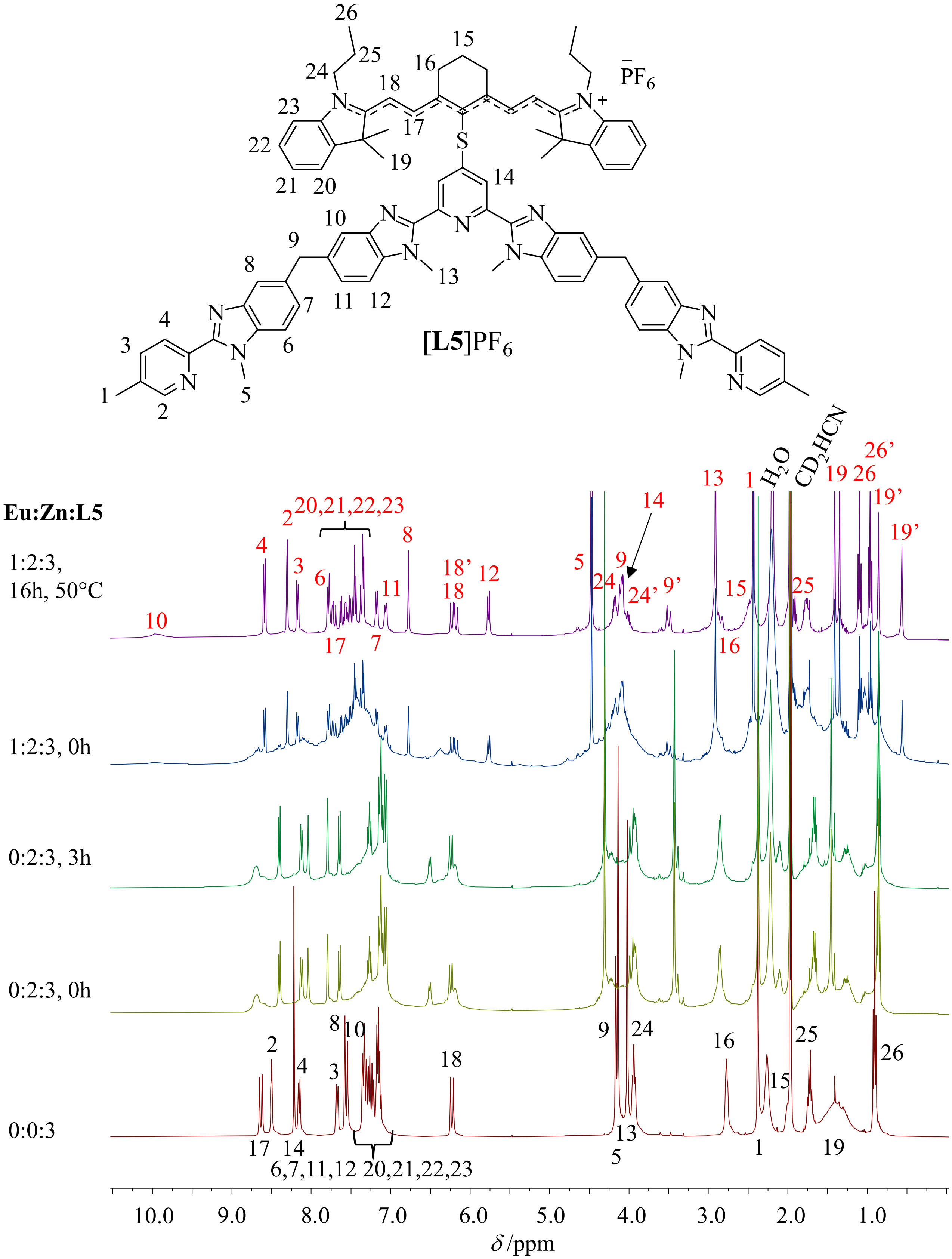

Fig. S9 ^{1}H NMR titration of [**L5**]PF_6 by $Zn(CF_3SO_3)_2$ and $Eu(CF_3SO_3)_3$ to give $3.93 \cdot 10^{-3}$ M of [ZnEuZn(**L5**)$_3$]$(PF_6)_3(CF_3SO_3)_7$ (CD_3CN, 298 K).

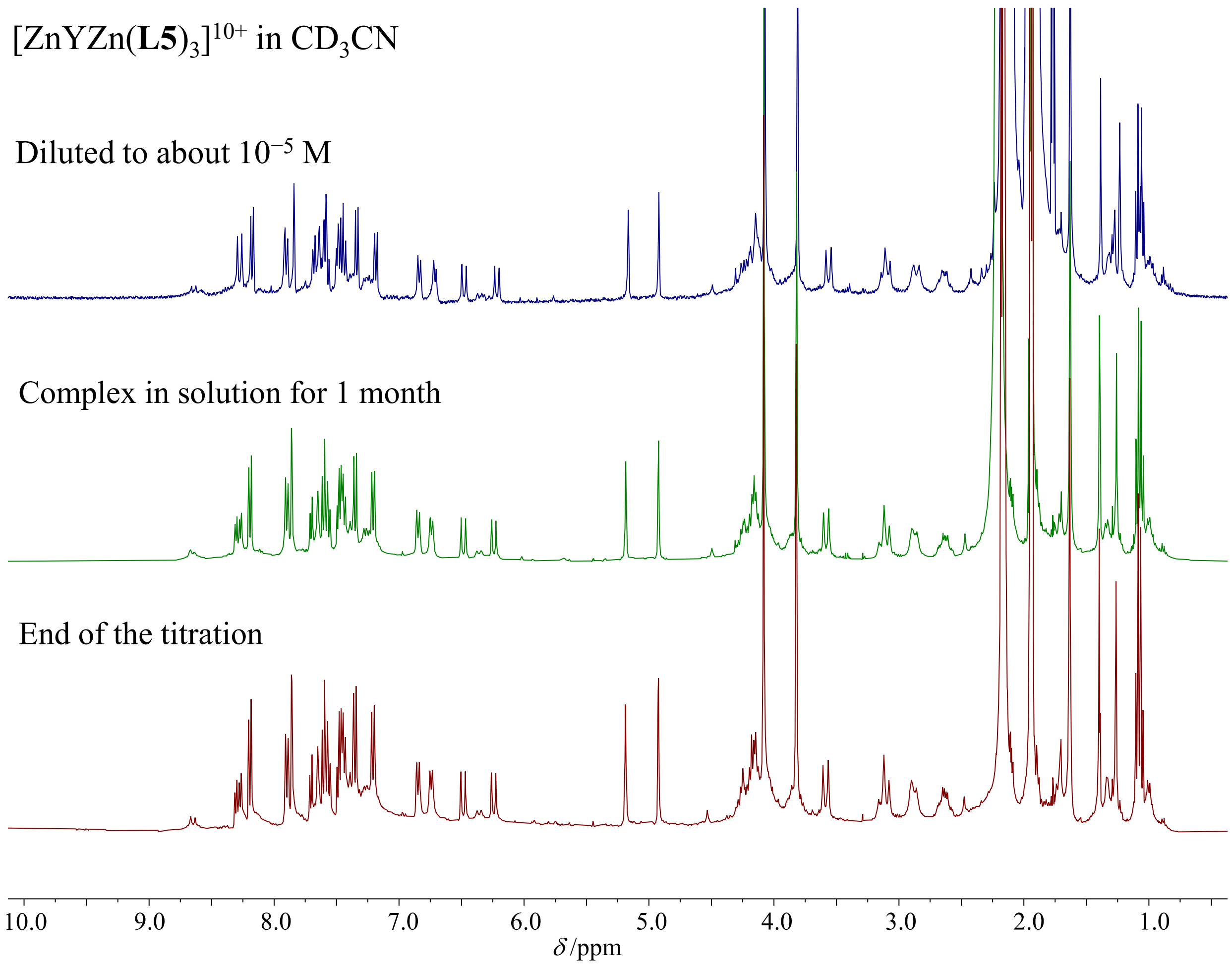

Fig. S10 ^{1}H NMR (400 MHz) spectra of $[ZnYZn(\mathbf{L5})_3]^{10+}$ in MeCN-d_3. In red the freshly prepared helicate complex, in green the same solution let in the same tube for 1 month and in blue the same solution diluted to $3.4\cdot10^{-5}$ M.

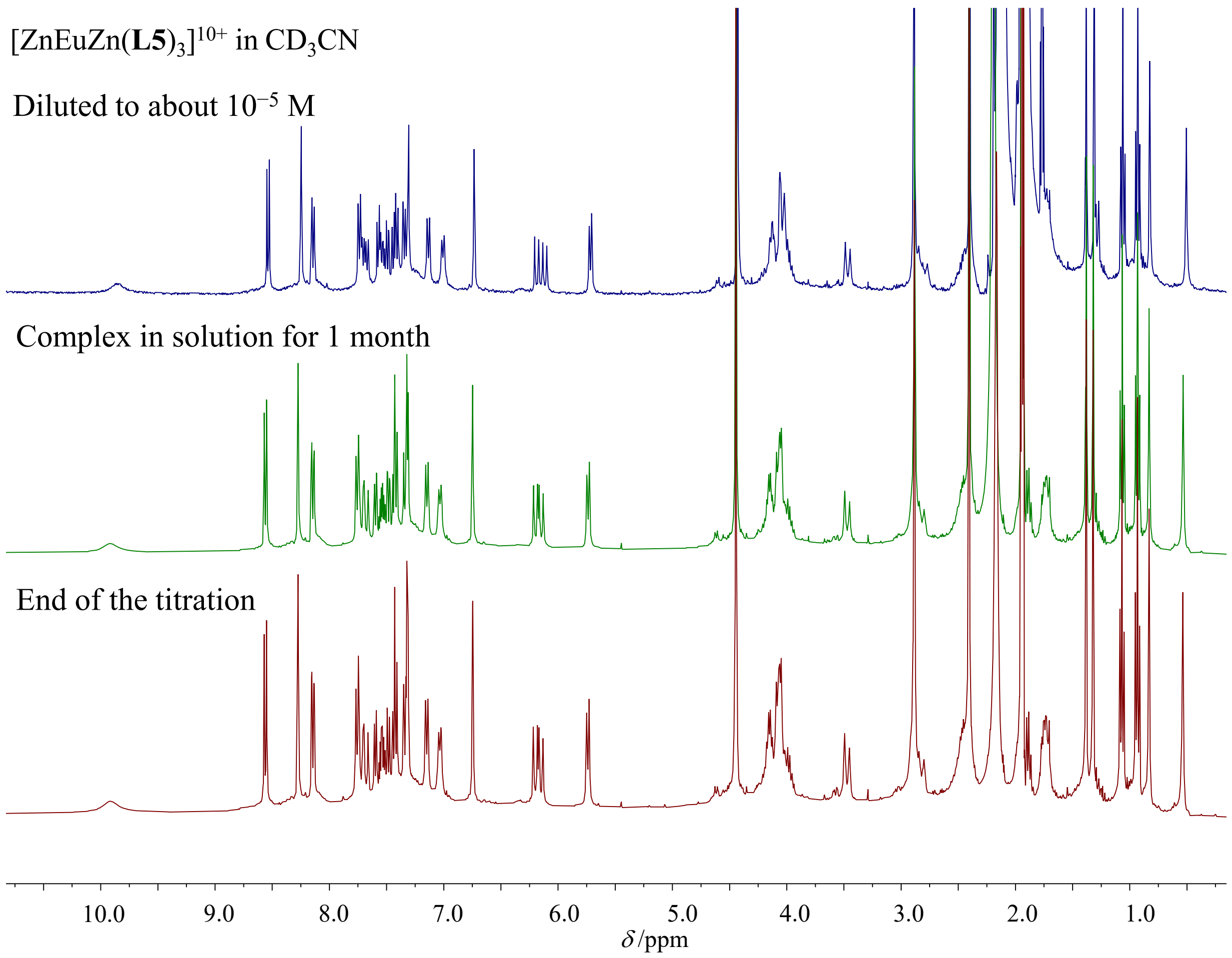

Fig. S11 ^{1}H NMR (400 MHz) spectra of $[ZnEuZn(\mathbf{L5})_3]^{10+}$ in MeCN-d_3. In red the freshly prepared helicate complex, in green the same solution let in the tube for 1 month and in blue the same solution diluted to $8.3 \cdot 10^{-5}$ M.

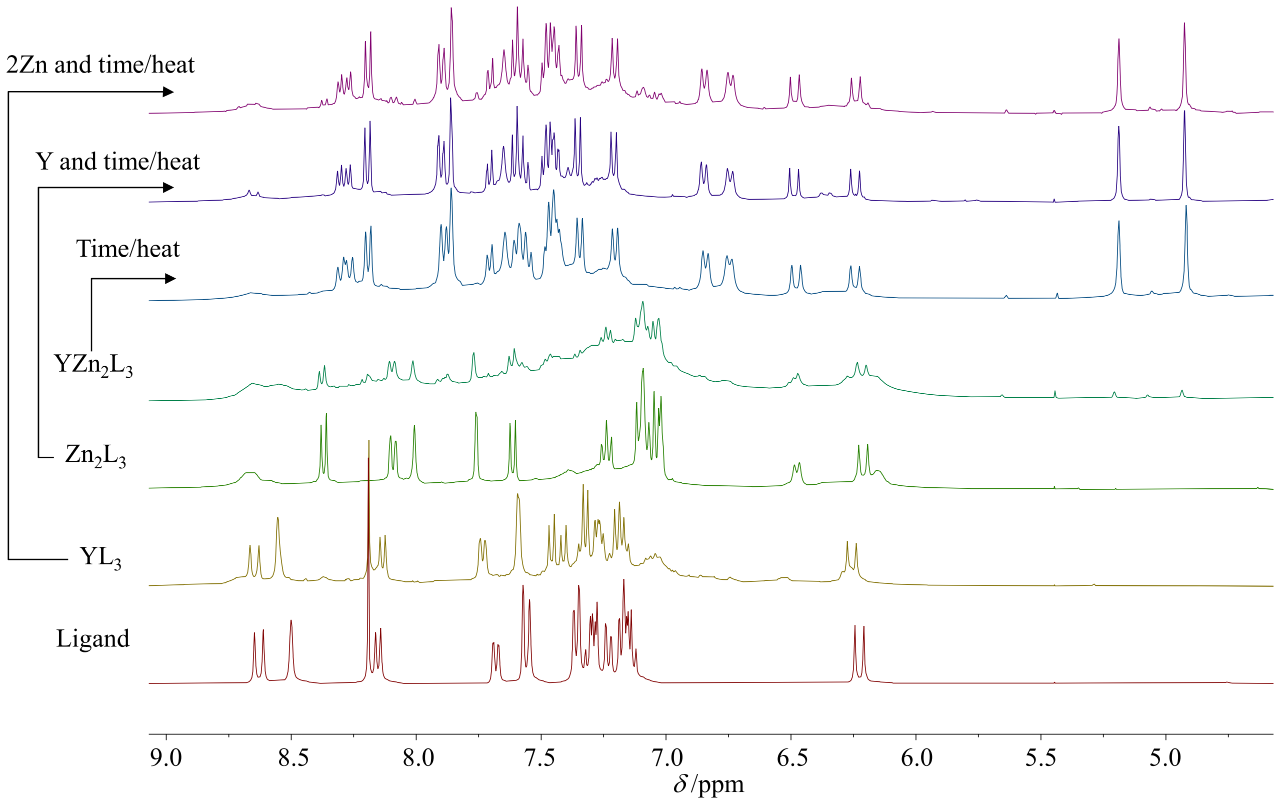

Fig. S12 Summary of the addition of the metal highlighting the unimportance of the metal addition's order in the formation of $[ZnYZn(\mathbf{L5})_3]^{10+}$ complex.

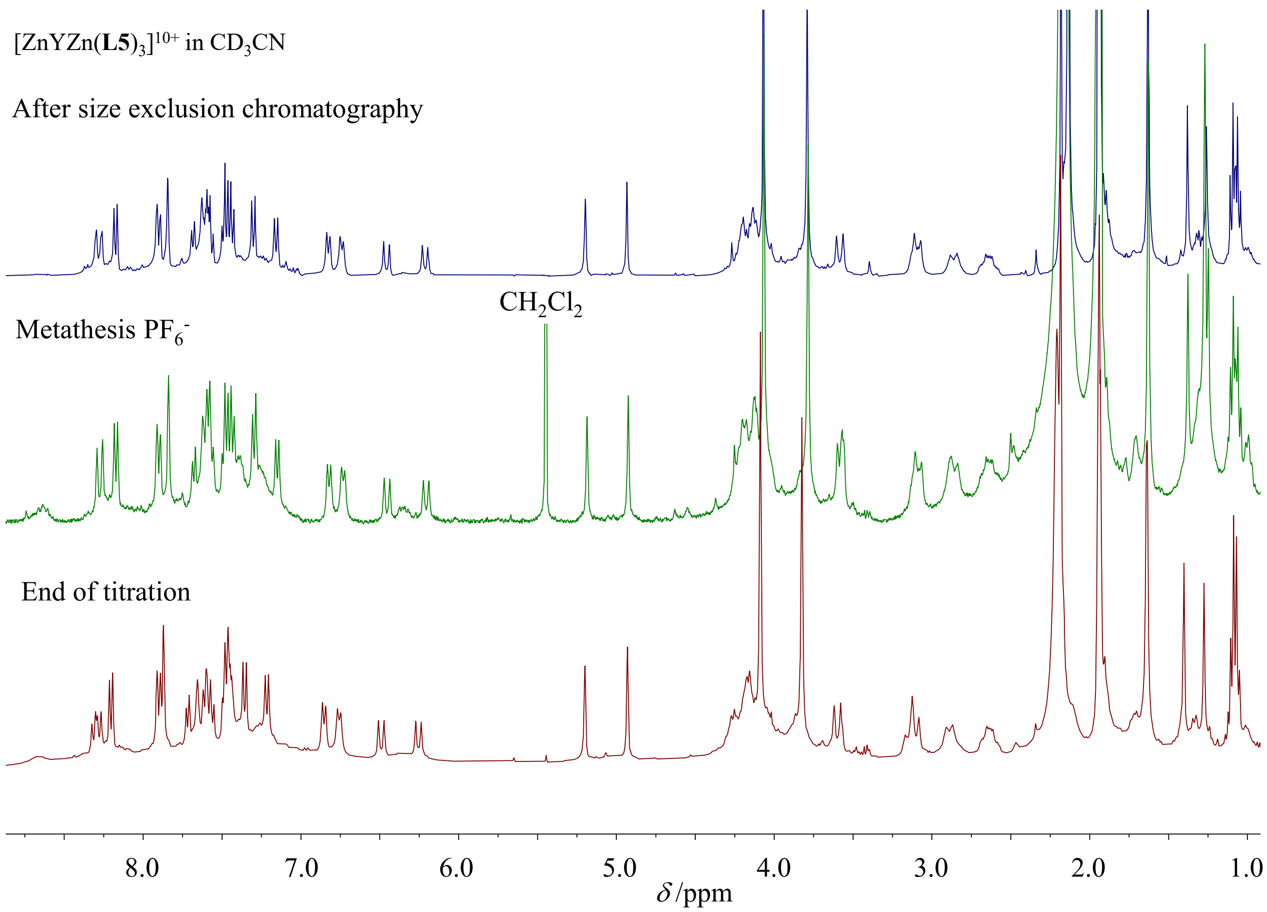

Fig. S13 ^{1}H NMR (400 MHz) spectra of [ZnYZn(**L5**)$_3$]$^{10+}$ in MeCN-d^3. In red the freshly prepared helicate complex, in green the complex after complete metathesis to PF_6, and in blue the complex after size exclusion chromatography purification.

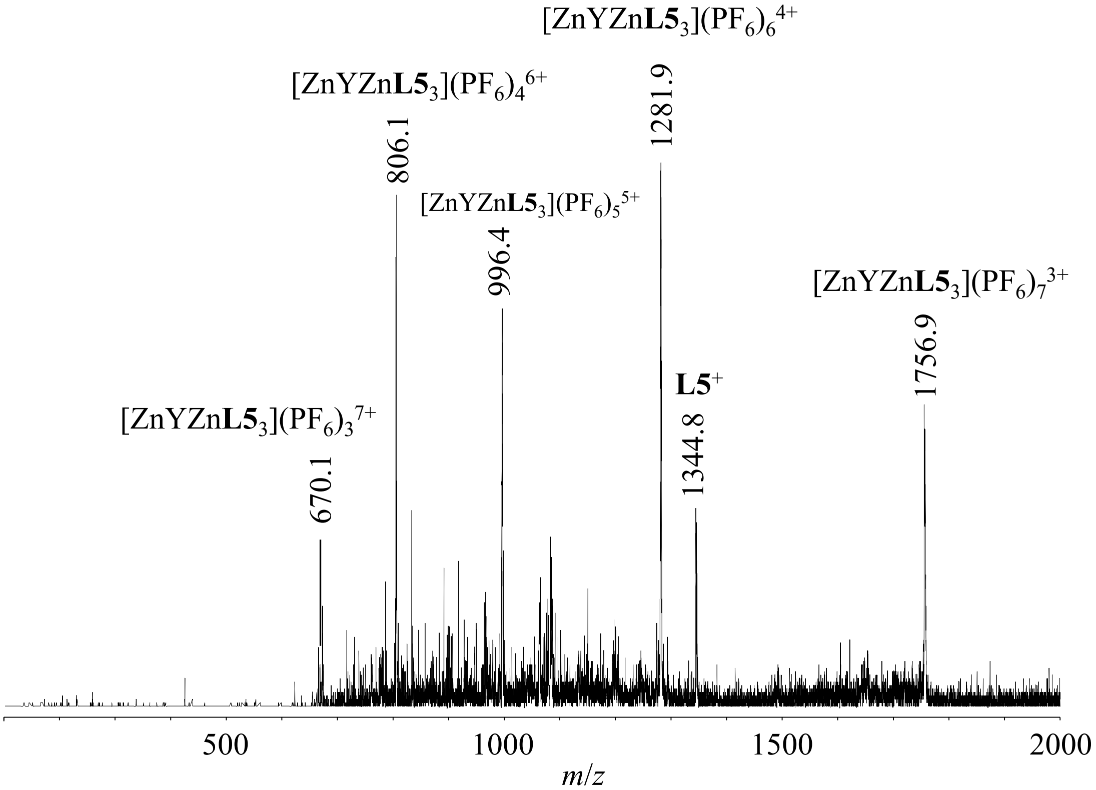

Fig. S14 ESI-MS spectrum of [ZnYZn(**L5**)$_3$](PF$_6$)$_{10}$ at $3\cdot10^{-5}$ M in MeCN.

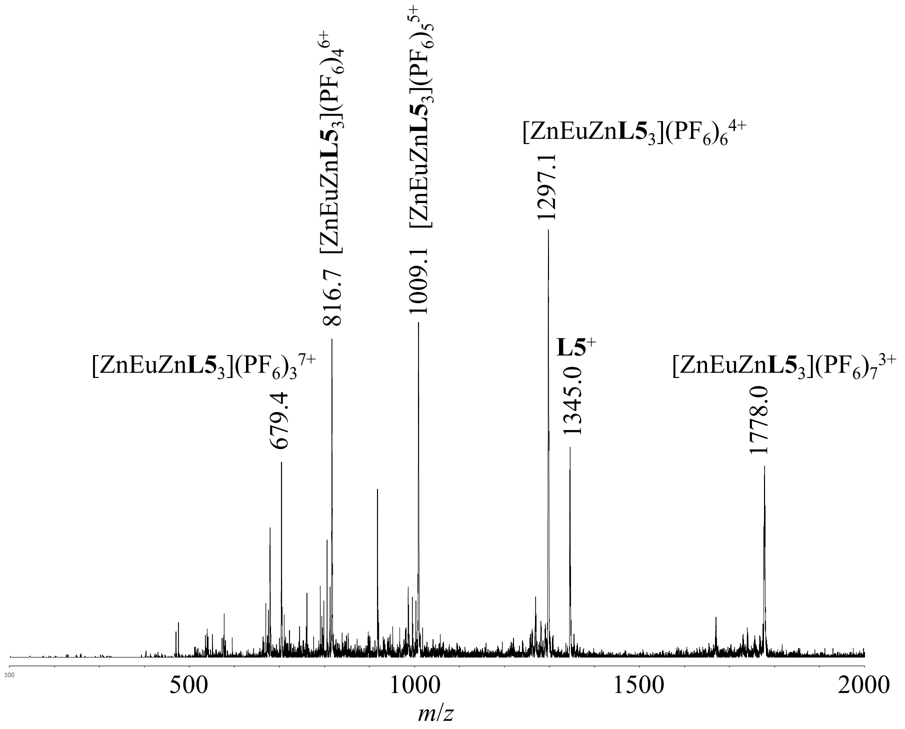

Fig. S15 ESI-MS spectrum of [ZnEuZn(**L5**)$_3$](PF$_6$)$_{10}$ at $5\cdot10^{-5}$ M in MeCN.

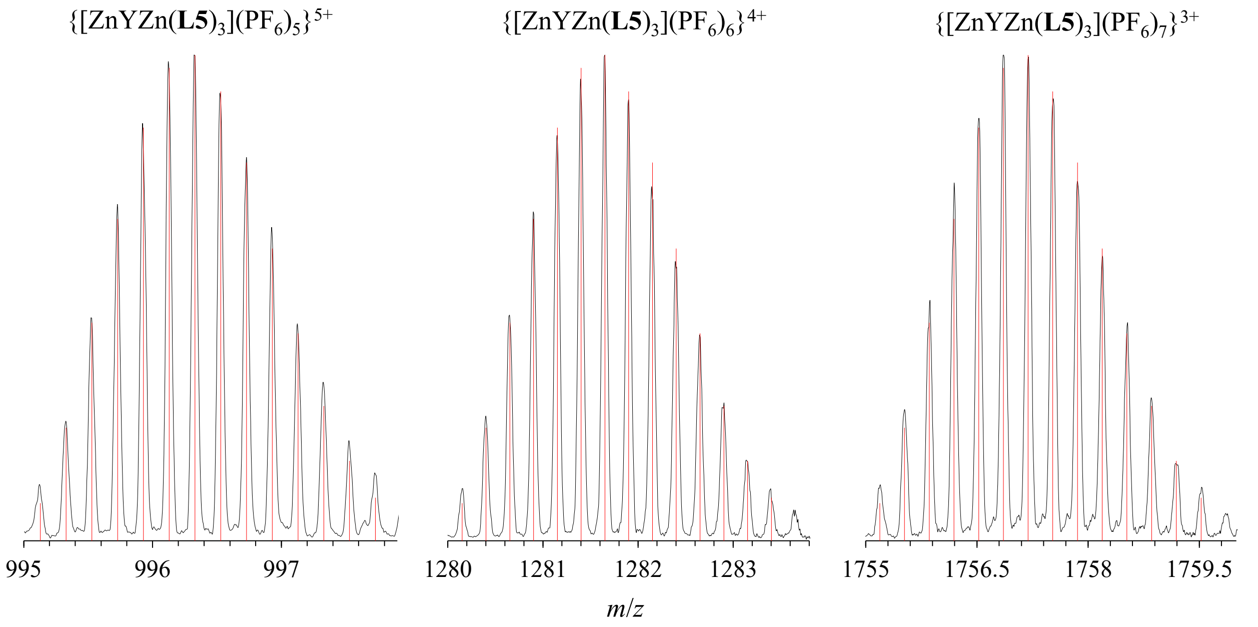

Fig. S16 Zoomed HR-MS ESI-ToF spectrum of [ZnYZn(**L5**)$_3$](PF$_6$)$_{10}$ at $3\cdot10^{-5}$ M in MeCN in the range of the complexes of interest. In black the experimental data and in red the isotopic distribution of the corresponding molecular formula of the complexes.

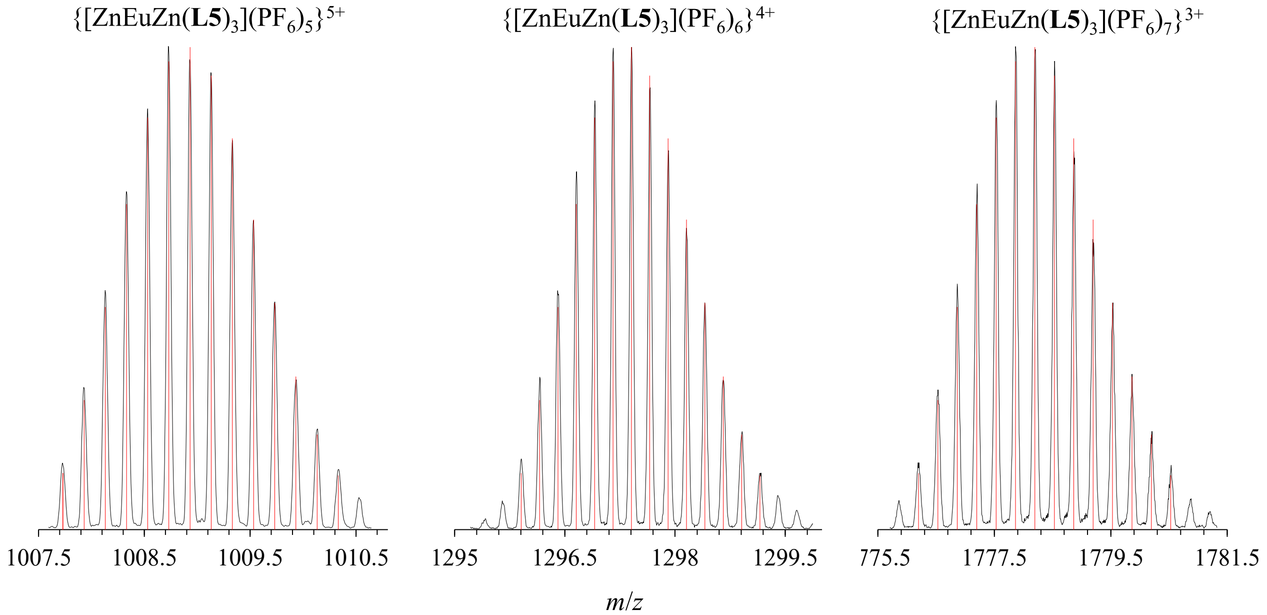

Fig. S17 Zoomed HR-MS ESI-ToF spectrum of [ZnEuZn**L5**$_3$](PF$_6$)$_{10}$ at $5\cdot10^{-5}$ M in MeCN in the range of the complexes of interest. In black the experimental data and in red the isotopic distribution of the corresponding molecular formula of the complexes.

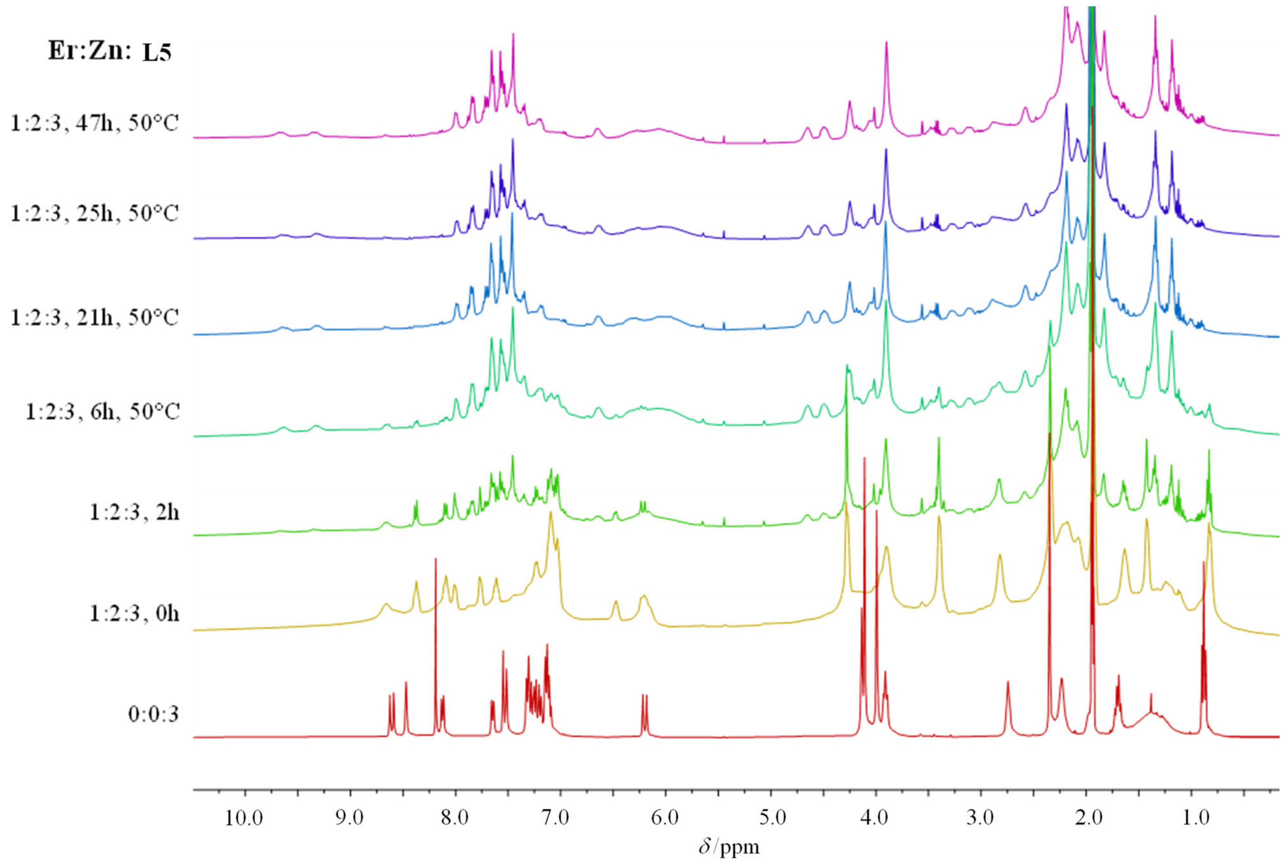

Fig. S18 ^{1}H NMR titration of [**L5**]$^{+}$ by Zn^{2+} and Er^{3+} in CD_3CN at $6.39\cdot10^{-3}$ M of [ZnErZn(**L5**)$_3$]$(PF_6)_3(CF_3SO_3)_7$ (CD_3CN, 298 K).

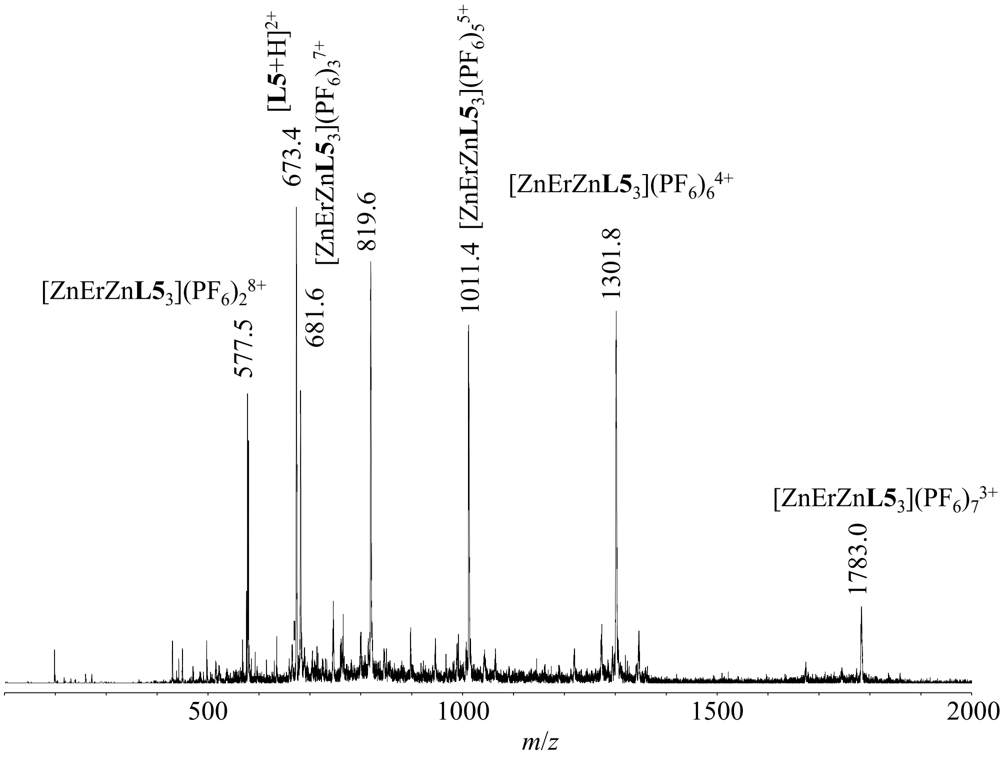

Fig. S19 ESI-MS spectrum of [ZnErZn**L5**$_3$]$(PF_6)_{10}$ at $9\cdot10^{-5}$ M in MeCN.

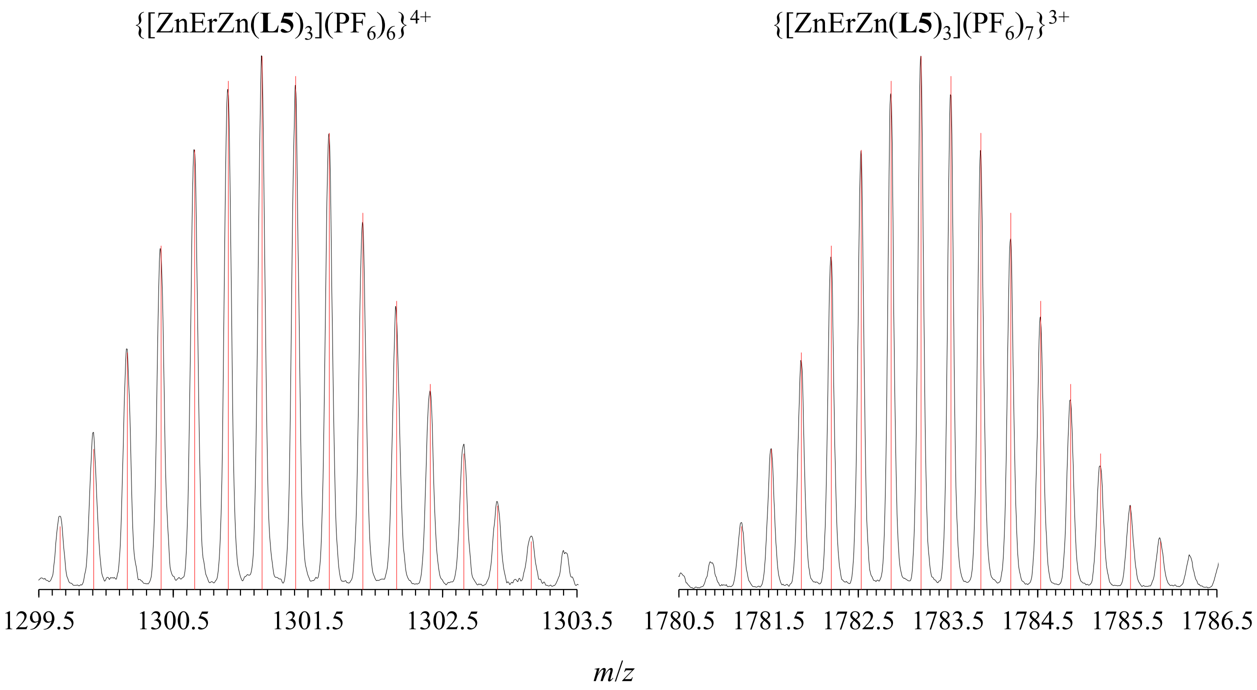

Fig. S20 HR-MS ESI-ToF spectrum of [ZnErZn**L5**$_3$](PF_6)$_{10}$ at $9\cdot10^{-5}$ M in MeCN. In black the experimental data and in red the isotopic distribution of the corresponding molecular formula of the complexes.

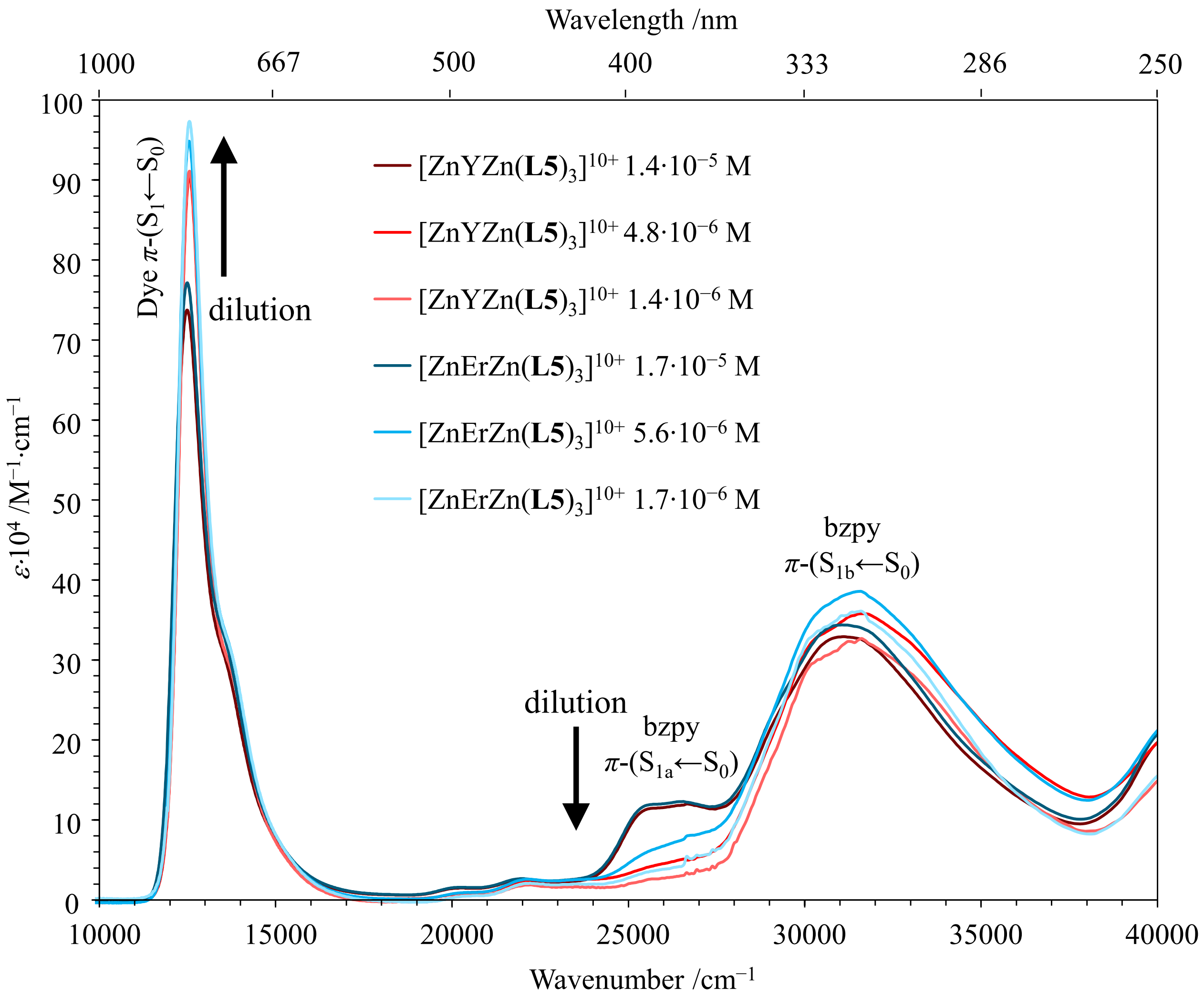

Fig. S21 Absorption spectra of $[ZnYZn(\mathbf{L5})_3]^{10+}$ and $[ZnErZn(\mathbf{L5})_3]^{10+}$ complexes recorded in acetonitrile solutions at 293 K at different concentrations showing the effect of dilution.

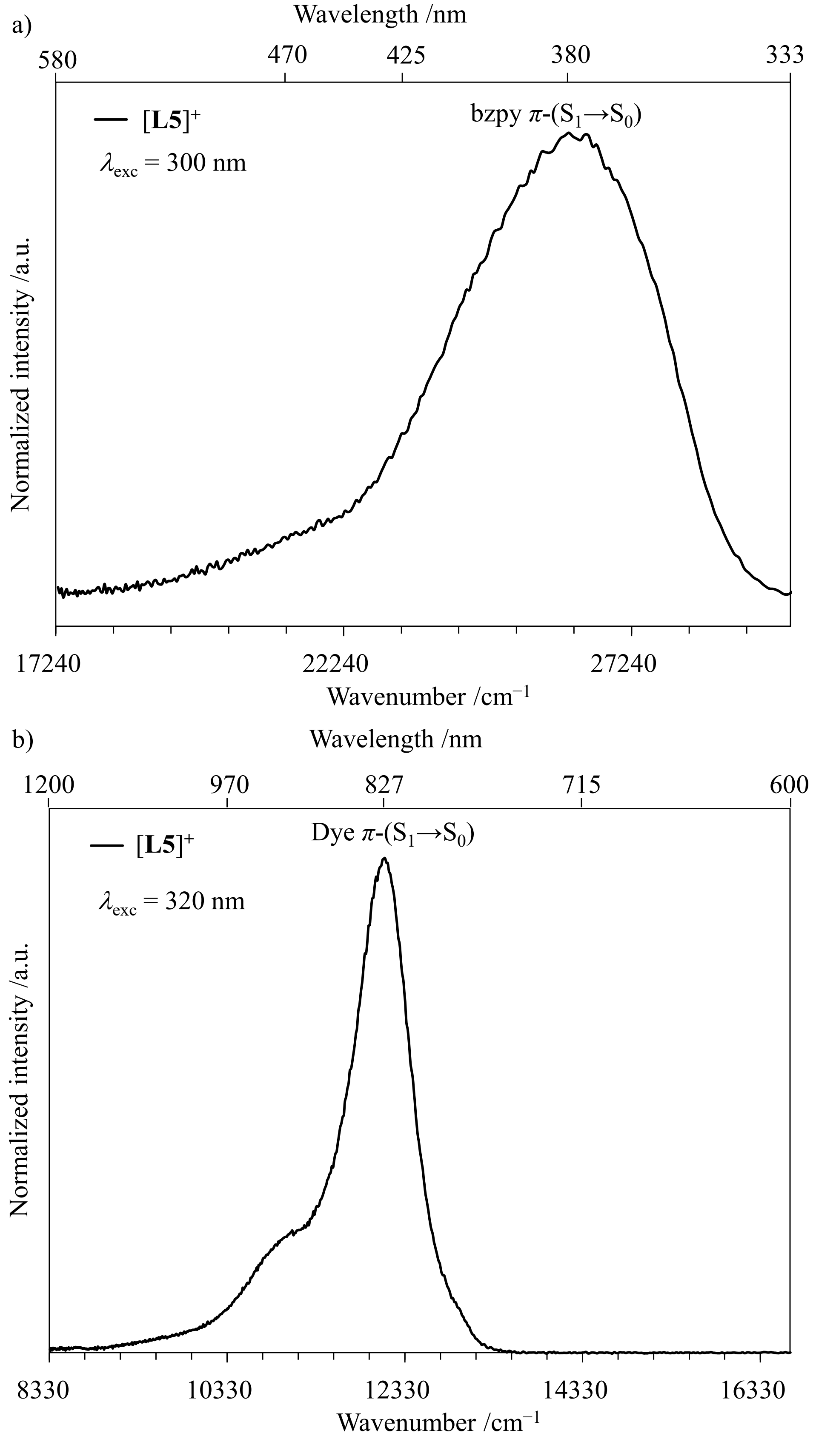

Fig. S22 Emission spectra of [**L5**]$^{+}$ at $2{\cdot}10^{-6}$ M (CH_3CN, 293 K) upon lamp excitation a) λ_{exc} = 300 nm and b) λ_{exc} = 320 nm.

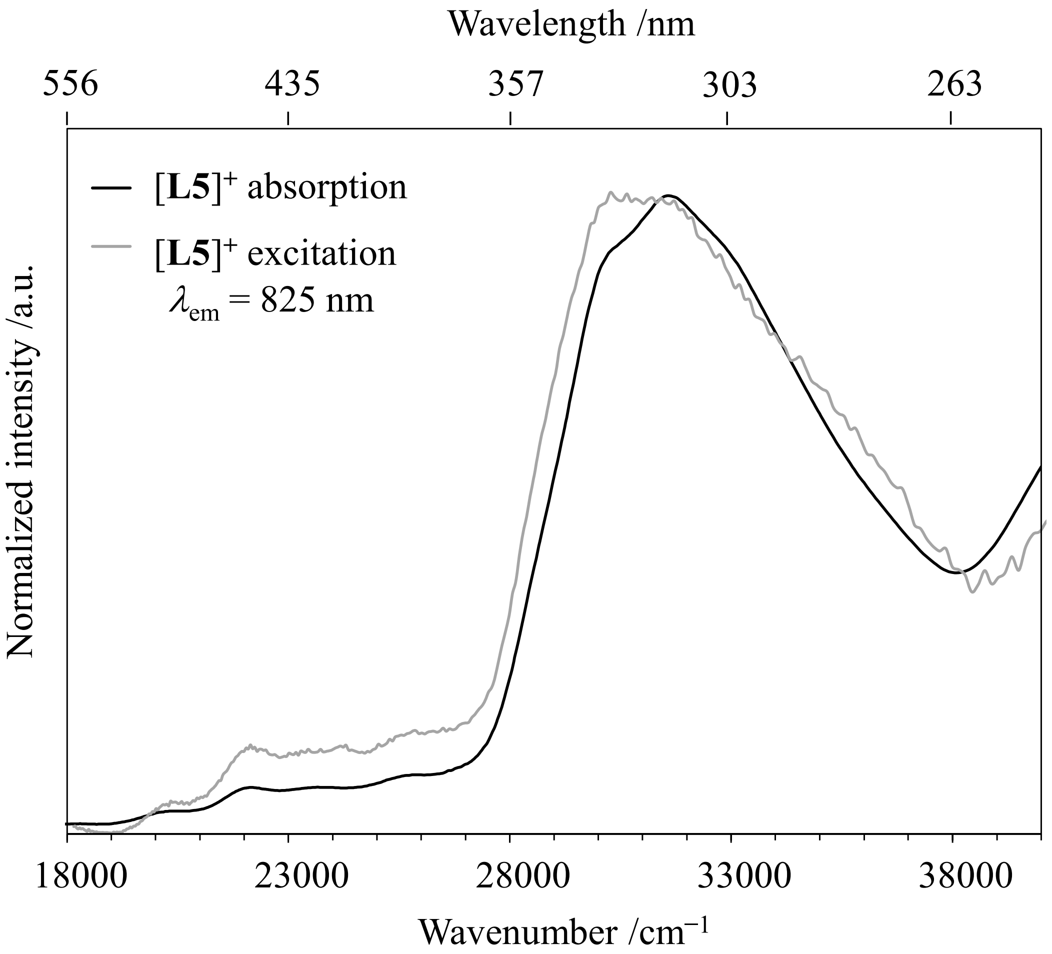

Fig. S23 Normalized excitation (λ_{em} = 825 nm) and absorption spectra of [**L5**]$^+$ (CH_3CN, 293 K).

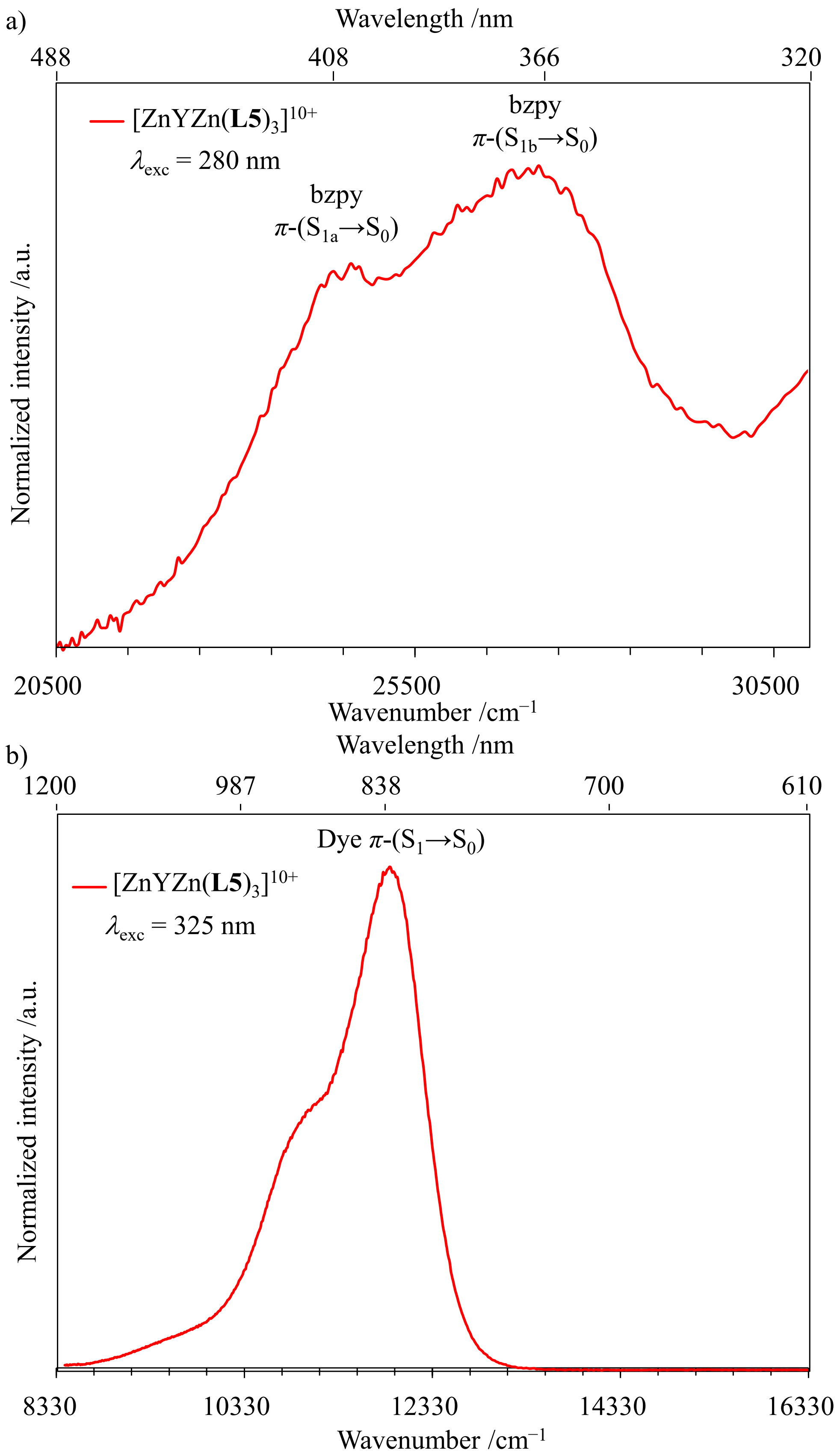

Fig. S24 Emission spectra of $[ZnYZn(\mathbf{L5})_3]^{10+}$ $4{\cdot}10^{-5}$ M (CH_3CN, 293 K) upon lamp excitation a) λ_{exc} = 280 nm and b) λ_{exc} = 325 nm.

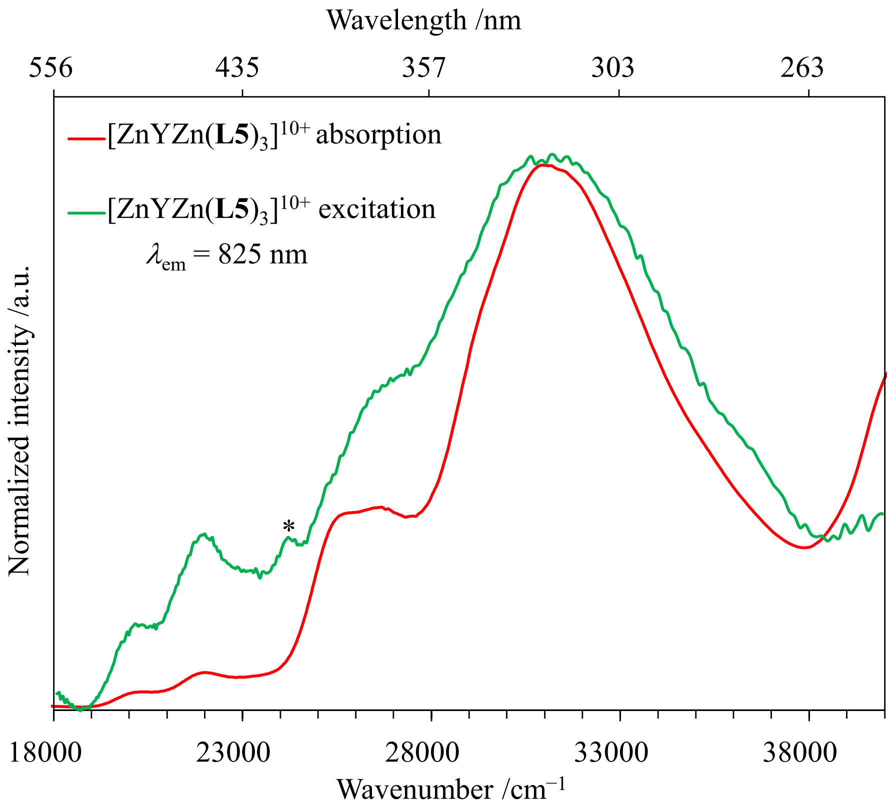

Fig. S25 Normalized excitation (λ_{em} = 825 nm) and absorption spectra of $[ZnYZn(\mathbf{L5})_3]^{10+}$ (CH_3CN, 293 K). * Corresponds to second-order Rayleigh band.

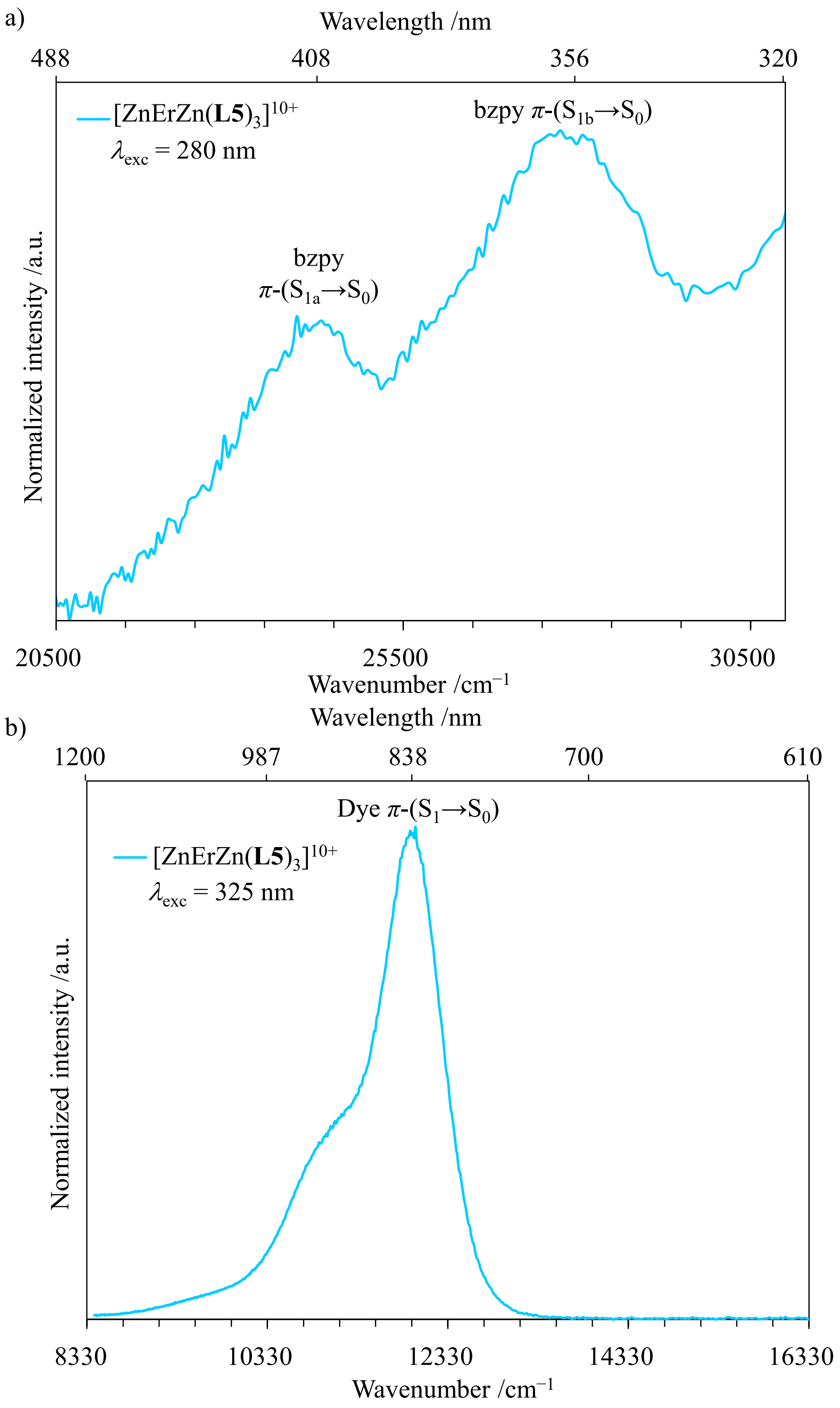

Fig. S26 Emission spectra of $[ZnErZn(\mathbf{L5})_3]^{10+}$ $4\cdot10^{-5}$ M (CH_3CN, 293 K) upon lamp excitation a) λ_{exc} = 280 nm and b) λ_{exc} = 325 nm.

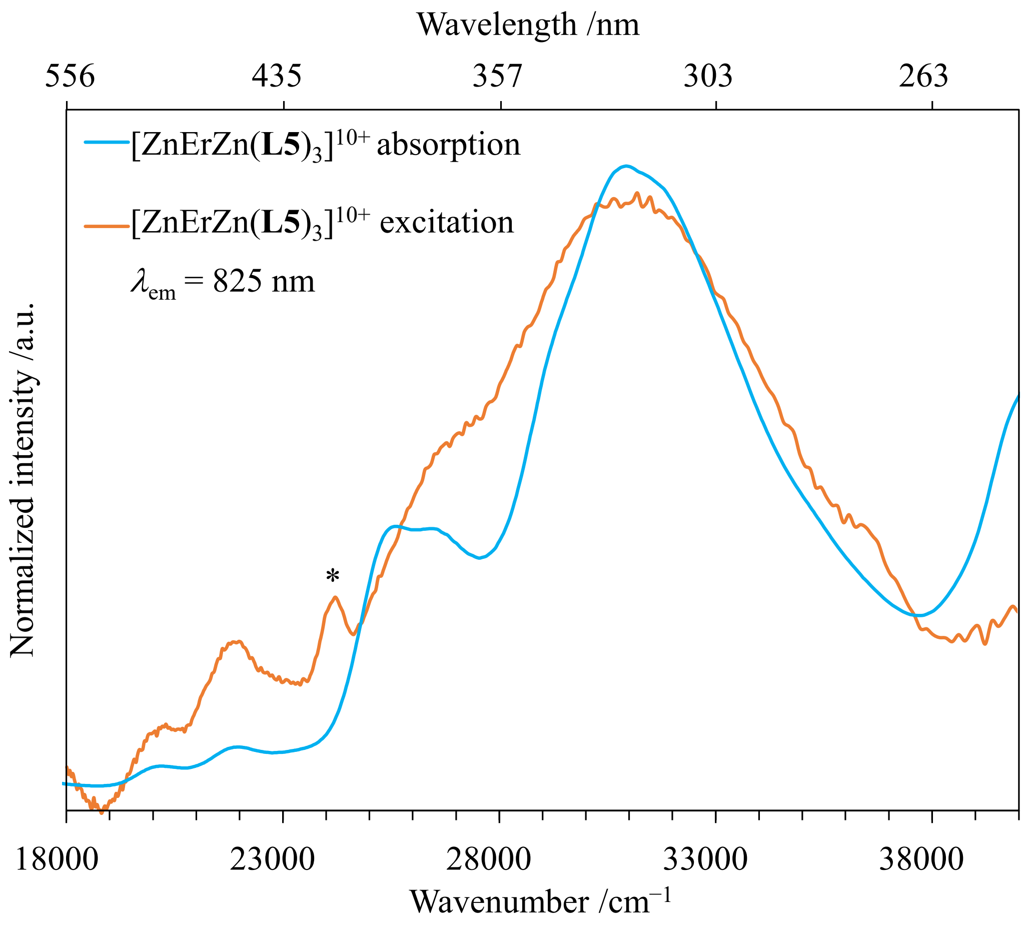

Fig. S27 Normalized excitation (λ_{em} = 825 nm) and absorption spectra of $[ZnYZn(\mathbf{L5})_3]^{10+}$ (CH_3CN, 293 K). * Corresponds to second-order Rayleigh band.

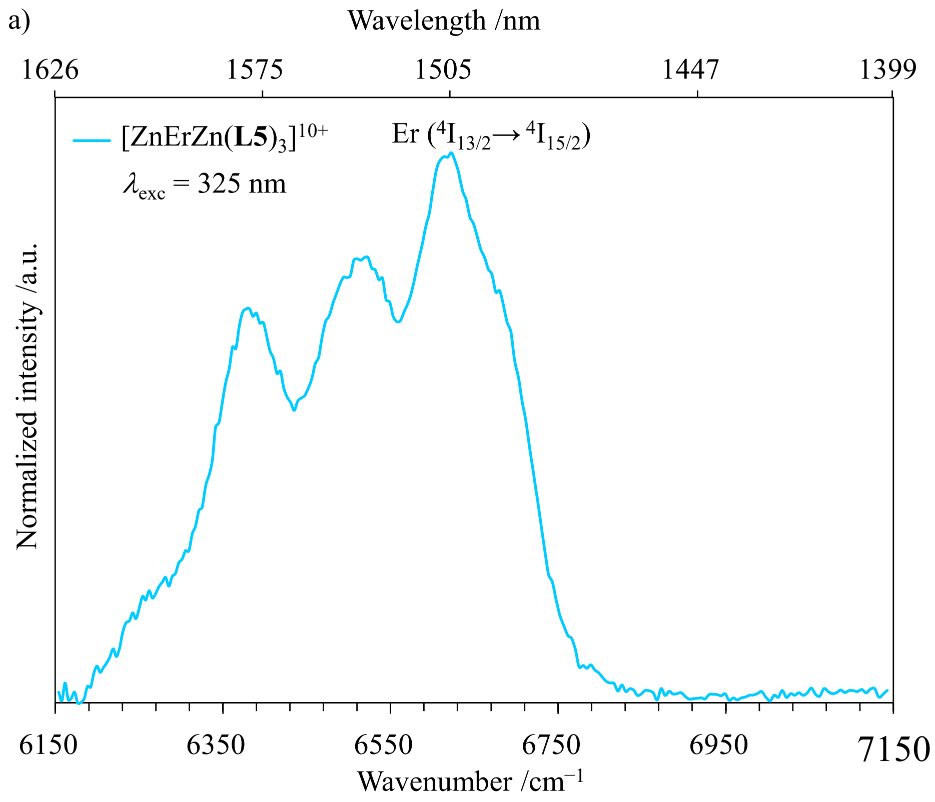

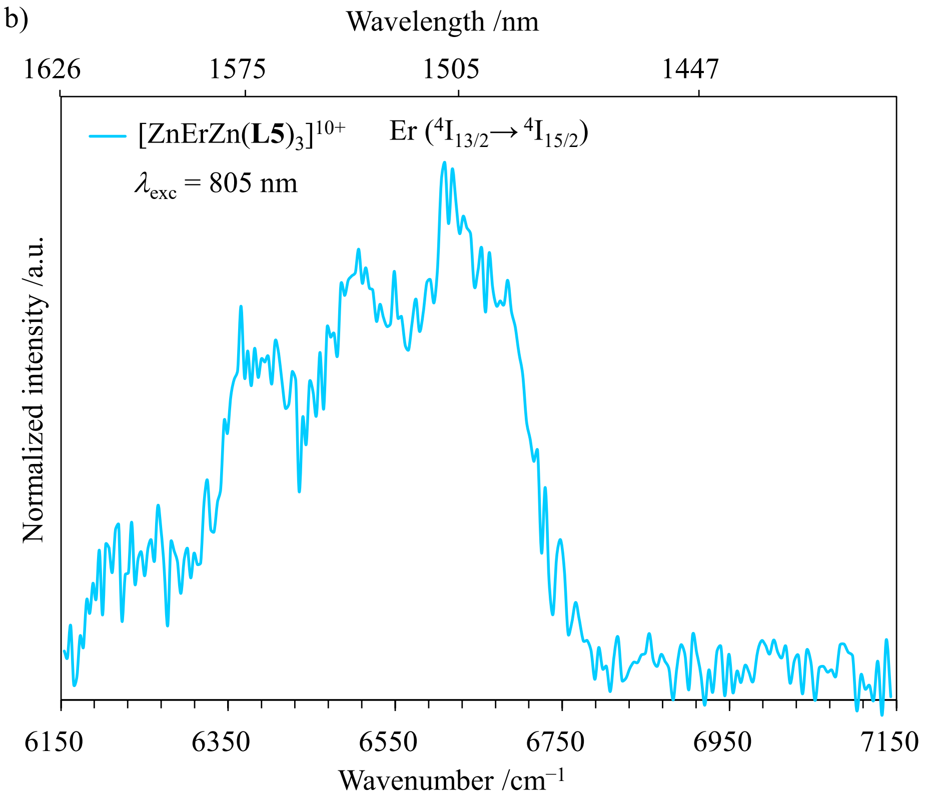

Fig. S28 Emission spectra of $[ZnErZn(\mathbf{L5})_3]^{10+}$ $4 \cdot 10^{-5}$ M (CH_3CN, 293 K) upon lamp excitation a) λ_{exc} = 325 nm and b) λ_{exc} = 805 nm.

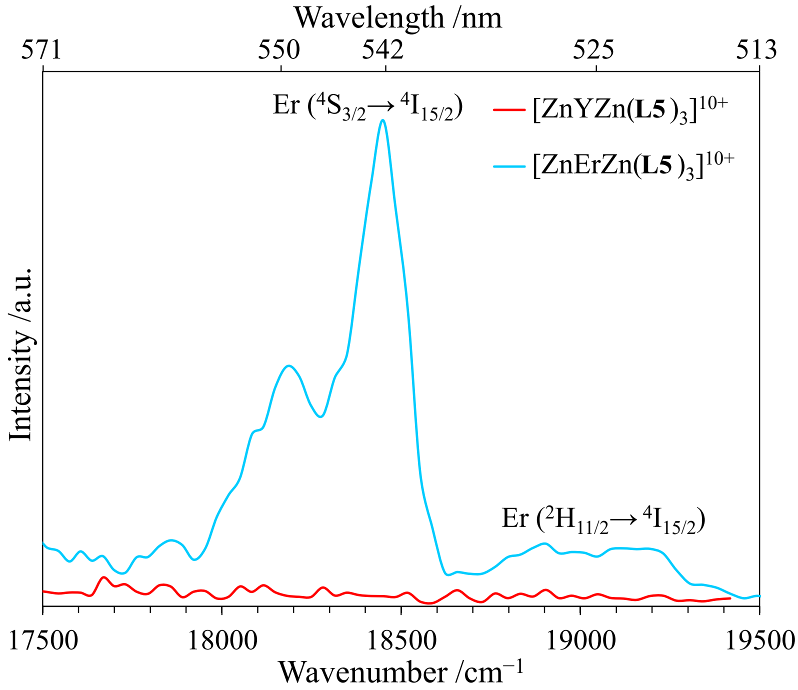

Fig. S29 Upconverted signal Er($^4S_{3/2}$→$^4I_{15/2}$) and Er($^2H_{11/2}$→$^4I_{15/2}$) of [ZnErZn(**L5**)$_3$]$^{10+}$ in acetonitrile solution (4·10^{-5} M, 293 K) traced in blue and of [ZnYZn(**L5**)$_3$]$^{10+}$ in acetonitrile solution (4·10^{-5} M, 293 K) traced in red, upon continuous wave laser excitation (λ_{exc} = 801 nm, P = 1.4 W·cm^{-2}).
